# Modular Donor‐Acceptor Diradicaloids Based on an Electron Deficient *N*‐Heteroacene Acceptor

**DOI:** 10.1002/smll.202510228

**Published:** 2026-02-10

**Authors:** Tanner L. Smith, Zhendian Zhang, Tanya A. Balandin, Paramasivam Mahalingam, Andrew H. Comstock, Anna M. Österholm, Michael K. Bowman, Molly M. Lockart, Guoxiang Hu, Jason D. Azoulay

**Affiliations:** ^1^ School of Chemistry and Biochemistry School of Materials Science and Engineering Center for Organic Photonics and Electronics Georgia Institute of Technology Atlanta Georgia USA; ^2^ Department of Chemistry and Biochemistry The University of Alabama Tuscaloosa Alabama USA; ^3^ Department of Chemistry and Biochemistry Howard College of Arts and Sciences Samford University Birmingham Alabama USA

**Keywords:** diradicaloid, donor‐acceptor, electronic structure, open‐shell, organic semiconductors

## Abstract

Conjugated organic molecules with open‐shell diradical character (*y*) possess two weakly paired electron spins interacting across their constituent *π*‐systems. These materials provide fundamental insight into the nature of electron pairing, enabling the utilization of the spin degree of freedom within emerging technologies. However, materials systems that synergistically offer high modularity, tunable *y*, high chemical stability, and interrelated (opto)electronic functionalities remain limited. Here, we report the facile synthesis of donor‐acceptor‐donor diradicaloids comprised of a central electron‐deficient 6,7,8,9‐tetrachloro‐[1,2,5]thiadiazolo[3,4‐*b*]phenazine acceptor flanked by electron‐rich thiophene‐based donors. Nuclear magnetic resonance and electron paramagnetic resonance spectroscopies, and theoretical investigations that account for the multiconfigurational nature of these species, connect a narrowing of the singlet‐triplet splitting (∆*E*_ST_), extension of *π*‐conjugation, and electronic correlations with the evolution of diradical character. These data demonstrate that the differences in structural, electronic, spin, magnetic, physicochemical, and transport properties of the materials can be modulated, while the inherent multireference nature of the electronic structure can be predicted using optimally tuned long‐range corrected Mixed‐Reference Spin‐Flip Time‐Dependent Density Functional Theory. These insights enable facile access to a broader range of open‐shell materials and facilitate the manipulation of important properties such as electronic structure, topology, exchange, and interrelated optoelectronic and transport functionalities.

## Graphical abstract

This work presents a modular approach to donor‐acceptor‐donor (D‐A‐D) diradicaloids that show systematic evolution of their energy gaps, singlet‐triplet splittings, and diradical character (*y*). The correlation of (opto)electronic, spin, and magnetic properties with high‐fidelity theoretical investigations provides clear connections between the degree of diradical character and electronic structure and functionality.

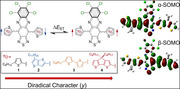

## 1 Introduction

Organic *π*‐conjugated materials with open‐shell diradical character are molecular species with an orbital manifold in which valence electron spins occupy nearly degenerate singly occupied molecular orbitals (SOMOs), whose exchange interaction (*J*) depends on the distance between the two spins [1, 2, 3, 4]. The unique electronic structures and spin‐correlated phenomena, which emerge from weakly paired or unpaired electron spins and the presence of magnetic moments coupled to light elements, offer richer categories of behavior than closed‐shell materials [5, 6, 7]. Such materials offer new fundamental insight into the nature of *π*‐bonding and electron pairing, which has brought about the creation of emerging functionalities that are defining a new generation of photonic, (opto)electronic, renewable energy, catalytic, magnetic, spintronic, quantum, and numerous other technologies [8, 9, 10, 11, 12, 13, 14, 15].

**FIGURE 1:**
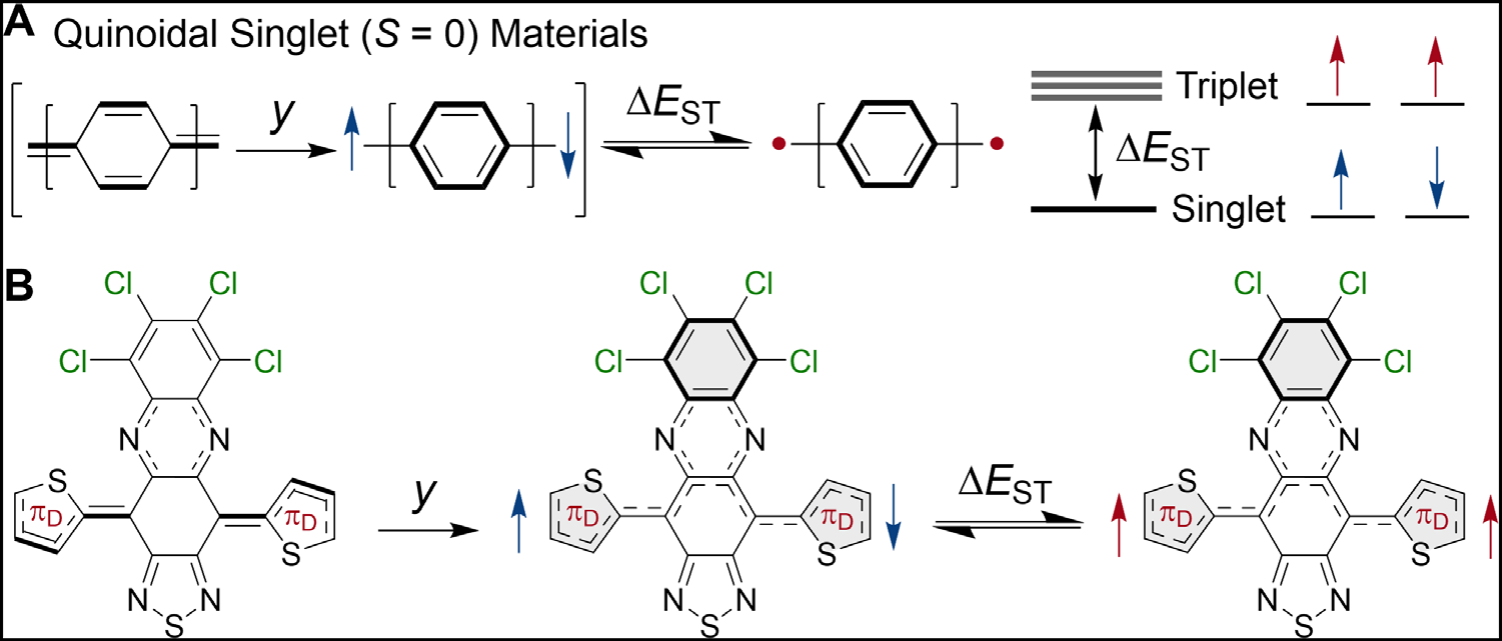
Molecular design of donor‐acceptor‐donor diradicaloids. (A) The resonance structures showing the closed‐ and open‐shell forms of Kekulé diradicaloids, characterized by the diradical character index (*y*), and the energy gap between the singlet ground state and thermally accessible triplet state (∆*E*_ST_). (B) Generalized molecular design and structure of the donor‐acceptor‐donor diradicaloids in this work.

The diradical character index (*y*) describes the degree of electron correlation, which ranges from 0 < *y* < 1, where a value of *y* = 0 refers to a closed‐shell electronic configuration, *y* = 1 to a diradical, and intermediate *y* values are referred to as diradicaloids [1, 2, 3]. The prevailing paradigm for the synthesis of these open‐shell species, spanning more than a century, has relied on embedding pro‐aromatic (quinoidal) structures within *π*‐conjugated frameworks, whereby the recovery of aromaticity reduces the covalency of a *π*‐bond and results in the adoption of an open‐shell ground state (i.e., Clar's rule). Various Kekulé diradicaloids based on this manifestation of Clar's rule have been developed, such as polycyclic aromatic hydrocarbons (PAHs) and related structures [16, 17], open‐shell graphene fragments [18], zigzag edge graphene nanoribbons (ZGNRs) [19], quinoidal oligothiophenes [20, 21], and many others [1, 3, 7]. The unifying features of these materials are structures that resemble a resonance hybrid of closed‐ and open‐shell forms with a singlet ground state (*S* = 0) and a reduced singlet–triplet gap (∆*E*_ST_) that allows for thermal population of the triplet state (*S* = 1) (Figure 1A) [16, 22, 23]. Emerging design paradigms include extending *π*‐conjugation to promote a synergistic narrowing of the bandgap (*E*_g_) and ∆*E*_ST_ to increase configurational admixing (HOMO‐LUMO mixing) and stabilize topological structures that facilitate the spatial distribution of α‐ and β‐SOMOs within the *π*‐conjugated backbone. The relative overlap of these SOMOs determines the degree of spin pairing, diradical character, and ground‐state electronic structure [1, 2, 24]. These emerging design guidelines have been demonstrated in correlated materials such as rylene ribbons and donor‐acceptor conjugated polymers [11, 12, 13, 25, 26, 27].

These delocalized diradicaloids share common characteristics including degenerate or near‐degenerate frontier molecular orbitals (FMOs), a considerable narrowing of the energy gap between spin states, weakened intramolecular electron‐electron pairing, and efficient spin polarization throughout the *π*‐conjugated structures, features that enable new functionality and high chemical stability [25, 26, 27]. This picture differs from electron‐rich PAHs, nanographenes, or ZGNRs in which localized SOMOs populate zigzag edges, or *π*‐systems that require isolation of open‐shell sites with bulky substituents that mask important *π*‐*π* and spin–spin interactions [7, 17, 18, 28, 29]. However, attaining narrow energy gaps in open‐shell organic molecules generally requires larger, more extensively conjugated *π*‐systems, which are often difficult to synthesize and isolate, and whose properties are difficult to control. This has been demonstrated by persistent difficulties in the solution‐based synthesis of highly conjugated open‐shell materials, which require extensive multistep syntheses, highly reactive open‐shell intermediates, and demonstrate poor solubility [18, 19].

Here, we demonstrate an approach that overcomes these limitations and enables facile synthetic access to *π*‐conjugated diradicaloids with narrow energy gaps (Figure 1B). Utilizing a heteroannulated 6,7,8,9‐tetrachloro‐[1,2,5]thiadiazolo[3,4‐b]phenazine acceptor flanked with progressively electron‐rich thiophene‐based donors gives rise to donor‐acceptor‐donor (D‐A‐D) frameworks that demonstrate a systematic narrowing of ∆*E*_ST_ and increase in *y*, which correlates with the spatial distribution of α‐ and β‐SOMOs within the *π*‐conjugated backbones. This remarkable capability in such small molecular systems is enabled by the highly electron‐deficient phenazinothiadiazole acceptor, which strongly withdraws electron density from the *π*‐conjugated backbones of these D‐A‐D materials. This provides the driving force for donor‐acceptor hybridization at shorter conjugation lengths than can be achieved using prototypical strong acceptors such as [1,2,5]thiadiazolo[3,4‐*g*]‐quinoxaline (TQ) or benzo[1,2‐*c*;4,5‐*c*']bis[1,2,5]thiadiazole (BBT) [30]. Such significant internal charge transfer character between electron‐rich donors and the phenazinothiadiazole acceptor leads to narrower energy gaps than achievable with TQ or BBT, enhanced HOMO‐LUMO admixing, adaptation of quinoidal bonding patterns, and a route for recovery of aromaticity via localization of electron density in the ancillary halogenated ring, features that promote open‐shell ground states [30]. In donor‐acceptor molecules and oligomers, internal charge transfer between intrachain donor and acceptor units leads to the delocalization of SOMOs. This feature provides thermodynamic stabilization and promotes exceptional chemical stability, even in the absence of sterically protective groups at open‐shell sites [31, 32]. Moreover, strong electronic correlations give rise to spin–spin, magnetic, and transport functions such as high electrical conductivity that are otherwise difficult to achieve. The well‐defined and compact nature of these materials systems enables high‐fidelity theoretical descriptions of their multiconfigurational nature using Mixed‐Reference Spin‐Flip Time‐Dependent Density Functional Theory (MRSF‐TDDFT) [33].

## 2 Results and Discussion

**FIGURE 2:**
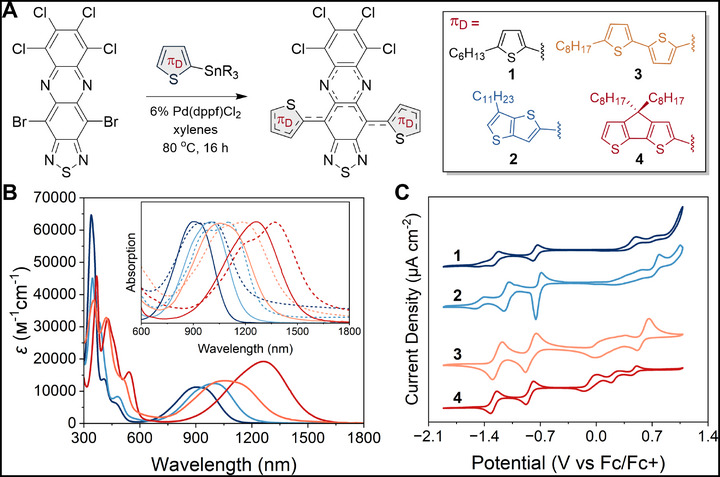
Synthesis of the D‐A‐D diradicaloids, absorption profiles, and electrochemical properties. (A) The molecular building blocks and Stille cross‐coupling used to synthesize compounds **1**–**4**. (B) Solution absorption spectra of **1**–**4** in chloroform (∼10^−5^ м, solid lines). Inset: Normalized absorption spectra of thin films (dashed lines) spin‐cast from chloroform or chlorobenzene onto quartz substrates. (C) Cyclic voltammograms (25 mV s^−1^) of **1**–**4** in degassed 0.1 м tetrabutylammonium hexafluorophosphate (Bu_4_NPF_6_) in dicholoromethane. All potentials are estimated from the third scan. Measurements were recorded from −0.42 to 1.08 V to −1.92 V under an argon atmosphere.

We designed and synthesized a series of D‐A‐D molecules comprised of a 6,7,8,9‐tetrachloro‐[1,2,5]thiadiazolo[3,4‐*b*]phenazine acceptor flanked by solubilizing, electron‐rich thiophene (**1**), thieno[3,2‐*b*]thiophene (**2**), 2,2'‐bithiophene (**3**), and 4*H*‐cyclopenta[2,1‐*b*:3,4‐*b*']dithiophene (**4**) donors (*π*_D_, Figure 2A). For all the D‐A‐D molecules considered, Density Functional Theory (DFT) shows that the open‐shell geometry is lower in energy than the closed‐shell configuration, indicating that the diradical form contributes to the ground state. Thus, we carried out calculations using various quantum mechanical approaches, finding that MRSF‐TDDFT with an optimally tuned long‐range corrected hybrid exchange–correlation functional (U)CAM‐B3LYP with the def2‐TZVP basis set correlates with experimental data (see Supporting Information section 3.7 for details) [34]. As further detailed below, the MRSF‐TDDFT method accounts for the inherent multireference nature of the diradicaloids’ electronic structure, strong electron correlation, and emerging properties, which otherwise pose significant challenges for traditional computational approaches.

The D‐A‐D complexes were synthesized from the coupling of excess monostannyl donors (2.5 eq.) with 4,11‐dibromo‐6,7,8,9‐tetrachloro‐[1,2,5]thiadiazolo[3,4‐b]phenazine [30]. Using [1,1′‐bis(diphenylphosphino)ferrocene]dichloropalladium(II) as the catalyst in xylenes at 80 °C and solubilizing donors comprised of (5‐hexyl‐2‐thienyl)trimethylstannane for **1,** (6‐undecylthieno[3,2‐*b*]thien‐2‐yl)tributylstannane for **2**, (5′‐octyl[2,2′‐bithiophen]‐5‐yl)trimethylstannane [35] for **3**, and (4,4‐dioctyl‐4*H*‐cyclopenta[2,1‐*b*:3,4‐*b*′]dithien‐2‐yl)trimethylstannane [36] for **4** resulted in soluble compounds. The stability of the compounds in ambient conditions enabled their purification using column chromatography, resulting in yields of 63% for **1**, 54% for **2**, 73% for **3**, and 33% for **4**. Thermogravimetric analysis (TGA) of **1**–**4** suggests that thermal decomposition begins at ∼375‐395 °C (Figure S8). The stability was also monitored by ^1^H NMR measurements of samples stored under ambient conditions for a period of 6 months, which show no changes to the spectra (Figures S9–S12).

The UV‐Vis‐NIR absorption spectra of **1**–**4** in chloroform are shown in Figure 2B. The absorption spectrum of **1** features a broad charge transfer peak with an absorption maximum (*λ*_max_) of 918 nm (*ε* = 10,363 м^−1^ cm^−1^) and a pronounced high‐energy peak centered at 340 nm. The *λ*_max_ of **2** is red‐shifted to 997 nm and rises in intensity (*ε* = 12,797 м^−1^ cm^−1^) relative to the high‐energy peak centered at 345 nm, which features a prominent shoulder at 382 nm. The spectrum of **3** displays a *λ*_max_ that is further red‐shifted to 1098 nm (*ε* = 13,261 м^−1^ cm^−1^) and the emergence of prominent peaks at 350 nm and 423 nm, which result from increased *π*‐extension and enhanced orbital mixing between donor and acceptor units when compared to **1** and **2**. Such strong donor‐acceptor hybridization is apparent in the spectrum of **4**, which features an intense charge transfer band with *λ*_max_ = 1263 nm (*ε* = 19,219 м^−1^ cm^−1^) and three pronounced high‐energy peaks at 365, 435, and 529 nm. These spectra demonstrate the ability of the electron‐deficient phenazinothiadiazole acceptor to induce charge transfer character and strong donor‐acceptor hybridization.

Thin films were prepared by spin‐coating chloroform or chlorobenzene solutions (10 mg mL^−1^) onto quartz substrates. These solid‐state absorption spectra show two significant changes relative to the solution spectra: a redshift of *λ*_max_ that is not observed in the high‐energy band, and a change in the shape of the absorption profile consistent with distinct 0‐0 and 0–1 vibrational bands (Figure 2B; Figure S13). The spectrum of **1** displays a solid‐state absorption profile with *λ*_max_ = 1020 nm, which progressively redshifts in going from **2** (*λ*_max_ = 1096 nm) to **3** (*λ*_max_ = 1189 nm) and **4** (*λ*_max_ = 1372 nm). The changes in the solid‐state absorption spectra arise from strong intermolecular coupling, which leads to bathochromatic shifts of the absorption bands toward longer wavelengths [37]. Compound **4** shows the most prominent increase in intensity of the 0‐0 peak relative to the 0–1 peak (*I*_0–0_/*I*_0–1_ = 1.2), consistent with enhanced aggregation compared to **1**, **2**, and **3**, which can be associated with the increased planarity and *π*‐delocalization of the fused 4*H*‐cyclopenta[2,1‐*b*:3,4‐*b'*]dithiophene donors. The optical energy gap (*E*_g_^opt^) was estimated from the absorption onset of the thin films, which progressively decreases moving across the series from **1** (1.01 eV), to **2** (0.95 eV), to **3** (0.83 eV), and to **4** (0.75 eV).

The electrochemical properties were evaluated using cyclic voltammetry in a 0.1 м solution of tetrabutylammonium hexafluorophosphate (Bu_4_NPF_6_) in dichloromethane (Figure 2C). In open‐shell systems, the first oxidation wave should correspond to the removal of an electron from the SOMO. Compared to their closed‐shell analogs, the oxidation potential for singlet diradicaloids is generally lower due to the presence of unpaired electrons. The cyclic voltammogram (CV) of **1** shows two oxidation waves at 0.43 and 0.74 V versus Fc/Fc^+^, indicative of stepwise removal of two electrons from its SOMO and another frontier molecular orbital. Upon reduction, two consecutive reduction processes are observed at −0.76 and −1.13 V. From the first oxidation and reduction processes, we estimate an electrochemical gap of 1.19 eV. Compound **2** displays similar amphoteric redox behavior, albeit with a third reduction wave at 1.47 V. The slightly lower onset of oxidation (0.41 V) is attributed to the more extended conjugation of the thienothiophene donor, and results in a lower estimated electrochemical gap of 1.13 eV. Compounds **3** and **4** display progressively smaller electrochemical gaps of 0.91 and 0.75 eV, respectively, which can be attributed primarily to their low oxidation potentials due to the progressively more *π*‐conjugated donor units. It is worth noting that **4** exhibits three distinct oxidation processes, suggesting its high‐lying occupied molecular orbitals and extended *π*‐conjugation promote stabilization of the (tri)cation [38, 39]. The observed trend in electrochemical gap is consistent with the fusion of progressively electron‐rich donors to the acceptor, which stabilizes the SOMO energies with minimal impact on the SUMOs (Figure S14).

The transport properties were assessed by fabricating bottom gate, bottom contact field effect transistors (FET) with the architecture Si/SiO_2_ (300 nm)/Au (60 nm) (see Supporting Information section 3.4 for details). The transfer current‐voltage (*I*–*V*) characteristics were obtained by sweeping the gate bias (*V*_g_) from 60 to ‐60 V under a source‐drain bias (*V*_d_) of −60 V. Devices of compound **1** showed no measurable transport properties. Compounds **2** and **3** showed p‐type FET behavior, with hole mobilities (*µ*_h_) of 4.0 × 10^−4^ and 5.3 × 10^−4^ cm^2^ V^−1^ s^−1^, respectively (Figure S15). Compound **4** also shows p‐type‐dominated FET behavior, with a higher hole mobility (*µ*_h_) of 4.15 × 10^−1^ cm^2^ V^−1^ s^−1^ (Figure S15). The transfer curve of **4** does not demonstrate an off‐state, indicating the presence of free carriers. Thus, the room‐temperature conductivity (*σ*_RT_) of **4** was measured without the application of a gate bias, which shows linear current–voltage (*I*–*V*) characteristics with *σ*_RT_ = 6.17 × 10^−2^ S cm^−1^ (Figure S15). The Ohmic transport in **4** arises from its open‐shell character and narrow energy gap, which results in a low energetic barrier for thermal activation of free charge carriers, while strong intermolecular coupling enables extensive charge delocalization [10, 15, 40, 41].

**FIGURE 3:**
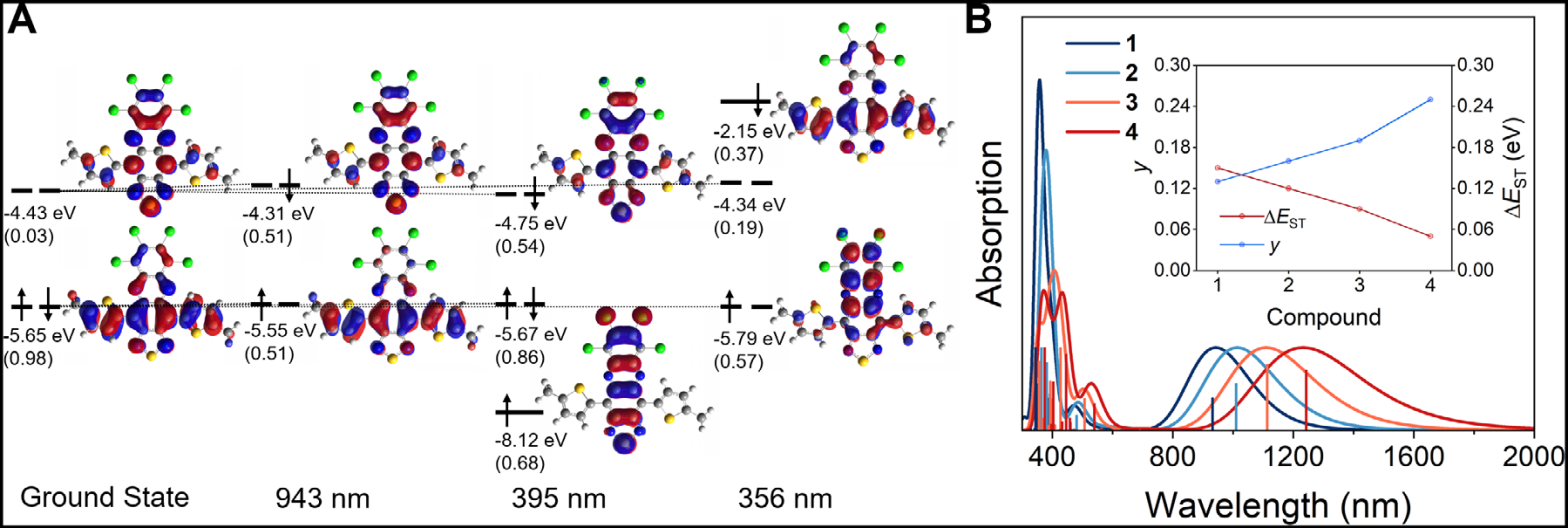
Theoretically determined optical properties at the MRSF‐TDDFT level of theory with the (U)CAM‐B3LYP functional and def2‐TZVP basis set. (A) Dyson Orbital (DO) diagram of the major electronic transitions in the absorption spectrum of **1**, with the normalized occupancies in parentheses. (B) Absorption spectra of **1**–**4** predicted at the MRSF‐TDDFT level of theory together with the transitions represented as bars, in which the heights are proportional to the oscillator strength. Inset: Broken‐symmetry (BS)‐DFT predicted values for *y* plotted against the MRSF‐TDDFT predicted values of Δ*E*_ST_.

The simulated absorption spectra and optical transitions calculated using MRSF‐TDDFT are shown in Figure 3. Dyson Orbitals (DO) give both a chemically intuitive and quantitative description of the excited state transitions, which provide excitation energies that correlate with the absorption spectra of **1**–**4** and enable further insight into the major electronic transitions (see Supporting Information section 3.7 for details) [42, 43]. As depicted in Figure 3A, the low‐energy excitation in **1** is an intramolecular charge transfer transition between the donor *π* orbital and the acceptor π* orbital, corresponding to excitation of the open‐shell singlet [42]. The MRSF‐TDDFT absorption spectrum of **1** predicts a *λ*_max_ = 943 nm (*f* = 0.40) that originates from the SOMO(α) → SUMO(α) and SOMO(β) → SUMO(β) transitions (Figure 3B), in good agreement with the solution spectrum with *λ*_max_ of 918 nm (*ε* = 10,363 м^−1^ cm^−1^). The most prominent transitions in the high‐energy band correspond to a SOMO → LUMO+3 excitation at 356 nm (*f* = 1.0) and a HOMO‐5 → SUMO excitation at 395 nm (*f* = 0.39) that is localized on the acceptor. The predicted absorption profiles of **2**, **3**, and **4** similarly correlate with their respective solution spectra, which show progressively red‐shifted charge transfer bands with *λ*_max_ = 1014 nm (*f* = 0.56) in **2**, *λ*_max_ = 1110 nm (*f* = 0.80) in **3**, and *λ*_max_ = 1232 nm (*f* = 0.72) in **4**. Furthermore, the larger oscillator strengths of **3** and **4** compared to **1** are consistent with the increase in *ε* from **1** → **4** displayed in the solution spectra, reflecting enhanced internal charge transfer across the series. The high‐energy band of **2** includes an additional transition at 385 nm (*f* = 0.82) that MRSF‐TDDFT attributes primarily to a SOMO → LUMO+1 excitation, which is red‐shifted to 423 nm (*f* = 0.90) in **3** and 435 nm (*f* = 0.91) in **4**. This further demonstrates progressively enhanced donor‐acceptor hybridization from **1** → **4**, resulting in a concomitant narrowing of *E*_g_ that provides the driving force for increased diradical character due to configuration mixing between the frontier MOs [25, 39].

MRSF‐TDDFT has been shown to overcome challenges associated with the multiconfigurational nature of diradicals, enabling accurate predictions of Δ*E*_ST_ [33, 34, 44]. This theoretical approach predicts that all the D‐A‐D materials considered have open‐shell singlet (*S* = 0) ground states with two electrons occupying (near) degenerate frontier SOMOs (see Supporting Information section 3.7 for details). The contribution of the open‐shell resonance form to the ground‐state structure was calculated from the occupation number of the lowest occupied natural orbitals, giving values of *y* = 0.13, 0.16, 0.19, and 0.25 for **1**, **2**, **3**, and **4**, respectively. This trend correlates with increased diradical character upon narrowing *E*_g_^opt^ and is accompanied by a concomitant narrowing of Δ*E*_ST_ from ‐0.154 → ‐0.053 eV in going from **1** → **4** (Table S6).

**FIGURE 4:**
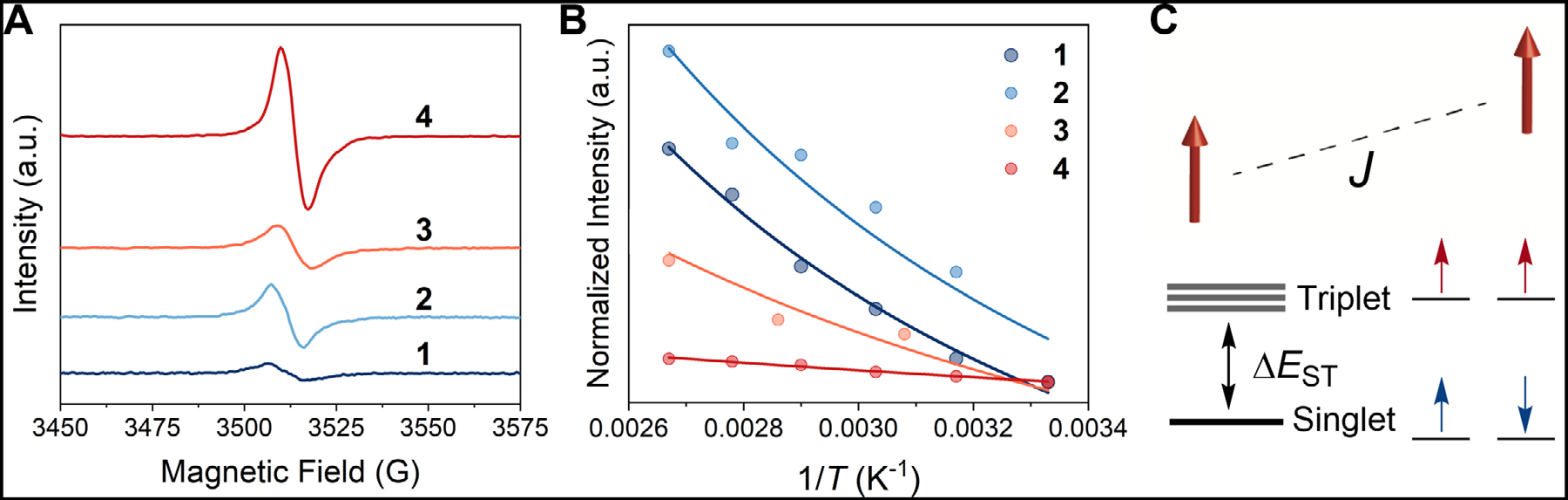
EPR spectra of the D‐A‐D diradicaloids. (A) CW EPR (X‐band) spectra of powder samples at room temperature. (B) Temperature‐dependent fits of the solution spectra to the Bleaney‐Bowers equation of **1**, **2**, and **4** in toluene and **3** in bromobenzene (∼10^−5^ м) between 300–375 K. (C) The energy gap between the singlet ground state and the thermally populated triplet state of the D‐A‐D molecules, which is proportional to the exchange interaction (Δ*E*_ST_ *= 2J*).

Continuous wave (CW) electron paramagnetic resonance (EPR) measurements of the samples confirm the open‐shell nature of the compounds (Figure 4A). At room temperature, the strong intermolecular interactions of each triplet with its neighboring singlet and triplet molecules in the solid‐state collapse the EPR spectra to single, nearly featureless broad lines 7 to 12 G wide with *g*‐factors (*g*) from 2.0045 to 2.0041 (Figure 4A). Superconducting quantum interference device (SQUID) magnetometry of these powders also reveals strong intermolecular spin–spin coupling among these open‐shell species (Figure S19), behavior that is common amongst strongly interacting organic diradicals [7, 45, 46]. These SQUID, EPR and thin film absorption spectra from highly‐aggregated pure samples of **1**–**4** reveal little about individual molecular properties. Thus, we prepared dilute solutions (∼10^−5^ м) of **1**–**4** in a range of solvents in order to study the isolated molecules.

Variable temperature (VT) EPR of dilute solutions of **1**–**4** demonstrate an increase in signal intensity with increasing temperature, consistent with a triplet state thermally populated from a singlet ground‐state. To quantitatively determine ∆*E*_ST_, the integrated EPR intensities (*I*_EPR_) were measured as a function of reciprocal temperature (*T* ^−1^) from 300 to 375 K and fit by the Bleaney–Bowers equation (Figure 4B), giving Δ*E*_ST_ of ‐0.121, ‐0.120. ‐0.100, and ‐0.048 eV for **1**, **2**, **3**, and **4**, respectively [47]. These values are in excellent agreement with the respective MRSF‐TDDFT predicted values of −0.154, −0.125, −0.093, and −0.053 eV. The VT EPR confirms the low‐spin ground state with small intramolecular antiferromagnetic coupling (*J* < 0) and thermal population of the triplet state with ferromagnetic interactions between unpaired spins (Figure 4C). The decrease in Δ*E*_ST_ correlates with the increase in *y* and progressive spatial separation of spins.

**FIGURE 5:**
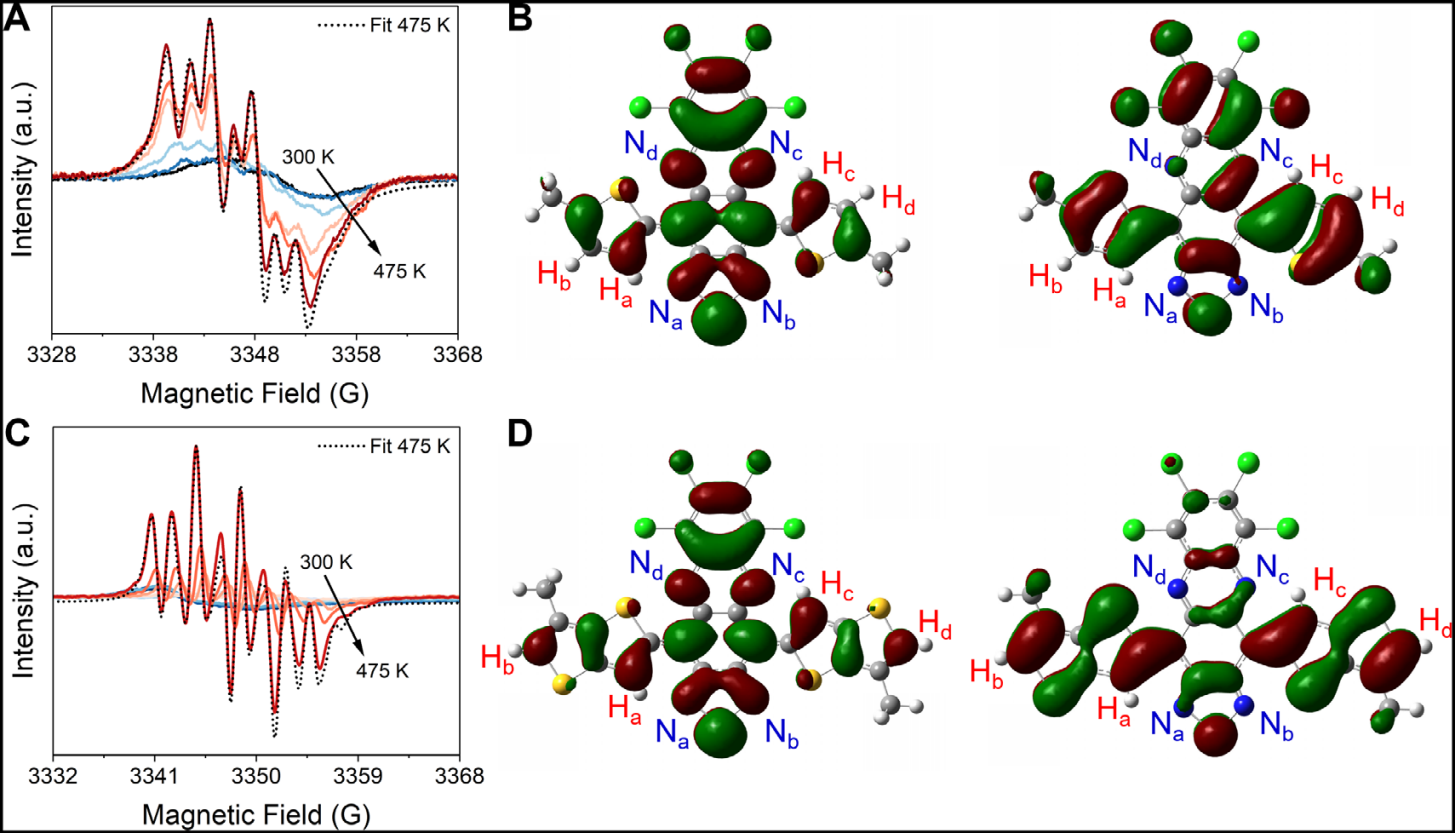
Variable temperature EPR of 1 and 2 and electronic structures of the triplets. (A) EPR spectra of 1 in CN (1 mg ml^−1^) from 300–475 K and simulation of the 475 K spectrum (dotted line). (B) Triplet α‐ and β‐SOMOs of **1**. (C) EPR spectra of **2** in CN (1 mg ml^−1^) from 300–475 K and simulation of the 475 K spectrum (dotted line). (D) Triplet α‐ and β‐SOMOs of **2**.

The EPR spectra of **1** and **2** in 1‐chloronaphthalene (CN) show the emergence of hyperfine splittings from ^14^N and ^1^H nuclei (Figure 5). The increasing resolution with temperature from 300 to 475 K results from an increased rate of molecular tumbling, which reduces spectral broadening from anisotropic hyperfine and zero‐field splitting (ZFS) interactions [48]. This increased resolution enables measurement of hyperfine coupling constants (HFCCs) that provide insight into the triplet SOMOs and spin density distributions. The spectrum at 475 K for **1** in CN is consistent with hyperfine couplings of 15.65 and 10.04 MHz to two unique ^14^N nuclei, and 2.26 and 1.86 MHz to two unique ^1^H nuclei (Figure 5A). The largest hyperfine couplings in **1** are associated with the two ^14^N nuclei on the phenazine ring of the acceptor (N_c_ and N_d_) and the thiophene ^1^H nuclei proximal to the acceptor core (H_a_ and H_c_), which have the most substantial triplet spin densities (Figure 5B; Figure S36). The remaining ^14^N and ^1^H nuclei in **1**, and each additional compound, are unresolved because their HFCCs are smaller than the linewidth of any of the lines in the spectra, which is limited by the rotational correlation time of the molecules. The spectrum of **2** at 475 K in CN shows hyperfine splittings consistent with coupling to two unique ^14^N nuclei with HFCCs of 14.43 and 8.66 MHz and one ^1^H nucleus with an HFCC of 5.90 MHz (Figure 5C). When compared to **1**, the smaller HFCCs for the ^14^N nuclei and larger HFCC for the ^1^H nucleus in **2** can be attributed to enhanced delocalization that depletes spin density from the acceptor core and distributes it onto the donor (Figure 5D; Figure S37) [49, 50]. The lack of symmetry inherent in the EPR spectra are consistent with DFT calculations, which predict HFCCs that are inequivalent for all of the ^14^N and ^1^H nuclei (Tables S10 and S11). The asymmetry can be understood by analyzing the optimized triplet geometries of **1** and **2**, which show differing conformations of the flanking thiophene donors resulting in distinct delocalization of the triplet SOMOs (Figure 5B,D).

The EPR spectra of **1** and **2** show a high‐field shoulder and slight shift in the centroid of the spectra as the temperature is varied, which suggests the presence of intermolecular interactions that persist even at the highest temperatures measured [51]. These data are consistent with spectral simulations (Figures S25 and S26) and supported by VT UV‐Vis measurements that show strong intermolecular interactions in CN, particularly for **2**, whose spectra remain nearly unchanged with increasing temperature and in solvents with better solubilizing power such as chloroform and toluene (Figures S27–S30). A comparison of the optimized singlet and triplet geometries reveals that the triplet is significantly more planar than the singlet (Figures S44 and S45), with an almost fully coplanar conformation that would lead to stronger intermolecular interactions. The solution spectra of **3** and **4** show similar features to **1** and **2**, albeit with broader peaks on account of their larger structures, slower rotation, poorer averaging of anisotropic interactions, and greater triplet spin delocalization, accounting for their poor resolution in a range of solvents (Figures S22–S24). DFT predicts hyperfine coupling to eight and six unique ^1^H nuclei for **3** and **4**, respectively, with smaller albeit similar HFCCs for ^14^N nuclei and the ^1^H nuclei proximal to the acceptor compared to **1** and **2** (Tables S12 and S13), consistent with more extensive triplet spin density delocalization across the progressively *π*‐extended donor units (Figures S40 and S41). The ^14^N couplings from the acceptor are similar across all four compounds, while the ^1^H couplings that arise from the donor moiety have much larger variations across the series (Tables S10–S13). These trends indicate that the SOMOs are similar on the acceptor but depend strongly on the structure of the donor, providing an opportunity for fine‐tuning the electronic and spin properties of this family of diradicaloids.

Variable temperature ^1^H NMR experiments provide additional insight into the evolution of Δ*E*_ST_ and spin distribution throughout the series. Compounds **1** and **2** display sharp, well‐defined aromatic peaks in their ^1^H NMR spectra from 260 to 348 K, consistent with large Δ*E*_ST_ and minimal population of the triplet in this temperature range (Figures S32 and S33), consistent with EPR. The peak attributed to H_a_ shifts significantly from 8.23 to 8.92 ppm in **1** and from 7.97 to 9.05 ppm in **2**, which can be attributed to temperature‐dependent hyperfine shifts that arise from spin polarization of the paramagnetic triplet (Figures S32 and S33) [21, 52, 53]. The delocalization of spin density onto the acceptor upon thermal population of the triplet state depletes electron density from the donor, deshielding the thiophene protons (Figures S36 and S37). This delocalization of spin density is consistent with the strong hyperfine couplings to both ^14^N nuclei on the acceptor and proximal ^1^H nuclei as observed in the EPR of **1** and **2**.

**FIGURE 6:**
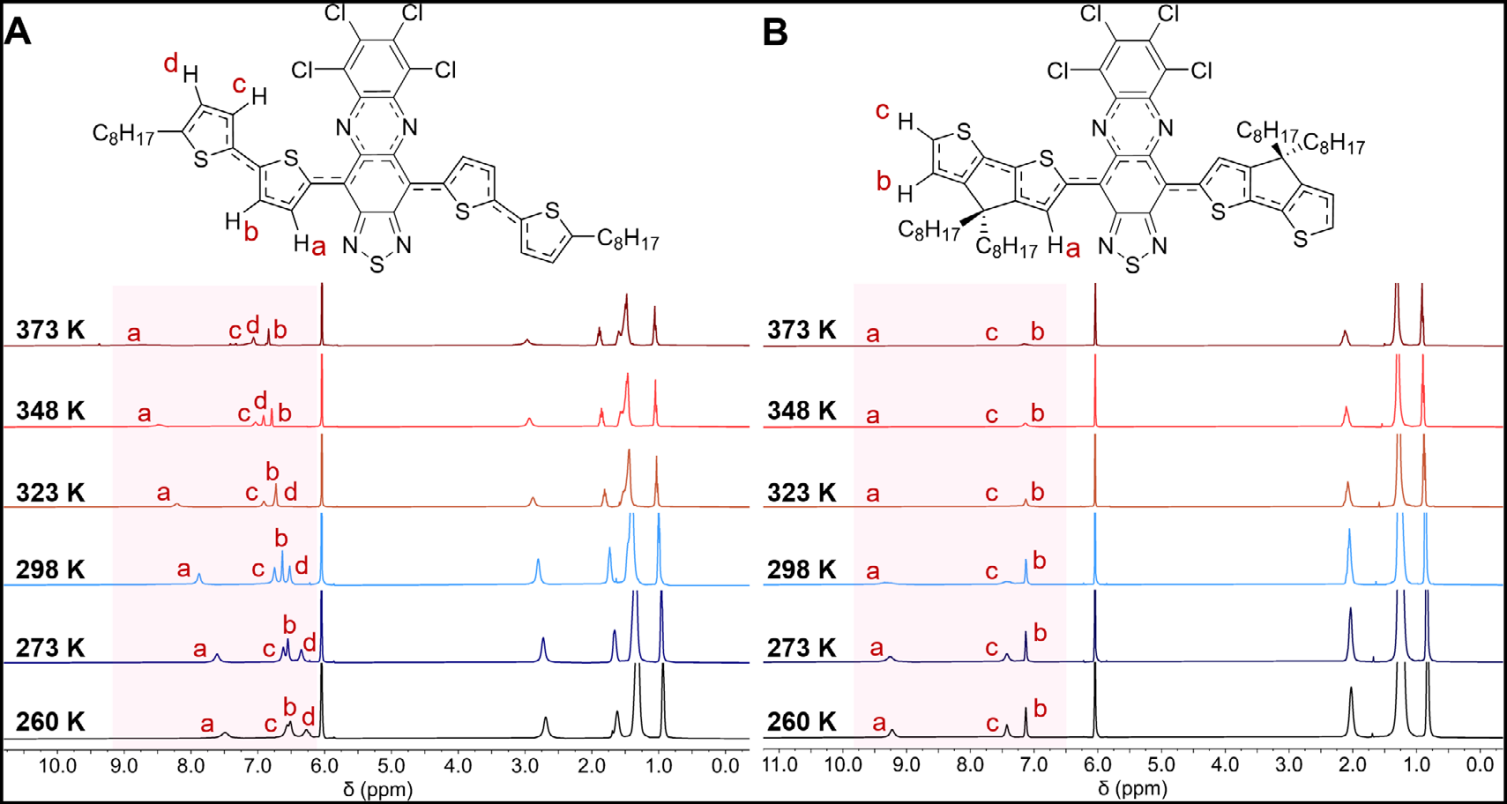
Temperature‐dependent NMR spectra of 3 and 4. (A) ^1^H NMR spectra of 3 in 1,1,2,2‐tetrachloroethane‐*d*_2_ between 260–373 K. (B) ^1^H NMR spectra of **4** in 1,1,2,2‐tetrachloroethane‐*d*_2_ between 260‐373 K.

Compound **3** shows broad aromatic peaks between 6.0 and 7.5 ppm at 260 K due to aggregation consistent with alkyl group anisotropy (Figure 6A). Upon heating to 298 K, these peaks sharpen, and then progressively broaden as the temperature is increased from 298 to 373 K due to thermal population of the triplet state. Broadening of all the aromatic protons is consistent with delocalization of the paramagnetic triplet across the bithiophene donor, however, the more significant peak broadening of H_a_ indicates a larger triplet density on the carbon adjacent to the interannular bond, consistent with DFT (Figure S38). Compound **3** also displays significant hyperfine shifts upon warming from 260 to 373 K (Figure 6A; Figure S34), with the largest shifts occurring on H_a_ (7.49 to 8.68 ppm) and on H_d_ (6.27 to 7.06 ppm). The spectrum of **4** shows the same trend with broad peaks corresponding to H_a_ and H_c_ at 298 K that sharpen as the temperature is lowered to 260 K (Figure 6B; Figure S35), consistent with a smaller Δ*E*_ST_ when compared to **1**, **2**, and **3**. The broad H_c_ peak in **4** implies a large population of spin density on the peripheral carbons of the donor, consistent with more pronounced delocalization to opposite sides of the molecule.

**FIGURE 7:**
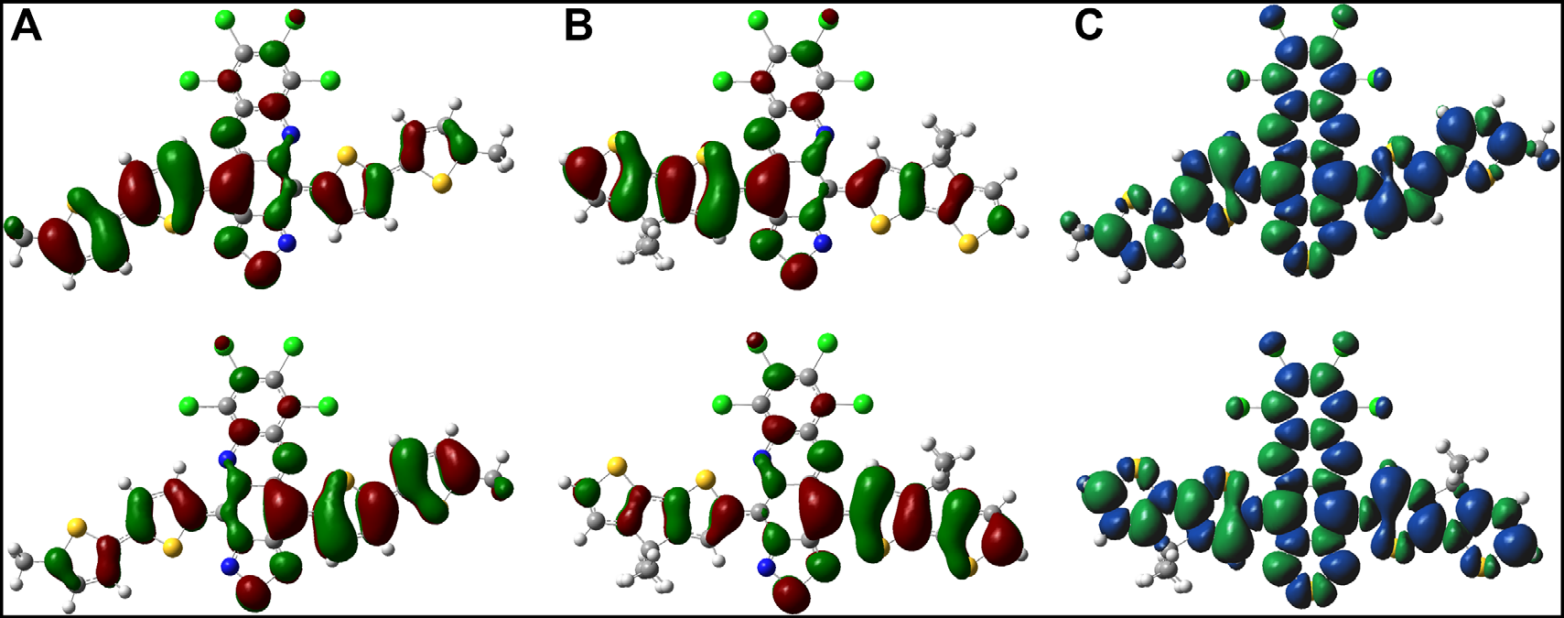
Electronic structure of the singlet ground states of 3 and 4. Singlet α‐ and β‐SOMOs of (A) compound **3** and (B) compound **4** modeled using BS‐DFT with the (U)CAM‐B3LYP functional and def2‐TZVP basis set. The green and red surfaces represent positive and negative signs of the MO at isovalue = 0.02 au, respectively. (C) Spin density distributions of the singlet states of **3** (top) and **4** (bottom). The blue and green surfaces represent positive and negative contributions of the spin density at an isovalue = 0.04 au.

The progression of open‐shell character and correlation with experimental results can be better understood by analyzing the evolution of ground state SOMOs and spin density distributions throughout the series. Molecular orbital analysis of the singlet ground states of **1** and **2** show significant spatial overlap of the α‐ and β‐SOMOs (Figures S40 and S41), which become progressively more segregated in **3** and **4**, such that the α‐ and β‐spins are primarily localized on opposite donor moieties of the D‐A‐D frameworks (Figure 7A,B). The spatial separation of antiferromagnetically coupled spins reduces bond covalency, decreases electron repulsion, and increases *y* across the series [22, 24, 25, 54]. All derivatives display significant spin polarization and spin delocalization across the *π*‐conjugated backbones (Figure 7C; Figures S40 and S41), providing thermodynamic stabilization despite the absence of sterically protective groups at the sites with large spin densities [31, 32, 55]. The triplet spin densities are similarly distributed onto the donors, albeit with delocalization of spin density onto the acceptor consistent with hyperfine coupling to the ^14^N nuclei observed in EPR. The presence of large triplet spin densities on the carbon adjacent to H_a_ results in the broad peaks corresponding to H_a_ in the VT ^1^H NMR spectra of **2**, **3**, and **4,** and to the large HFCCs observed for H_a_ and H_c_ in compounds **1** and **2**. Similarly, the peak‐broadening observed for H_c_ in **3** and **4** is consistent with delocalization of triplet spin density onto the acceptor and periphery of the donors, which coincides with progressive localization of the triplet β‐SOMOs onto the donors.

**FIGURE 8:**
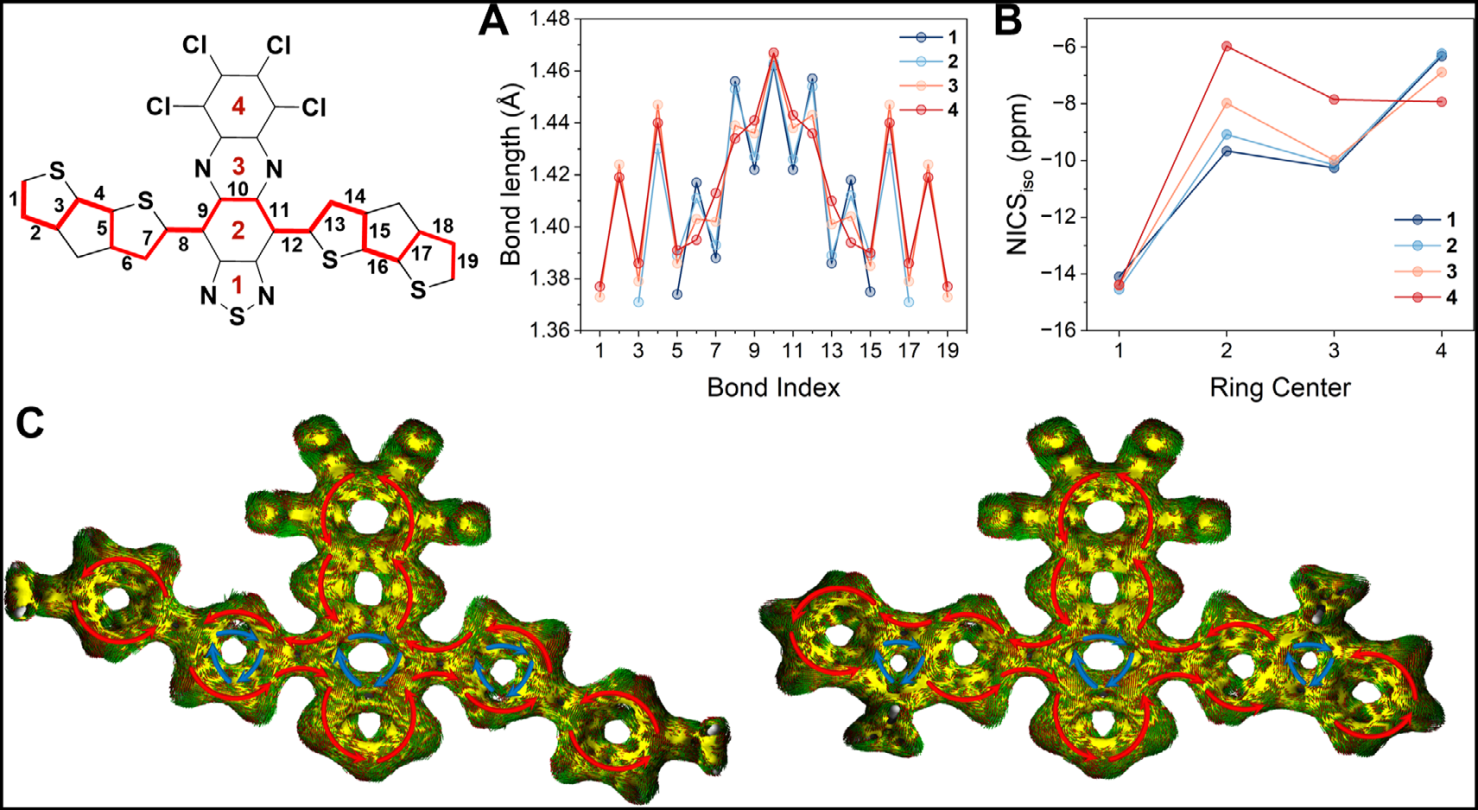
Theoretically determined bond lengths and aromaticity of the D‐A‐D molecules. (A) Bond length analysis and (B) NICS_iso_ values computed for each ring on the acceptor core in the singlet ground states of **1**–**4** modeled using BS‐DFT with the (U)CAM‐B3LYP functional and def2‐TZVP basis set. (C) Anisotropy of the induced current density (ACID) plots for **3** and **4**. The ring current is indicated by the current density vectors plotted on the ACID isosurface (red: clockwise, diatropic; blue: counterclockwise, paratropic).

Bond‐length alternation (BLA) analysis and nucleus independent chemical shift (NICS) calculations were carried out to correlate changes in molecular structure with the evolution of diradical character from **1** to **4**. The interannular bond between the donor and acceptor is reduced in length from 1.456 Å in **1** to 1.434 Å in **4**, consistent with increased double bond character toward the quinoidal form on the center ring (Figure 8A). This transition is further corroborated by a concomitant decrease in NICS_iso_ values on the central ring of the acceptor from an initial value of −9.67 ppm for **1** to −5.97 ppm in **4** (Figure 8B), consistent with significant quinoidal contribution to the ground state structures [27, 56]. The quinoidal shifts on rings 2 and 3 of the acceptor are accompanied by increasingly aromatic shifts on ring 4, with NICS_iso_ values that decrease from −6.32 ppm for **1** to −7.93 ppm in **4**, which demonstrates the recovery of aromatic resonance energy within the peripheral tetrachlorophenazine units [30]. The progression of quinoidal character is further evident by increased BLA on the donor moieties in **3** and **4**, in which elongation of bond 7 is accompanied by a contraction of bond 6, and is accompanied by more positive NICS_iso_ values on the peripheral thiophene rings (Tables S14–S17). Such reduced aromatic character correlates with localized spin density in **4**, consistent with a progressive localization of the α‐ and β ‐SOMOs to opposite sides of the molecule.

Anisotropy of the induced current density (ACID) calculations performed on the singlet ground states were employed to better visualize the delocalization of *π*‐electrons [57]. Compounds **1–4** all display diatropic ring currents along the periphery of their molecular backbones, while prominent paratropic ring currents on ring 2 of the acceptor and within the donor cores indicate significant quinoidal character, consistent with bond length analysis (Figure 8C; Figure S46). The singlet ground states show progressively increased double‐bond character across the central rings, which progressively restricts bond rotation between the donor and acceptor, with donor‐acceptor dihedral angles (*θ*) that decrease from 24.2° in **1** to <1° in **4**. (Figure S45). The increased planarity from **1** → **4** correlates with a concomitant decrease in their energy gaps and progressively stronger spin delocalization across the series, which can be attributed to the enhanced MO hybridization between the electron‐rich donors and the strong, pro‐quinoidal acceptor. Such strong orbital mixing between donor and acceptor units stabilizes quinoidal bonding patterns, reduces SOMO−SOMO spatial overlap, and favors progressively localized spin densities to opposing sides of the molecules [22, 27]. The optimized triplet structures display nearly planar geometries with *θ *that decrease from 2.05° in **1** to 0.02° in **4** (Figure S44), which result from significantly smaller interannular bond lengths 1.423 and 1.416 Å (Figure S47). The increased planarity and quinoidal character is consistent with the delocalization of triplet spin density across the donor‐acceptor interannular bond (Figures S38–S41), which results in large hyperfine couplings to both ^14^N nuclei on the acceptor and ^1^H nuclei on the donors observed by EPR.

## 3 Conclusion

We demonstrate that strong donor‐acceptor interactions provide a viable framework for inducing open‐shell character in small, well‐defined molecular systems. The fusion of progressively *π*‐extended electron‐rich donor units to a strong 6,7,8,9‐tetrachloro‐[1,2,5]thiadiazolo[3,4‐b]phenazine acceptor results in a systematic narrowing of the energy gaps, ∆*E*_ST_, and increase in *y*, which correlates with spatial distribution of α‐ and β‐SOMOs within the *π*‐conjugated backbones. This spin polarization yields unique long‐range hyperfine interactions rarely observed in organic diradicaloids, which depend strongly on the structure of the donor, thereby providing the capability for fine‐tuning the electronic and spin properties of this family of diradicaloids. Moreover, strong electronic correlations give rise to spin–spin, magnetic, and transport functions such as high electrical conductivity that are otherwise difficult to achieve. The well‐defined and compact nature of these materials systems enabled high‐fidelity theoretical descriptions of the multiconfigurational nature of these materials using MRSF‐TDDFT. Thus, these materials provide advantages over other open‐shell materials for establishing connections between chemical, topological, spin, and magnetic structures with physicochemical properties and (opto)electronic functionality. These include: (1) the facile preparation of pro‐quinoidal materials with narrow energy gaps and a progressive increase in spin delocalization, (2) high chemical stability, (3) neutral open‐shell ground states, (4) solubility that enables processing into thin films, and (5) tunable spin–spin coupling and electronic communication via intramolecular through‐bond and intermolecular *π*‐*π* interactions. These collective attributes are difficult to achieve from other materials systems.

## Author Contributions

The manuscript was written through the contributions of all authors. All authors have given approval to the final version of the manuscript.

## Conflicts of Interest

The authors declare no conflicts of interest.

## Supporting information




**Supporting File**: smll72779‐sup‐0001‐SuppMat.docx.

## Data Availability

The data that support the findings of this study are available in the supplementary material of this article.
