# Supplementary material for: Modular Donor‐Acceptor Diradicaloids Based on an Electron Deficient N‐Heteroacene Acceptor
Source: Small. 2026 Feb 10;22(20):e10228. doi: 10.1002/smll.202510228 (PMC13054449; doi:10.1002/smll.202510228)
Supplement: Supplementary file 1 — Supporting File: smll72779‐sup‐0001‐SuppMat.docx. [file SMLL-22-e10228-s001.docx]

Supplemental Information

**Modular Donor-Acceptor Diradicaloids Based on an Electron Deficient *N*-Heteroacene Acceptor**

Tanner L. Smith, Zhendian Zhang, Tanya A. Balandin, Paramasivam Mahalingam, Andrew H. Comstock, Anna M. Österholm, Michael K. Bowman, Molly M. Lockart, Guoxiang Hu, and Jason D. Azoulay*

**1. General Remarks**

**2. Synthesis**

**3. Experimental Procedures**

3.1. UV-Vis-NIR and Fourier Transform Infrared Spectroscopy

3.2. Electrochemistry

3.3 Thermogravimetric Analysis

3.4. Device Fabrication and Charge Transport Measurements

3.5. Electron Paramagnetic Resonance Spectroscopy

3.6. SQUID Magnetometry

3.7. Quantum Chemical Calculations

**4. Supplemental Figures S1–S51**

**5. Supplemental Tables S1–S29**

**6. Supplemental References**

**1. General Remarks**

All manipulations of air/and or moisture sensitive compounds were performed under an inert atmosphere using standard glove box and Schlenk techniques. Reagents, unless otherwise specified, were purchased from Sigma-Aldrich or VWR and used without further purification. Chloroform, xylenes, chlorobenzene, and dichloromethane were degassed and dried over 4 Å molecular sieves prior to use. Deuterated solvents (1,1,2,2-tetrachloroethane-*d*_2_ and chloroform-*d)* were purchased from Cambridge Isotope Laboratories and used as received. [1,1'‑Bis(diphenylphosphino)ferrocene]palladium(II) dichloride was purchased from TCI and used as received. (5-Hexyl-2-thienyl)trimethylstannane and (6-undecylthieno[3,2-*b*]thien-2-yl)tributylstannane, were purchased from Derthon and used as received. (5′-Hexyl[2,2′-bithiophen]-5-yl)trimethylstannane,^[1]^ (4,4-dioctyl-4*H*-cyclopenta[2,1-*b*:3,4-*b*′]dithien-2-yl)trimethylstannane^[2]^, and 4,11-dibromo-6,7,8,9-tetrachloro-[1,2,5]thiadiazolo[3,4-b]phenazine^[3]^ were prepared according to previously reported procedures. ^1^H and ^13^C NMR spectra were collected on a Bruker AV3-HD 500 MHz or AV 400 MHz spectrometer and chemical shifts, δ (ppm) were referenced to the residual solvent impurity peak of the solvent. Data are reported as: s = singlet, d = doublet, t = triplet, m = multiplet, and coupling constant, (*J*) are reported in Hz. Flash chromatography was performed on a Teledyne Isco CombiFlash Purification System using RediSep Rf prepacked columns.

**2. Synthesis**

**6,7,8,9-tetrachloro-4,11-bis(5-hexylthiophen-2-yl)-[1,2,5]thiadiazolo[3,4-b]phenazine (1)** An oven-dried pressure-safe vial with stir bar was loaded with 4,11-dibromo-6,7,8,9-tetrachloro-[1,2,5]thiadiazolo-[3,4-b]phenazine (75.0 mg, 0.140 mmol) and brought inside a nitrogen-filled glovebox. (5-hexylthiophen-2-yl)trimethylstannane (2.5 eq., 116 mg, 0.351 mmol), Pd(dppf)Cl_2_ (6.0 mol%, 6.88 mg, 8.43 µmol), and xylenes (1.0 ml) were then added to the vial, which was then sealed inside the glovebox and then stirred at 80 ^o^C overnight. The vial was then allowed to cool to room temperature, and the mixture was precipitated into methanol and collected via filtration. The crude product was purified by flash column chromatography in hexanes:DCM (6:1) to give 62.2 mg (63%) of the product as a dark solid. ^1^H NMR (400 MHz, [D]chloroform, 298 K): *δ* 8.10 (d, ^3^*J*(H,H) = 4.0 Hz, 2H; CH), 6.45 (d, ^3^*J*(H,H) = 4.0 Hz, 2H; CH), 2.66 (t, ^3^*J*(H,H) *=* 4.0 Hz, 4H; CH_2_), 1.65 (q, ^3^*J*(H,H) *=* 4.0 Hz, 4H; CH_2_), 1.40 (m, 12H; CH_2_), 0.96 (t, ^3^*J*(H,H) *=* 4.0 Hz, 6H; CH_3_). ^13^C NMR (126 MHz, chloroform-*d*): *δ* 154.00, 149.53, 136.29, 134.67, 133.82, 133.44, 133.25, 131.58, 31.73, 30.76, 30.11, 29.20, 22.74, 14.23. UV–vis (chloroform): *λ*_max_(*ε*) = 918 (10,363). Mass spectrometry (MS) [electrospray ionization (ESI)] exact mass calculated for C_32_H_30_Cl_4_N_4_S_3_ is as follows: m/z 706.0392 [M-] and 706.0398 (found).

**6,7,8,9-tetrachloro-4,11-bis(5-undecylthieno[3,2-b]thiophen-2-yl)-[1,2,5]thiadiazolo[3,4-b]phenazine (2)** An oven-dried pressure-safe vial with stir bar was loaded with 4,11-dibromo-6,7,8,9-tetrachloro-[1,2,5]thiadiazolo-[3,4-b]phenazine (100.0 mg, 0.171 mmol) and brought inside a nitrogen-filled glovebox. Tributyl(5-undecylthieno[3,2-b]thiophen-2-yl)stannane (2.5 eq., 229.0 mg, 0.428 mmol), Pd(dppf)Cl_2_ (6.0 mol%, 8.40 mg, 10.3 µmol), and xylenes (1.4 ml) were then added to the vial, which was then sealed inside the glovebox and stirred at 80 ^o^C overnight. The vial was then allowed to cool to room temperature, and the mixture was precipitated into methanol and collected via filtration. The crude product was purified by column chromatography in hexanes:DCM (4:1) to give 88.3 mg (54%) of the product as a dark solid. ^1^H NMR (400 MHz, [D_2_]1,1,2,2-tetrachloroethane, 298 K): *δ* 8.15 (s, 2H; CH), 6.80 (s, 2H; CH), 2.26 (m, 4H; CH_2_), 1.51 (m, 4H; CH_2_), 1.33 (m, 16H; CH_2_), 0.96 (t, ^3^*J*(H,H) = 6.0 Hz, 6H; CH_3_). ^13^C NMR (126 MHz, chloroform-*d*): *δ* 149.24, 147.50, 138.76, 137.58, 135.29, 134.12, 133.46, 130.61, 126.10, 124.60, 118.85. UV–vis (chloroform): *λ*_max_(*ε*) = 997 (12,797). Mass spectrometry (MS) [electrospray ionization (ESI)] exact mass calculated for C_32_H_30_Cl_4_N_4_S_3_ is as follows: m/z 958.1399 [M-] and 958.1397 (found).

**6,7,8,9-tetrachloro-4,11-bis(5'-octyl-[2,2'-bithiophen]-5-yl)-[1,2,5]thiadiazolo[3,4-b]phenazinephenazine (3)** An oven-dried pressure-safe vial with stir bar was loaded with 4,11-dibromo-6,7,8,9-tetrachloro-[1,2,5]thiadiazolo-[3,4-b]phenazine (75.0 mg, 0.140 mmol) and brought inside a nitrogen-filled glovebox. Trimethyl(5'-octyl-[2,2'-bithiophen]-5-yl)stannane (2.5 eq., 155 mg, 0.351 mmol), Pd(dppf)Cl_2_ (6.0 mol%, 6.88 mg, 8.43 µmol), and xylenes (1.0 ml) were then added to the vial, which was then sealed inside the glovebox and heated at 80^o^C with stirring for 16 hours. The vial was then allowed to cool to room temperature, and the mixture was precipitated in methanol and collected via filtration. The crude product was purified by column chromatography in hexanes:DCM (2:1) to give the product as a brown solid (0.103 mmol, 73%). ^1^H NMR (400 MHz, [D_2_]1,1,2,2-tetrachloroethane, 298 K): *δ* 7.88 (m, 2H; CH), 6.75 (m, 2H; CH), 6.63 (m, 2H; CH), 6.52 (m, 2H; CH), 2.79 (m, 4H; CH_2_), 1.73 (m, 4H; CH_2_), 1.39 (m, 20H; CH_2_), 0.98 (t, ^3^*J*(H,H) = 8.0 Hz, 6H; CH_3_). UV–vis (chloroform): *λ*_max_(*ε*) = 1098 (13,261). Mass spectrometry (MS) [electrospray ionization (ESI)] exact mass calculated for C_32_H_30_Cl_4_N_4_S_3_ is as follows: m/z 926.0773 [M-] and 926.0780 (found).

**6,7,8,9-tetrachloro-4,11-bis(4,4-dioctyl-4H-cyclopenta[2,1-b:3,4-b']dithiophen-2-yl)-[1,2,5]thiadiazolo[3,4-b]phenazine (4)** An oven-dried pressure-safe vial with stir bar was loaded with 4,11-dibromo-6,7,8,9-tetrachloro-[1,2,5]thiadiazolo-[3,4-b]phenazine (75.0 mg, 0.140 mmol) and brought inside a nitrogen-filled glovebox. (4,4-dioctyl-4H-cyclopenta[2,1-b:3,4-b']dithiophen-2-yl)trimethylstannane (2.5 eq., 199 mg, 0.351 mmol), Pd(dppf)Cl_2_ (6.0 mol%, 6.88 mg, 8.43 µmol), and xylenes (1.0 ml) were then added to the vial, which was sealed inside the glovebox and heated at 80^o^C with stirring for 16 hours. The vial was then allowed to cool to room temperature, and the mixture was precipitated into methanol and collected via filtration. The crude product was purified by column chromatography in hexanes:DCM (4:1) to give 53.6 mg of the product as a dark solid (45.5 µmol, 33%). ^1^H NMR (400 MHz, [D_2_]1,1,2,2-tetrachloroethane*,* 273 K): *δ* 9.25 (s, 2H; CH), 7.42 (m, 2H; CH), 7.11 (d, ^3^*J*(H,H) = 4.0 Hz, 2H; CH), 2.03 (m, 8H; CH_2_), 1.22 (m, 48H; CH_2_), 0.82 (m, 12H; CH_3_). ^13^C NMR (126 MHz, chloroform-*d*): *δ* 160.79, 159.70, 151.11, 139.66, 137.75, 136.81, 134.59, 131.72, 128.86, 127.63, 122.01. UV–vis (chloroform): *λ*_max_(*ε*) = 1263 (19,219). Mass spectrometry (MS) [electrospray ionization (ESI)] exact mass calculated for C_32_H_30_Cl_4_N_4_S_3_ is as follows: m/z 1174.3277 [M-] and 1174.3295 (found).

**3. Experimental section**

**3.1 UV–vis–NIR and Fourier transform infrared spectroscopy.** UV–vis–NIR spectra were recorded from 200 to 2000 nm using an Agilent Cary 5000 UV–vis–NIR spectrophotometer. Solution measurements were performed in chloroform (~10^-5^ м) in 1 mm and 1 cm path length quartz cuvettes. Thin films were prepared by spin coating a chloroform or chlorobenzene solution (10 mg mL^-1^) onto quartz substrates at 1000 rpm. Temperature-dependent UV–vis spectra were collected using a Peltier attachment model TC225 from Quantum Northwest and 1 cm path length quartz cuvettes.

**3.2 Electrochemistry.** Electrochemical properties were determined by cyclic voltammetry (3 cycles, 25 mV s^-1^) carried out in room temperature in degassed anhydrous dichloromethane (0.1 µм) with tetrabutylammonium hexafluorophosphate (0.1 м) as the supporting electrolyte. The cyclic voltammograms were recorded using an EG&G PAR 273A potentiostat/galvanostat under CorrWare control. The oxidation behavior was probed between −0.42 and 1.08 V vs Fc/Fc^+^ whereas electrochemical reduction was probed between -0.42 V and -1.92 V vs. Fc/Fc^+^. The working electrode was glassy carbon disc electrode (0.07 cm^2^), the counter electrode was a coiled platinum wire and the pseudoreference electrode was Ag/AgCl wire. After each measurement the reference electrode was calibrated against the against the Fc/Fc^+^ redox couple (Fc/Fc^+^ = 0.42 V vs. Ag/Ag^+^). The electrochemical band gap for each compound was calculated from the difference between the E_1/2_ of the first oxidation peak and the E_1/2_ of the first reduction peak. All electrochemical measurements were performed under an argon atmosphere.

**3.3 Thermogravimetric analysis**. Thermogravimetric (TGA) analysis of compounds **1-4** were performed on a Mettler-Toledo TGA2 by ramping the temperature from 25 to 800 °C at a rate of 20 °C/min under nitrogen atmosphere.

**3.4 Device Fabrication and Charge Transport Measurements.** The conductivity and organic field-effect transistor (OFET) characteristics were evaluated using a typical bottom-gate, bottom-contact geometry. To prepare the Si/SiO_2_ substrates (2.0 cm × 1.5 cm), the substrates are first cut and cleaned thoroughly. They are washed with a 2% Hellmanex detergent solution in DI water, followed by DI water, acetone, and 2-isopropanol, each with 10 minutes of sonication. The cleaned substrates are dried using nitrogen with a filtered nozzle and baked in an oven for at least one hour. Afterward, the substrates undergo UV/ozone cleaning for 20 minutes before being immediately transferred into a glovebox. Inside the glovebox, gold (Au) electrodes (60 nm thick, deposited at a rate of 1 Ǻ s^‒1^) are deposited on a chromium adhesive layer (5 nm thick, rate: 0.1 Ǻ s^‒1^) using a shadow mask (*L* = 30, 40, 50, 60, 80 µm, *W* = 1000 µm). The heavily n-doped silicon substrates with a 300 nm thermally grown SiO_2_ dielectric were prepared as the bottom-gate electrode. Subsequently, 3 mм octadecyl trichlorosilane (CH_3_(CH_2_)_17_SiCl_3_ (OTS)) in trichloroethylene (TCE) is applied to the pre-fabricated substrates by spin-coating at 3000 rpm for 10 seconds, after allowing the solution to sit for 15 seconds. The substrates are dried in the glovebox for 15 minutes, washed twice with hexane, and dried for 2 hours before being removed from the glovebox. The OTS-treated substrates are then exposed overnight to ammonium hydroxide (28-30% in water) in a vacuum desiccator to promote hydrolysis. Any residual OTS is removed by ultrasonicating in toluene for 2 minutes, followed by rinsing with hexane and acetone. The substrates are dried with nitrogen before being transferred back into the glovebox, where 10 mg mL^-1^ solutions of the molecules were spin cast using 1000 rpm for 60 s onto the substrates with pre-patterned Au electrodes. The molecules are soluble in common organic solvents and could be readily processed onto glass, silicon, or plastic substrates.

Devices were tested on a probe station (Signatone 1160 series) inside a nitrogen-filled glovebox, and the data were recorded on a Keysight B1500A semiconductor characterization system. The mobility was extracted from the linear region of the transfer curve in a transistor geometry based on Eq. S1:

|  | $\text{μ}\text{ =}\frac{\text{L}}{\text{W}\text{C}_{\text{i}}\text{V}_{\text{D}}}\text{ }\frac{\text{∆}\text{I}_{\text{D}}}{\text{∆}\text{V}_{\text{G}}}$ | (Eq. S1) |
| --- | --- | --- |

where *µ*, *L*, *W*, *C*_i_, *V*_D_, *I*_D_, *and V*_G_ represent the mobility, channel length, channel width, capacitance of the dielectric layer (300 nm thick SiO_2_ layer), drain voltage, drain current, and gate voltage, respectively.^[4]^

**3.5 Electron Paramagnetic Resonance Spectroscopy** Room temperature continuous-wave (CW) EPR spectra were recorded on a Bruker ELEXSYS-II E500 CW EPR spectrometer operating in the X-band with a CW ELEXSYS Super High Sensitivity Probehead resonator using the following instrumental parameters: 0.20 mW microwave power, 30 dB attenuation, 25 scans, 20.48 s sweep time, and a 3321-3381 G magnetic field sweep. Variable temperature (VT) measurements were performed using a Bruker N2 VTU Controller. Samples were loaded into 4 mm high-purity quartz tubes. The *g*-factors were calculated experimentally and confirmed by fitting the acquired CW spectra with a MATLAB EasySpin simulation for a triplet S=1 system with a Voigt line shape.^[5]^

VT EPR measurements of **1-4** were performed on dilute ~10^-5^ м solutions in 1-chloronaphthalene (CN), toluene, or bromobenzene to characterize the single-molecule behavior and directly measure the singlet-triplet energy splitting (Δ*E*_ST_). By integrating the CW signal collected over a range of 300-375 K and plotting it versus 1/*T*, Δ*E*_ST_ can be extracted by fitting to the Bleaney-Bowers equation (Eq. S2):

|  | $\text{I}_{\text{EPR}}\text{=}\frac{\text{C}}{\text{T}}\frac{\text{3}\text{e}^{\text{-2}\text{J/}\text{k}_{\text{B}}\text{T}}}{\text{1}\text{+}\text{3}\text{e}^{\text{-2}\text{J/}\text{k}_{\text{B}}\text{T}}}$ | (Eq. S2) |
| --- | --- | --- |

where *C* is a constant, *k*_B_ is the Boltzmann constant, *J* is the intramolecular exchange coupling constant, and 2*J* is Δ*E*_ST._ These results reveal that the signal intensity increases as temperature increases, consistent with a thermally populated triplet state.

CW EPR spectra were simulated in MATLAB using the Easyspin toolbox (version 6.0.6).^[5]^ Simulations of **1** and **2** in CN were performed using the ‘pepper’ function, which supports *S* = 1 species. Zero-field splitting parameters *D* and *E* were set to zero to approximate rapid isotropic tumbling in solution. The EPR spectral simulations improved dramatically with inclusion of a second minor species. The minor species had similar couplings, slightly shifted g-values and broader linewidths – behavior suggesting formation of small aggregates in solution that dissociated at higher temperatures. Hyperfine couplings to two unique^14^N nuclei and two unique ^1^H nuclei were resolved for **1**, while couplings to two ^14^N nuclei and one ^1^H nucleus were resolved for **2**. **Tables S7** and **S8** summarize all simulation parameters.

**3.6 Quantum Interference Device (SQUID) Magnetometry.** Magnetometry data were collected using the Quantum Design MPMS3 SQUID-VSM. For the magnetic susceptibility as a function of temperature measurements, the magnetic moment was recorded by SQUID-VSM upon warming over the range 2–400 K after cooling in a zero magnetic field and allowing the sample to reach thermal equilibrium (10 minutes) at 2 K. The background signal of the sample holder and an empty VSM capsule of the same length as the one filled with the polymer sample was recorded using the same measurement sequence and subtracted from the original signal. The mass magnetic susceptibility χ was determined from the magnetic moment by the equation, where the magnetization *M* is defined as the magnetic moment per unit mass and *H* is the applied magnetic field (Eq. S4).^[6]^

|  | $\text{χ}\text{ = }\frac{\text{M}}{\text{H}}$ | (Eq. S4) |
| --- | --- | --- |

The modified Curie-Weiss Law (Eq. S5) for paramagnets where *C* is the material-dependent Curie constant, *T* is temperature, *θ* is the Weiss constant, and *X_0_* is for any observable diamagnetic offset in magnetic susceptibility, fits to the *χ* versus temperature data.^[7]^

|  | $\text{χ}\text{ = }\frac{\text{C}}{T-\theta}+\text{χ}_{\text{0}}$ | (Eq. S5) |
| --- | --- | --- |

**3.7 Quantum Chemical Calculations**. In recent years, computational tools have been utilized to describe the open-shell character of molecular and polymeric systems. The contributions of strong *π-*correlations between unpaired electrons to the ground state electronic structure can be described in terms of the singlet-triplet energy gap (Δ*E*_ST_), diradical character index (*y*), spatial overlap of electron spins, spin density distribution between the α- and β-frontier molecular orbitals (FMOs), nucleus independent chemical shift (NICS(1)iso), electrostatic potential (ESP) surface, anisotropy of the induced current density (ACID), and bond length alternation (BLA). Mixed-Reference Spin-Flip Time-Dependent Density Functional Theory (MRSF-TDDFT)^[8]^ is a computational method designed to accurately describe excited states in systems with strong static correlation, such as open-shell singlet donor–acceptor (D–A) conjugated molecules. Building on the spin-flip TDDFT framework,^[9]^ which accesses low-spin states by flipping the spin of an electron relative to a high-spin reference, MRSF-TDDFT improves reliability by using a mixed-reference state composed of a combination of multiple high-spin determinants. This approach reduces spin contamination and captures near-degeneracy effects that are common in diradicaloid systems. As a result, MRSF-TDDFT provides a balanced treatment of ground and excited states, making it particularly well suited for modeling the electronic structure and excitation behavior of D–A systems with open-shell singlet ground states.

Energy-minimized geometries were obtained using broken symmetry calculations with the CAM-B3LYP functional and Def2-TZVP basis set with Gaussian package.^[10]^ The broken symmetry first optimizes the triplet state geometry. A wave function stability test was conducted to the optimal triplet state geometry. After an additional geometry optimization, the wave function was enforced to the singlet state by flipping the wave function of one side of the molecule to produce the broken symmetry solution. All energy-minimized geometries obtained from broken symmetry calculations were subjected to excited state calculations using MRSF-TDDFT with (U)CAM-B3LYP/def2-TZVP.^[11-13]^ MRSF-TDDFT calculations were performed using the GAMESS software package with Restricted Open-Shell Kohn-Sham (ROKS) reference states.^[14]^ The alkyl side chains of the molecules were truncated to methyl (–CH_3_) groups to reduce computational cost. The CAM-B3LYP functional was tuned with $\mu$ of 0.05 by comparing the MRSF-TDDFT excitation energy with experimental data.

Dyson orbital analysis^[15]^ from GAMESS was employed to investigate the nature of excitation transitions. Dyson orbital analysis provides a quantitative description of electron detachment or attachment by evaluating the overlap between N-electron and (N±1)-electron wavefunctions.^[16]^ Unlike canonical molecular orbitals, which are eigenfunctions of a mean-field operator, Dyson orbitals directly represent the one-electron amplitude for ionization or electron capture, incorporating electron correlation and orbital relaxation effects. For ionization processes, the Dyson orbital is defined as the projection of the neutral state's wavefunction onto the ionized state's wavefunction with one electron removed. The spatial distribution and associated norm of excited states in Dyson orbitals provide insight into the character and strength of the ionization channel and excitation transitions.^[15]^

The diradical character index (*y*) at the same level of theory was evaluated from HONO and LUNO occupancies by following Yamaguchi’s formula (Eq. S6) ^[17, 18]^

|  | $\text{y }\text{= 1}-\frac{\text{2}\text{T}}{\text{1}-\text{T}^{\text{ 2}}}$ | (Eq. S6) |
| --- | --- | --- |

where *T* is defined as the orbital overlap that can be calculated using the occupation numbers (*n*) obtained from the unrestricted natural orbitals in equation (Eq. S7):

|  | $T=\frac{n_{HONO}-n_{LUNO}}{2}$ | (Eq. S7) |
| --- | --- | --- |

Spin locations were predicted from the natural spin densities of Kohn-Sham molecular orbitals (MO). The molecular electrostatic potential surface (MESP) and FMOs involved in electron and spin density distribution analyses were calculated using the CAM-B3LYP functional and def2-TZVP basis set. NICS*_iso(1)_* calculations were performed using the gauge-independent atomic orbital (GIAO) method to assess the effect of the ring current produced by the *π*-electrons from each ring of the model oligomers. To diminish contributions of *σ-*bonding contributions to the *π*-ring current, a so-called ghost atom (*B_q_*) was placed 1 Å perpendicular to the ring plane. The obtained values have been generally reported as the negative value of the absolute isotropic magnetic shielding, where large negative NICS values indicate a more pronounced aromaticity containing (4*n*+2) *π-*electrons, while the smaller negative values suggest an involvement of quinoidal characteristics with 4*nπ* electrons. ACID calculations were employed to evaluate the flow of ring current density over the *π-*framework in terms of global and local aromaticity induced by the donor and acceptor units.

**4. Supplemental Figures**


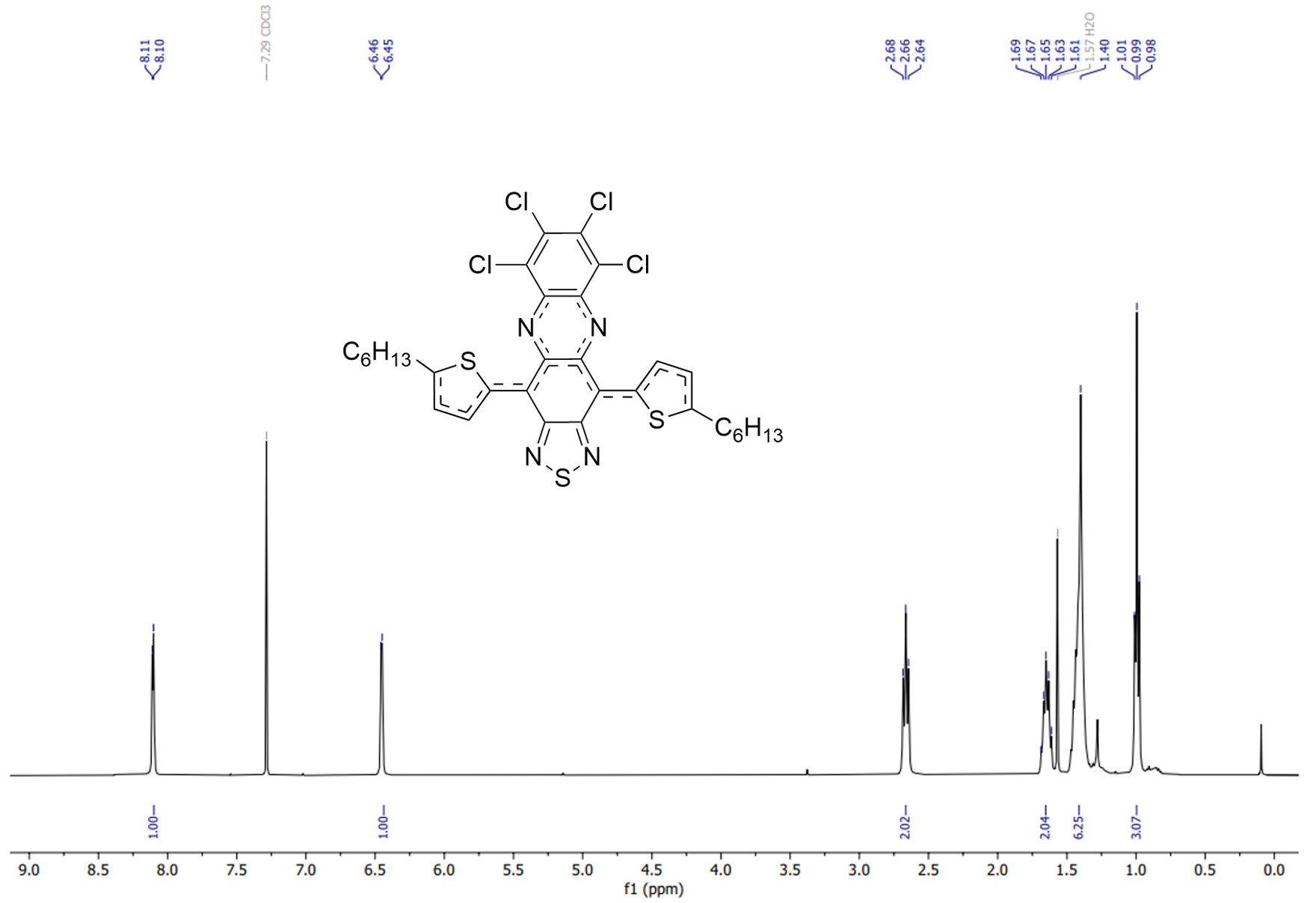


**Figure S1.** ^1^H NMR spectrum (400 MHz, chloroform-d) of **1** at 298 K.

**
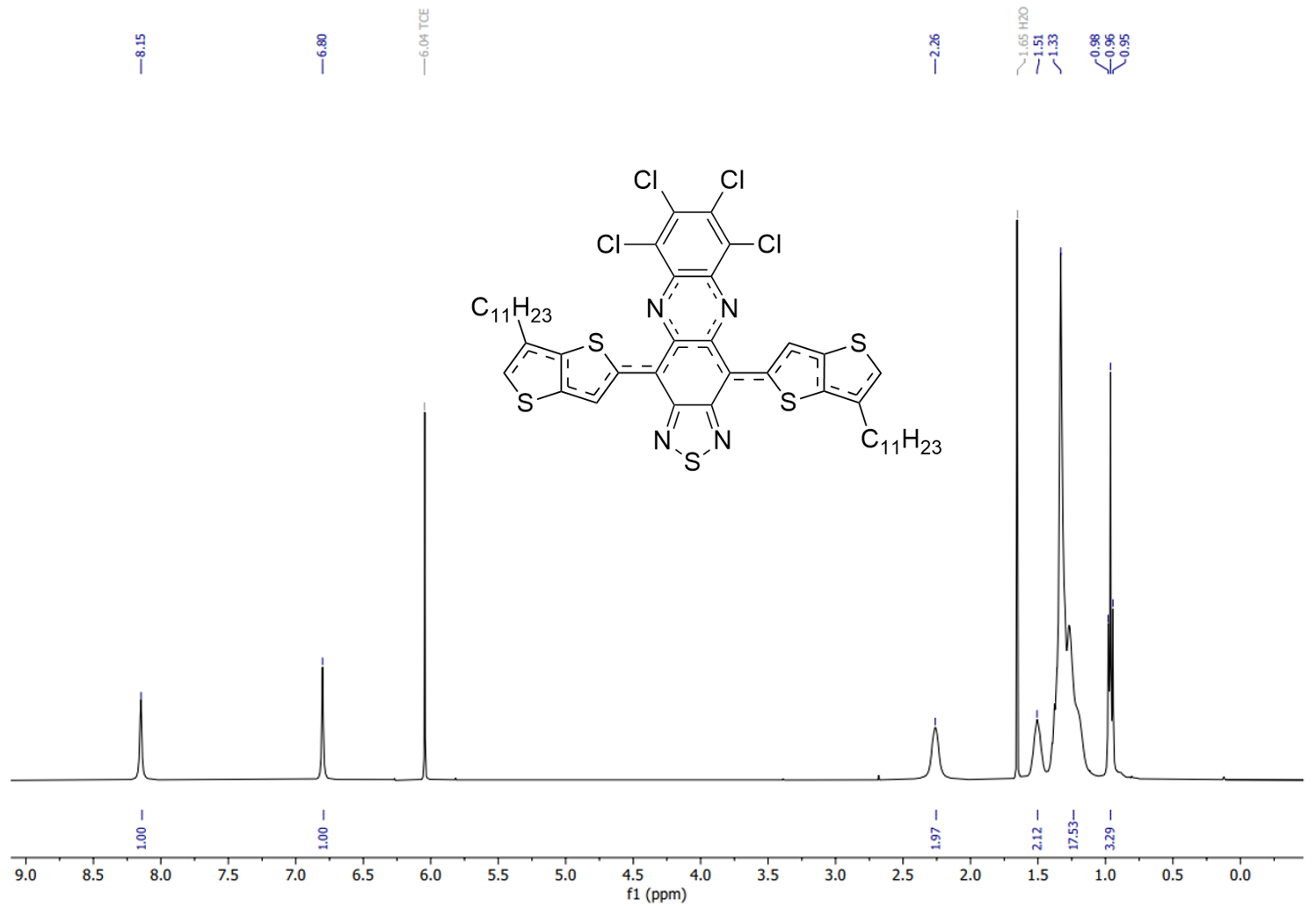
**

**Figure S2.** ^1^H NMR spectrum (400 MHz, 1,1,2,2-tetrachloroethane-d_2_) of **2** at 298 K.


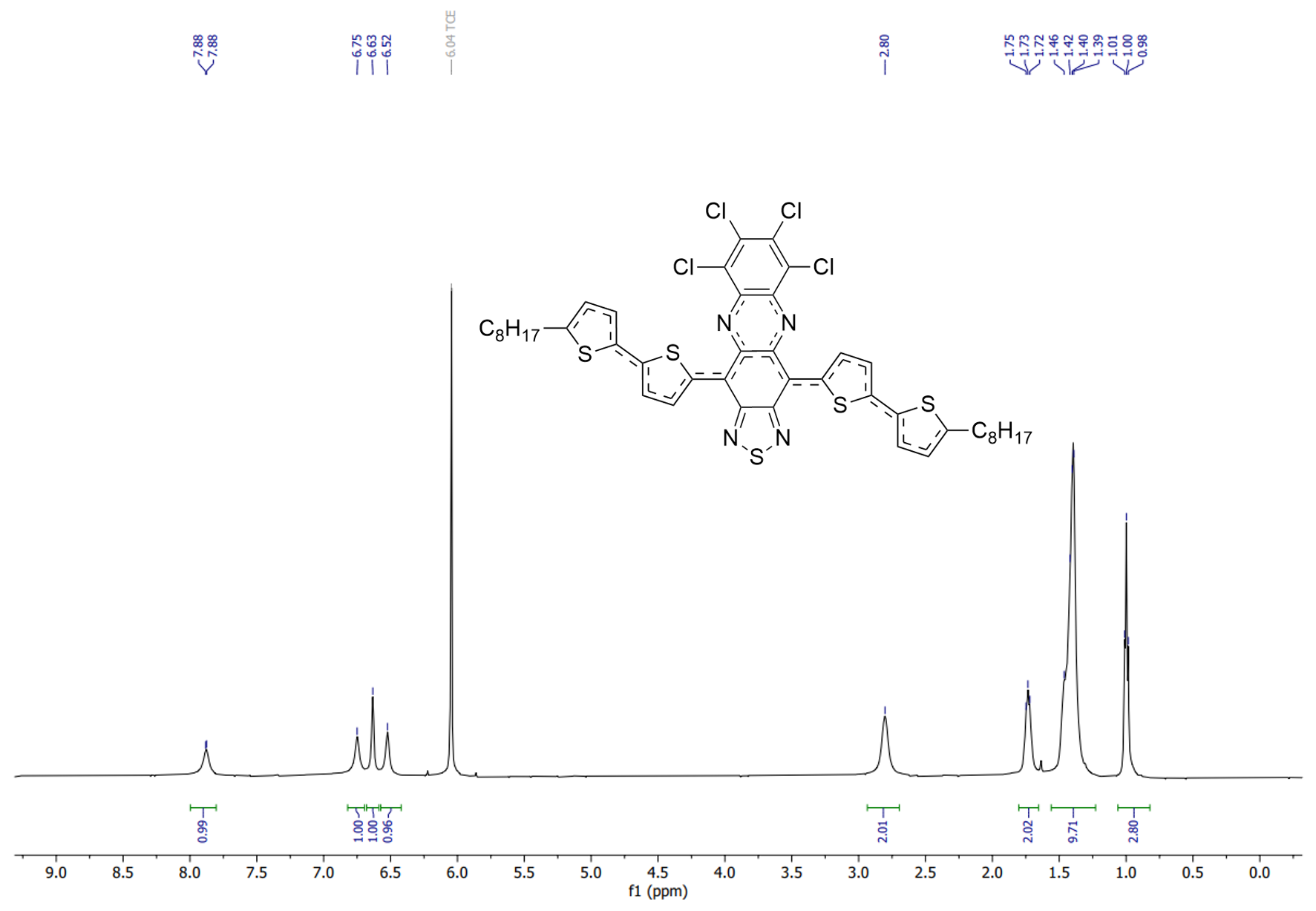


**Figure S3.** ^1^H NMR spectrum (400 MHz, 1,1,2,2-tetrachloroethane-d_2_) of **3** at 298 K.


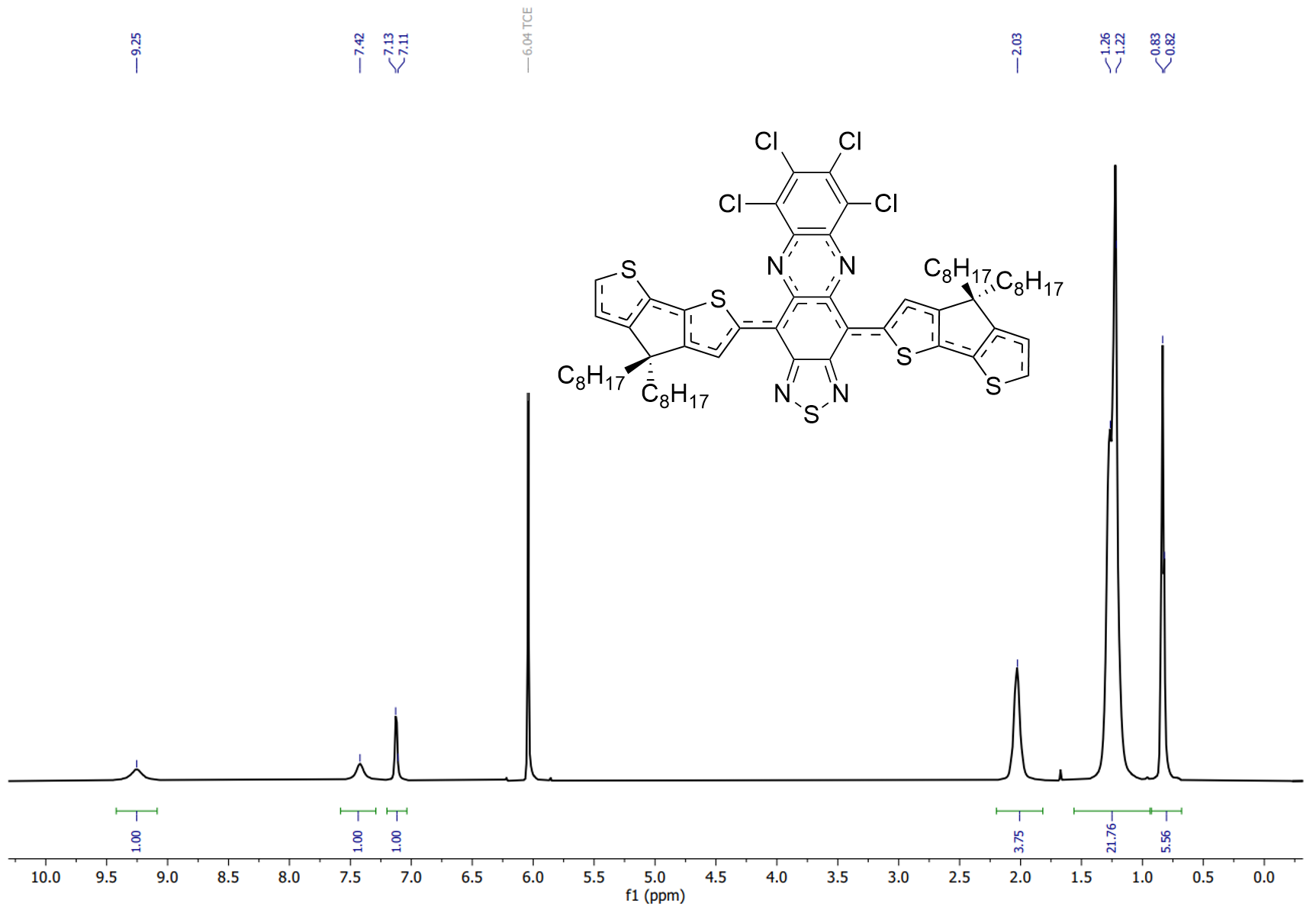


**Figure S4.** ^1^H NMR spectrum (400 MHz, 1,1,2,2-tetrachloroethane-*d_2_*) of **4** at 273 K.


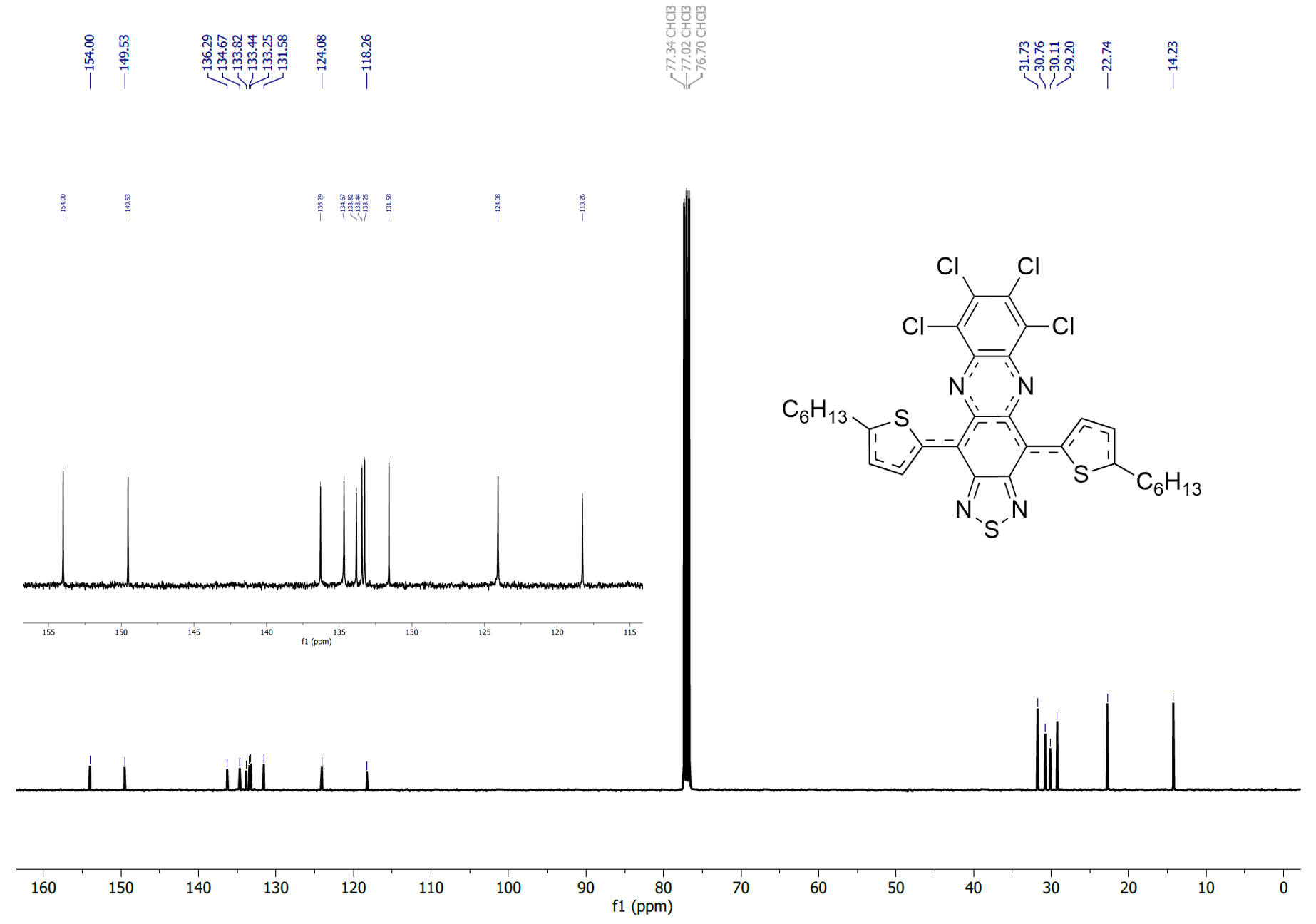


**Figure S5.** ^13^C NMR spectrum (500 MHz, chloroform-*d*) of **1** at 298 K.


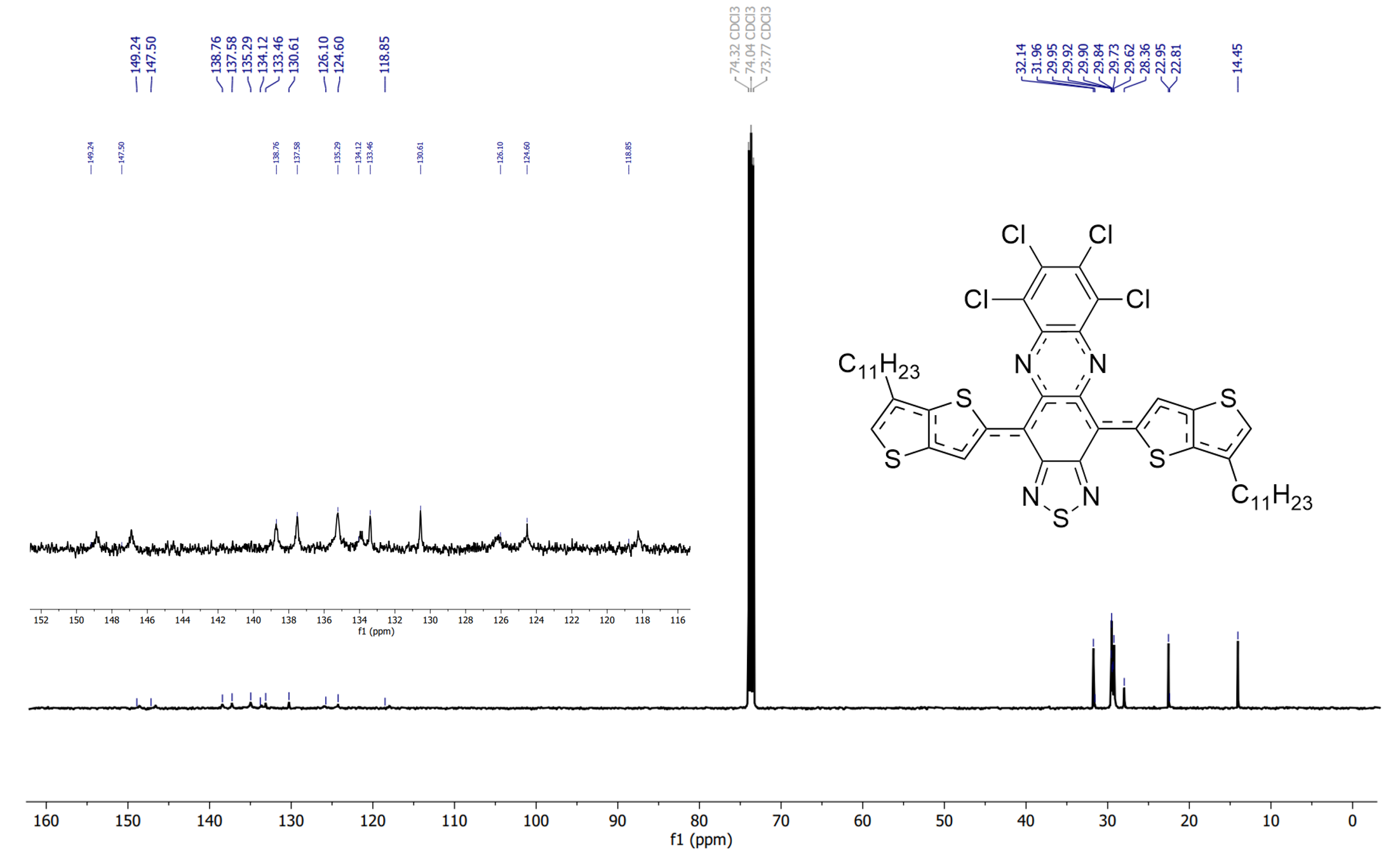


**Figure S6.** ^13^C NMR spectrum (500 MHz, chloroform-*d*) of **2** at 298 K.


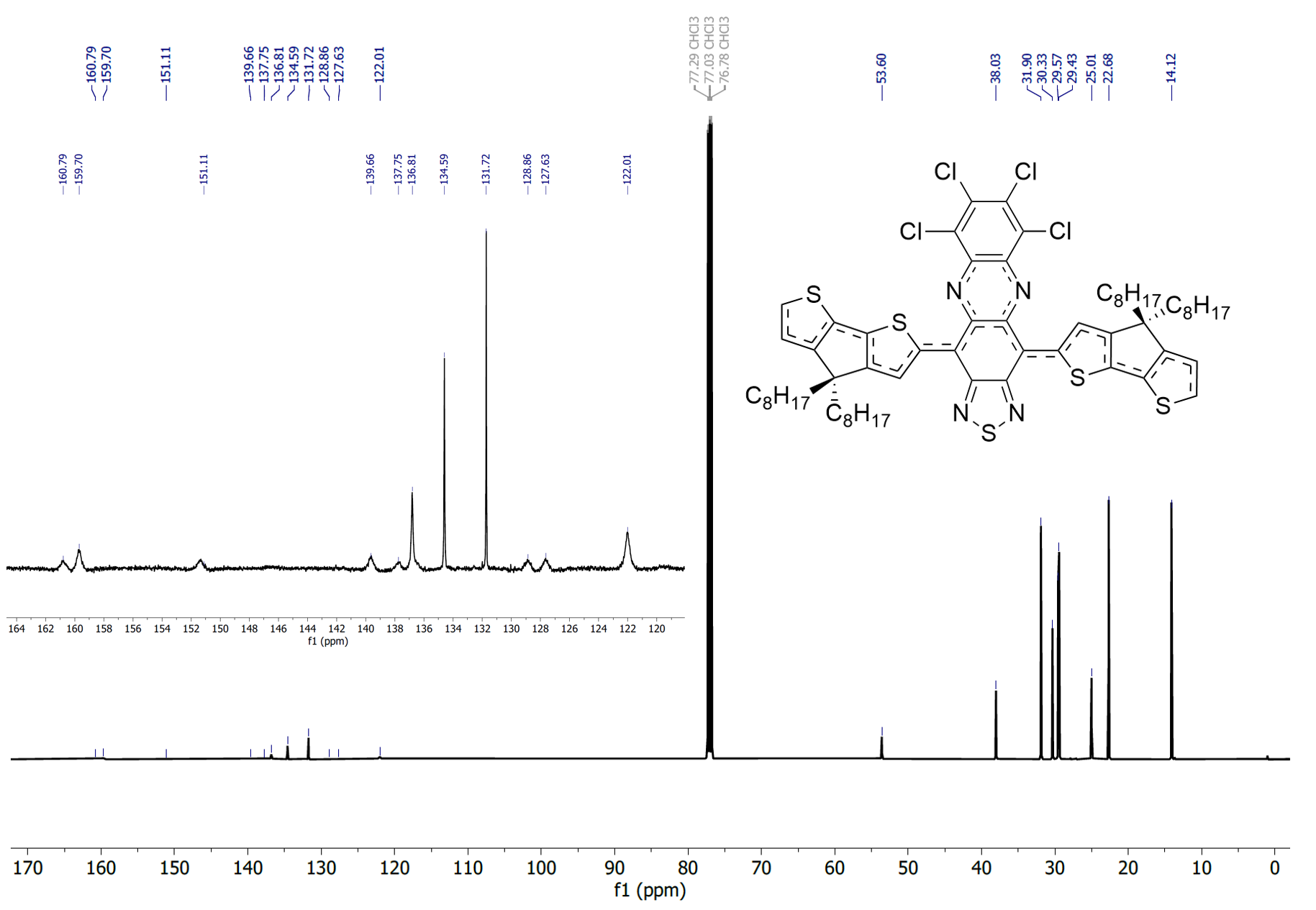


**Figure S7.** ^13^C NMR spectrum (500 MHz, chloroform-*d*) of **4** at 298 K.


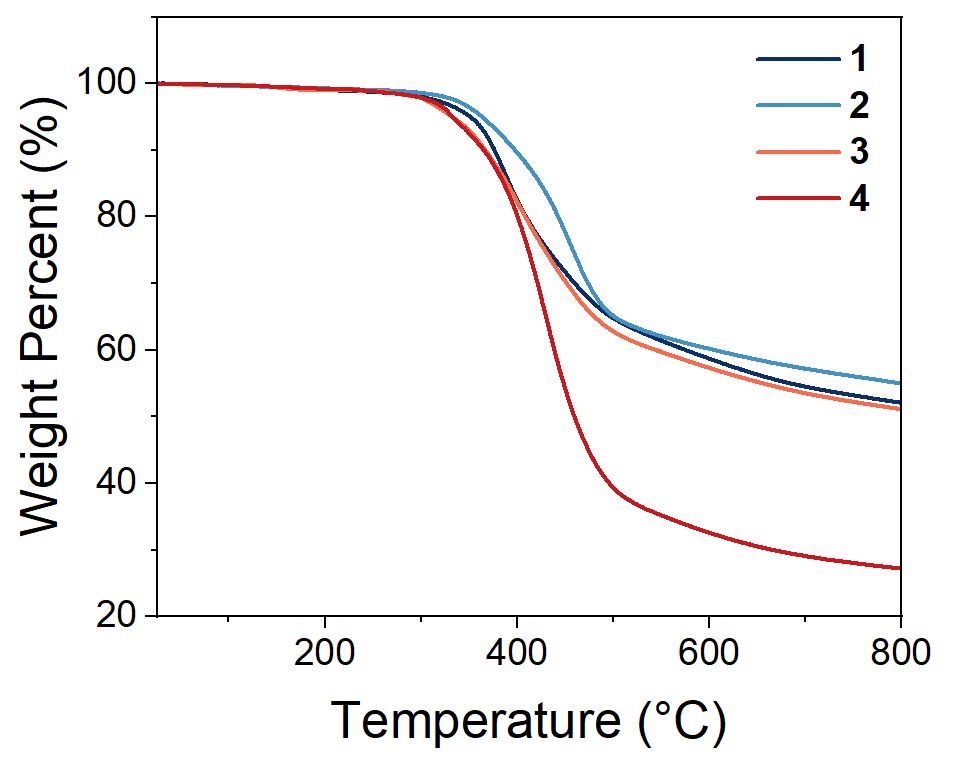


**Figure S8.** TGA curves of compounds **1-4** measured from 25-800 °C.


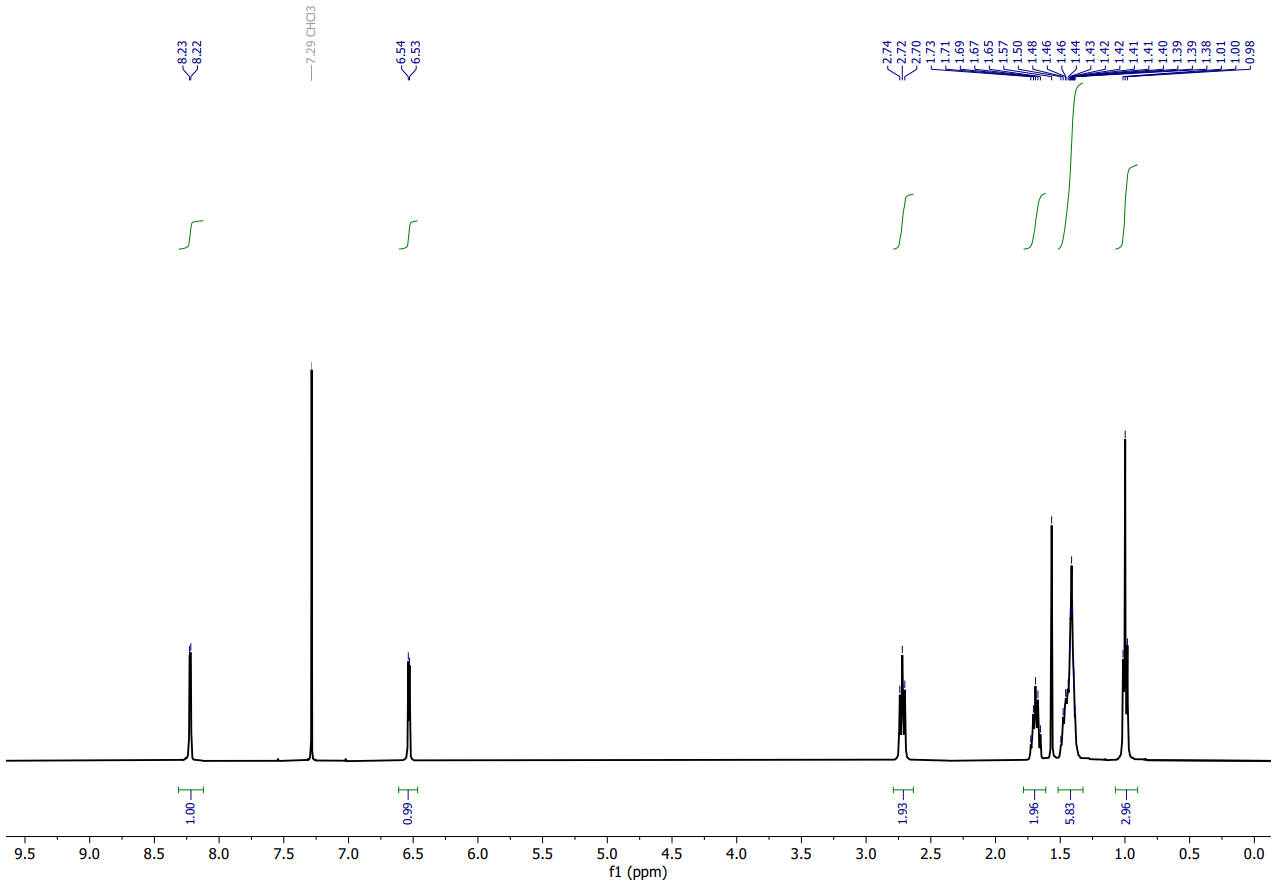


**Figure S9.** ^1^H NMR spectrum (400 MHz, chloroform-*d*) of **1** (298 K) after the powder sample was stored for 4 months under ambient conditions.


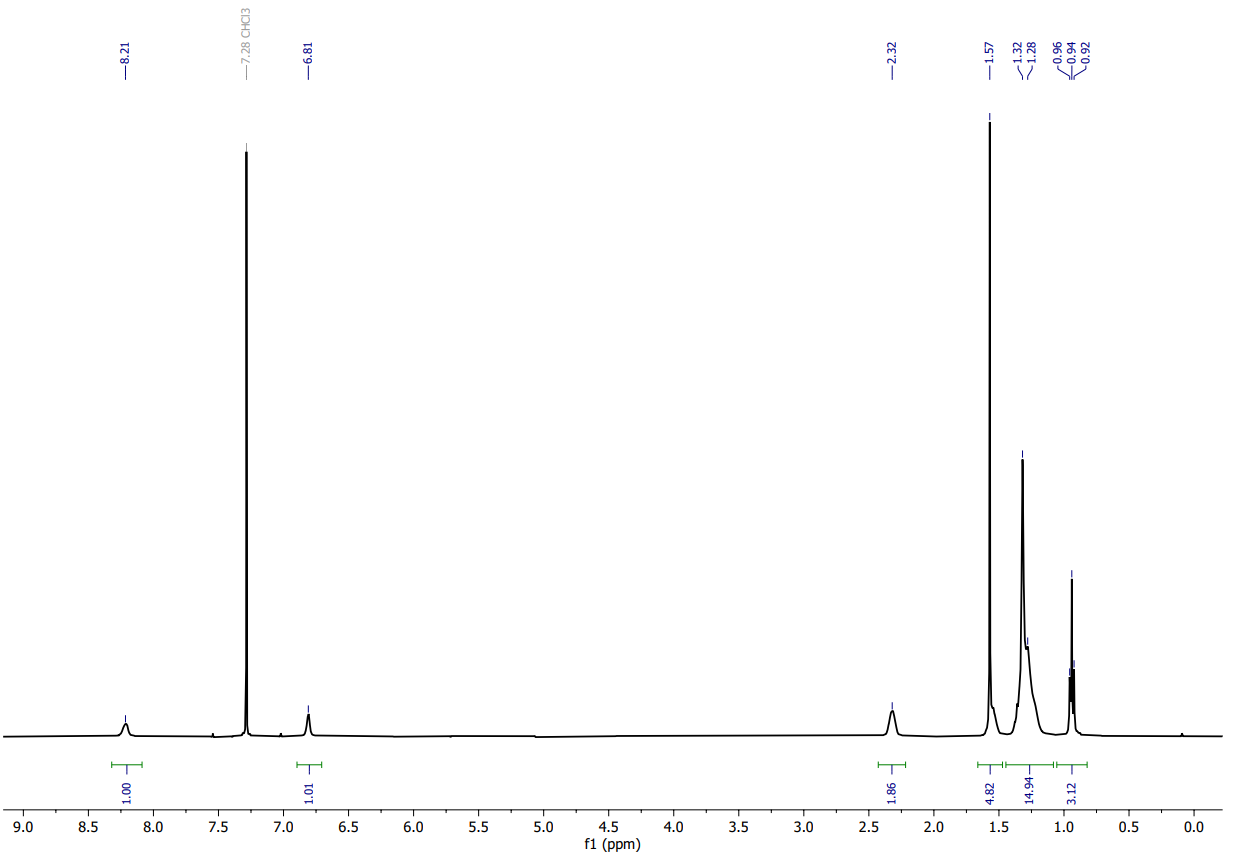


**Figure S10.** ^1^H NMR spectrum (400 MHz, chloroform-*d*) of **2** (298 K) after the powder sample was stored for 5 months under ambient conditions.

**
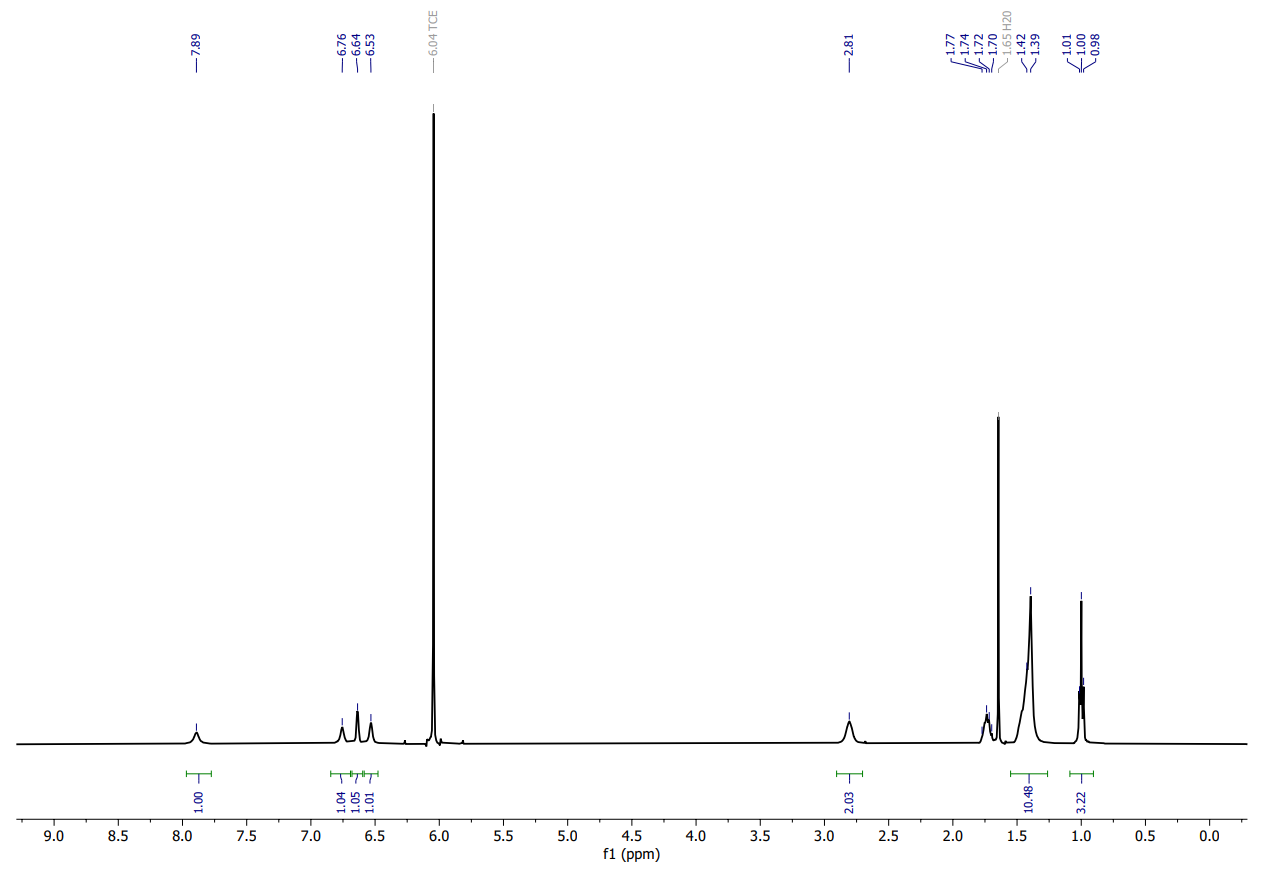
**

**Figure S11.** ^1^H NMR spectrum (400 MHz, 1,1,2,2-tetrachloroethane-*d_2_*) of **3** (298 K) after the powder sample was stored for 4 months under ambient conditions.


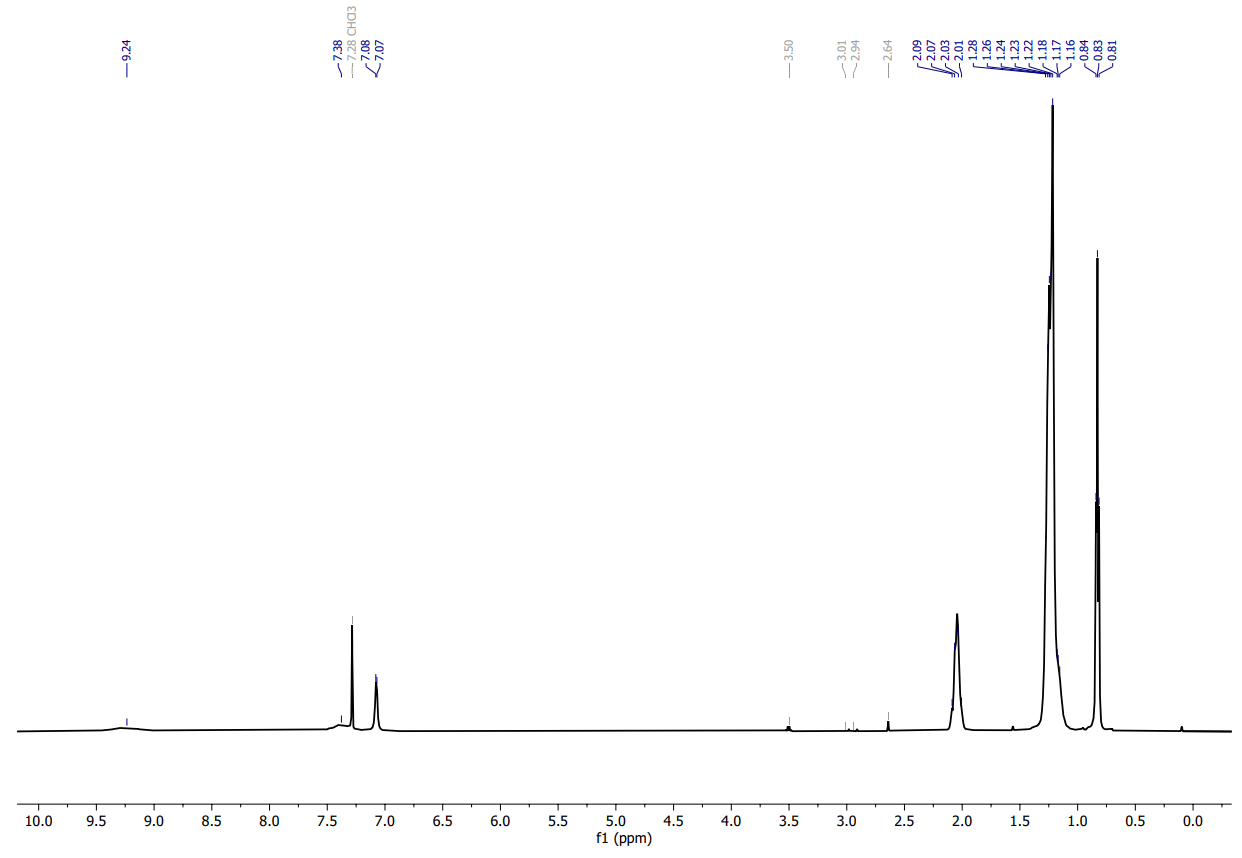


**Figure S12.** ^1^H NMR spectrum (400 MHz, chloroform-*d*) of **4** (298 K) after the powder sample was stored for 4 months under ambient conditions.

**
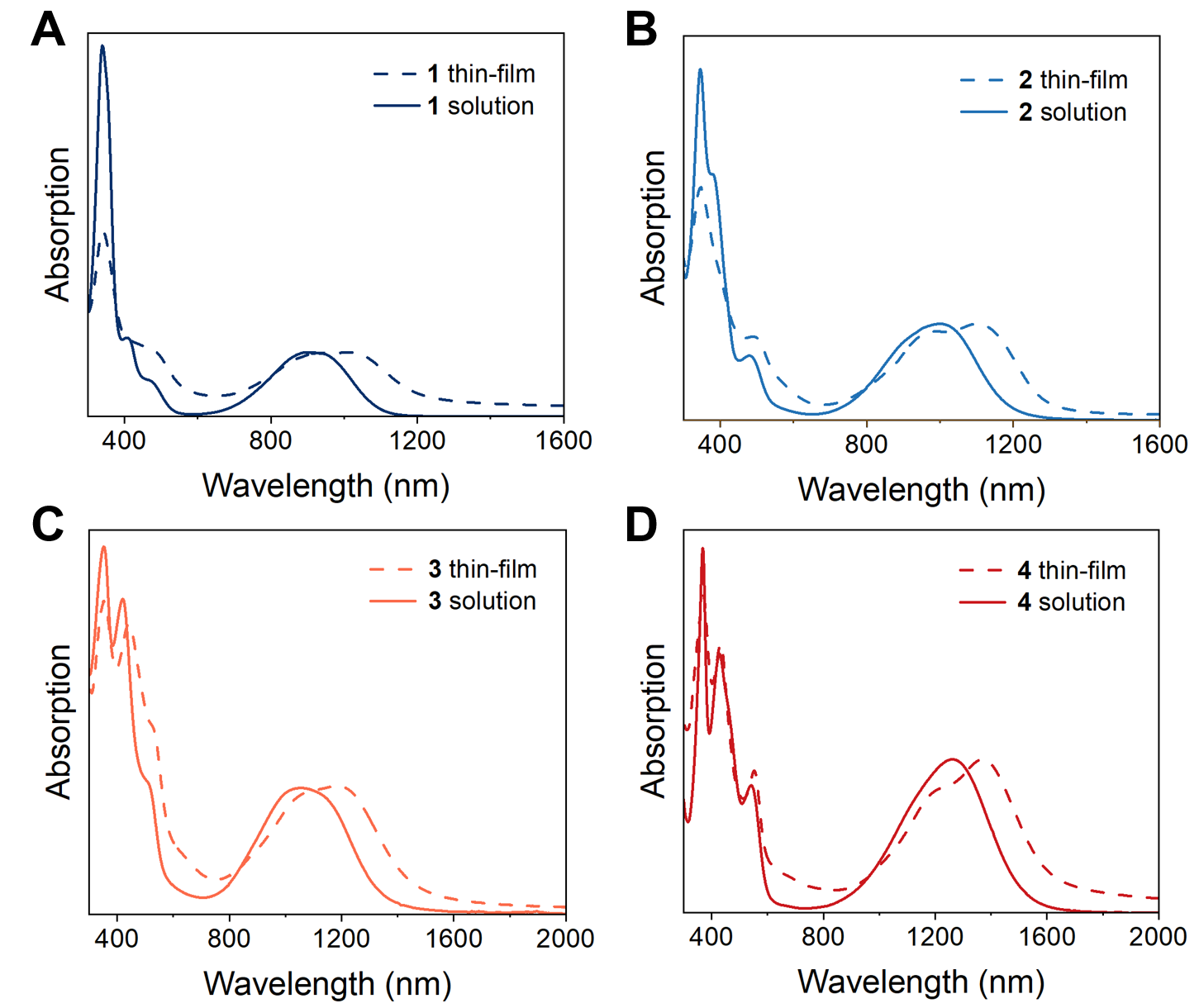
**

**Figure S13.** Normalized solution (chloroform, ~10^-5^ M) and thin-film absorption spectra for **1-4**.


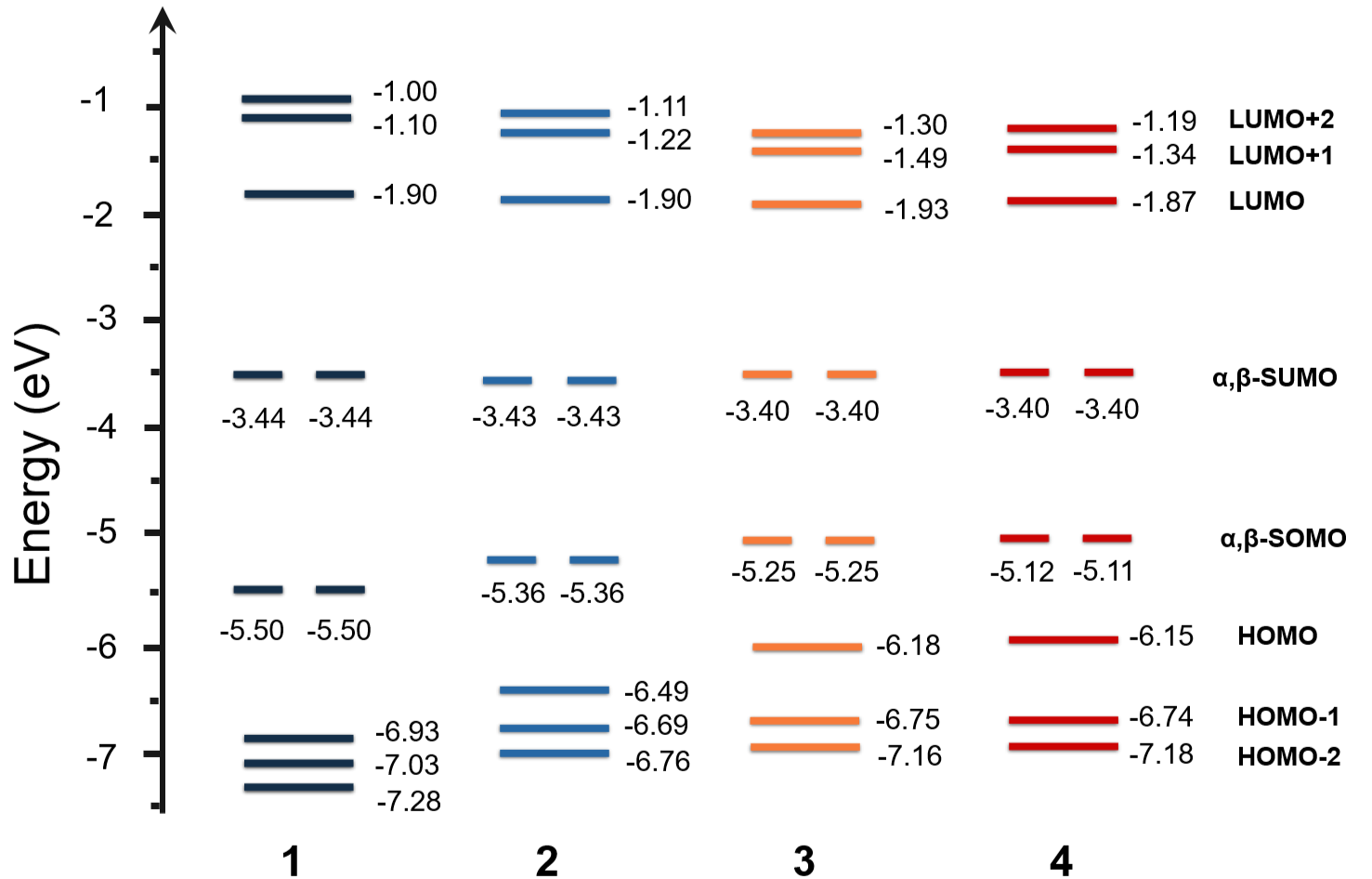


**Figure S14.** Schematic MO diagram illustrating the energy levels of the FMOs of **1-4** predicted using broken symmetry calculations with BS-DFT with the (U)CAM-B3LYP functional and def2-TZVP basis set.


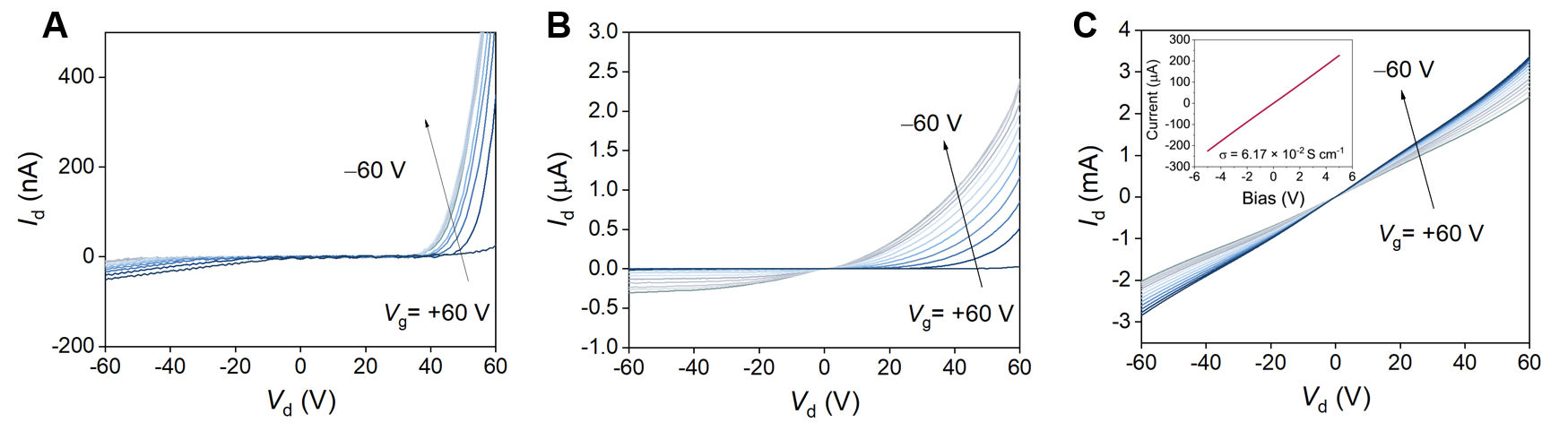


**Figure S15**. Representative field effect transistor output characteristics of (**A**) **2**, (**B**) **3**, and (**C**) **4** (inset: Current-voltage characteristics).


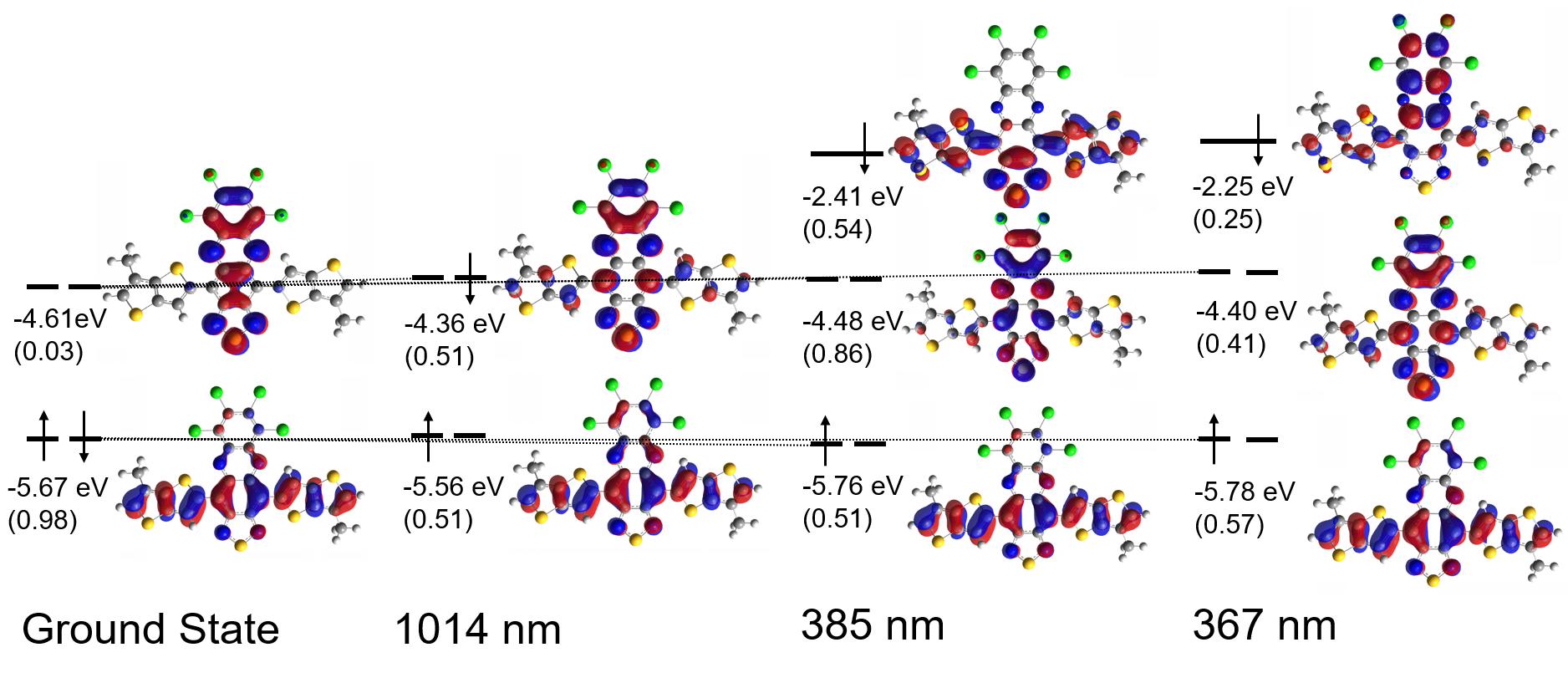


**Figure S16.** DO representations and energies of the major transitions calculated by (U)CAM-B3LYP/def2-TZVP with MRSF-TDDFT absorption spectrum of **2** with the normalized occupancies in parenthesis.


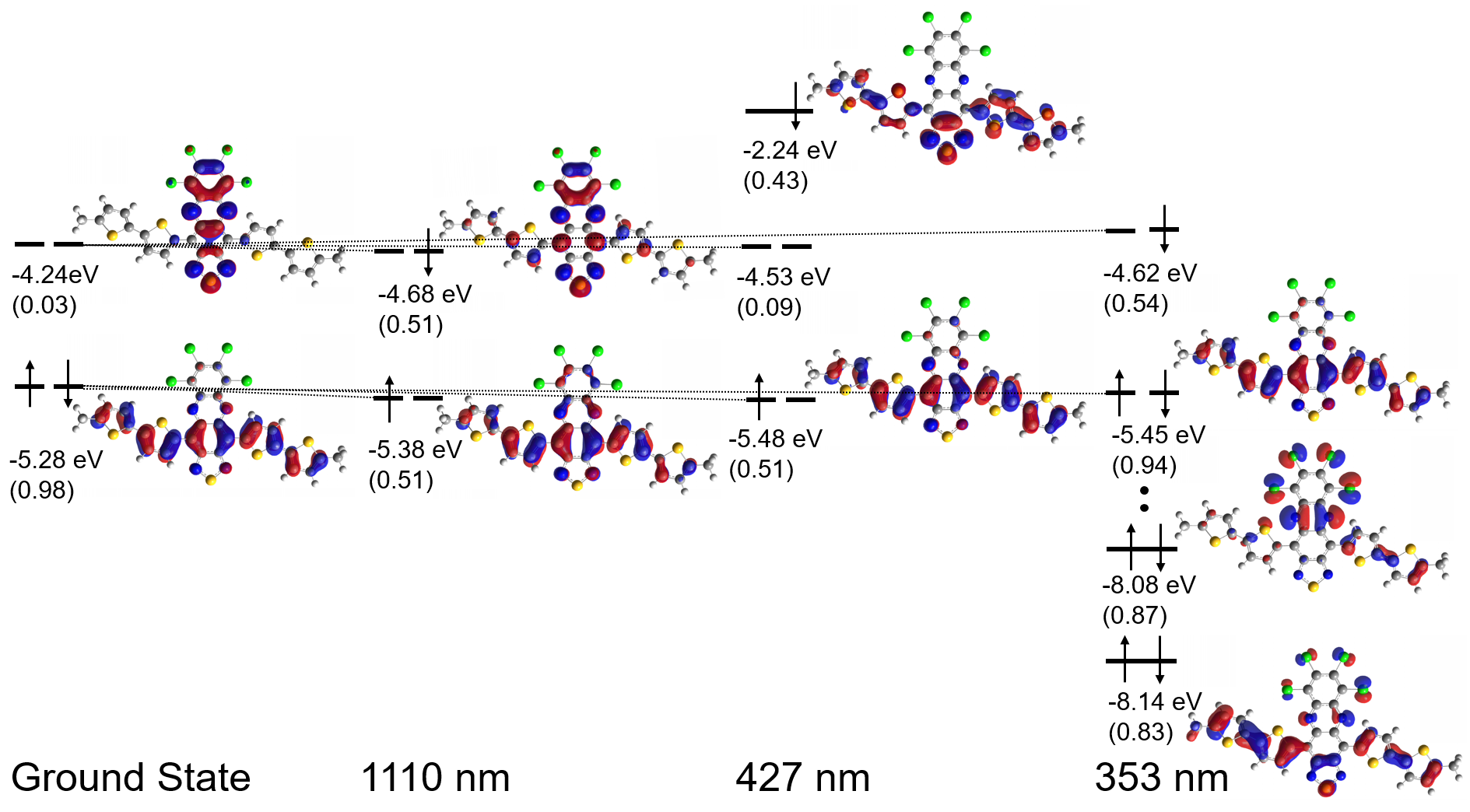


**Figure S17.** DO representations and energies of the major transitions calculated by (U)CAM-B3LYP/def2-TZVP with MRSF-TDDFT absorption spectrum of **3** with the normalized occupancies in parenthesis.


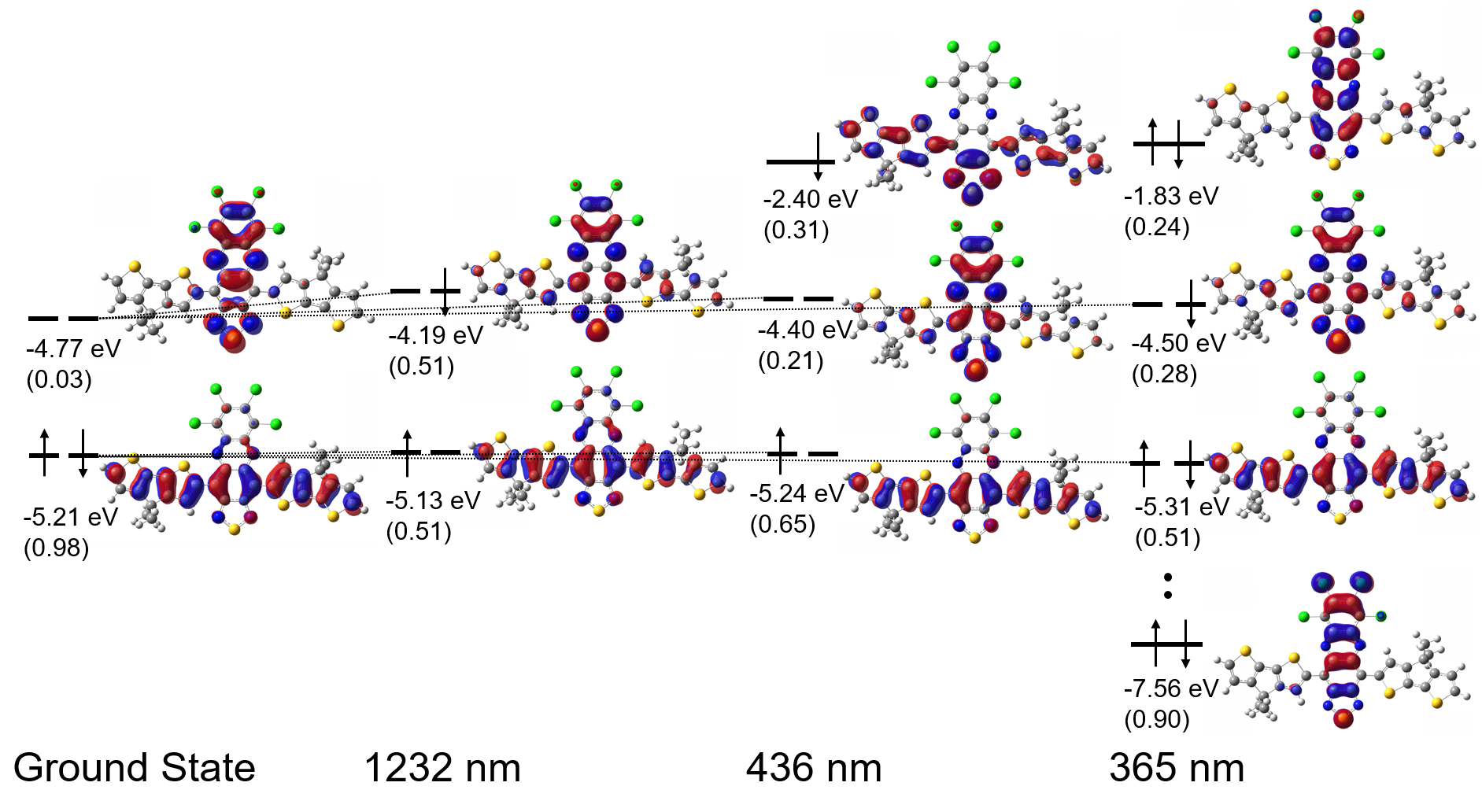


**Figure S18.** DO representations and energies of the major transitions calculated by (U)CAM-B3LYP/def2-TZVP with MRSF-TDDFT absorption spectrum of **4** with the normalized occupancies in parenthesis.


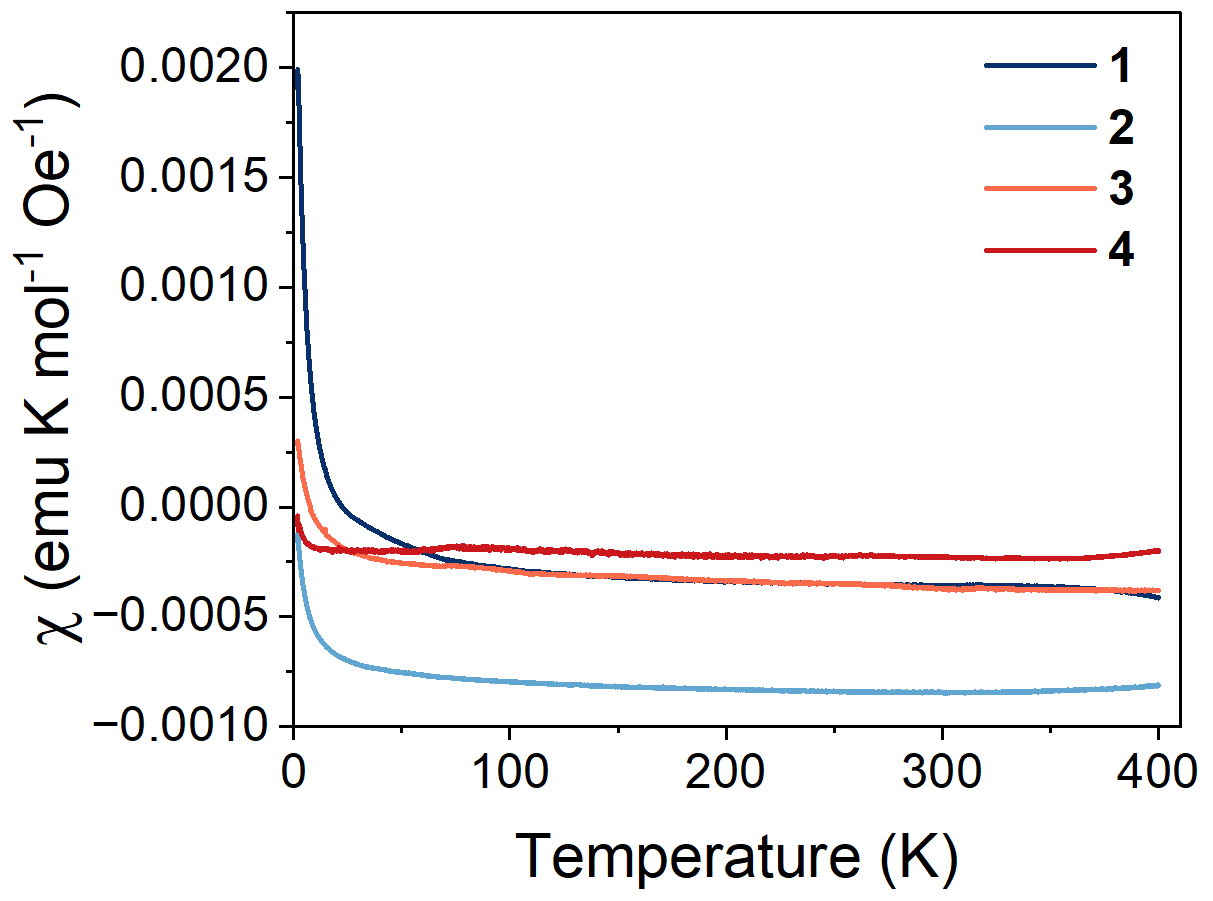


**Figure S19.** Magnetic susceptibility, χ versus T, from 2 to 400 K of powder samples.


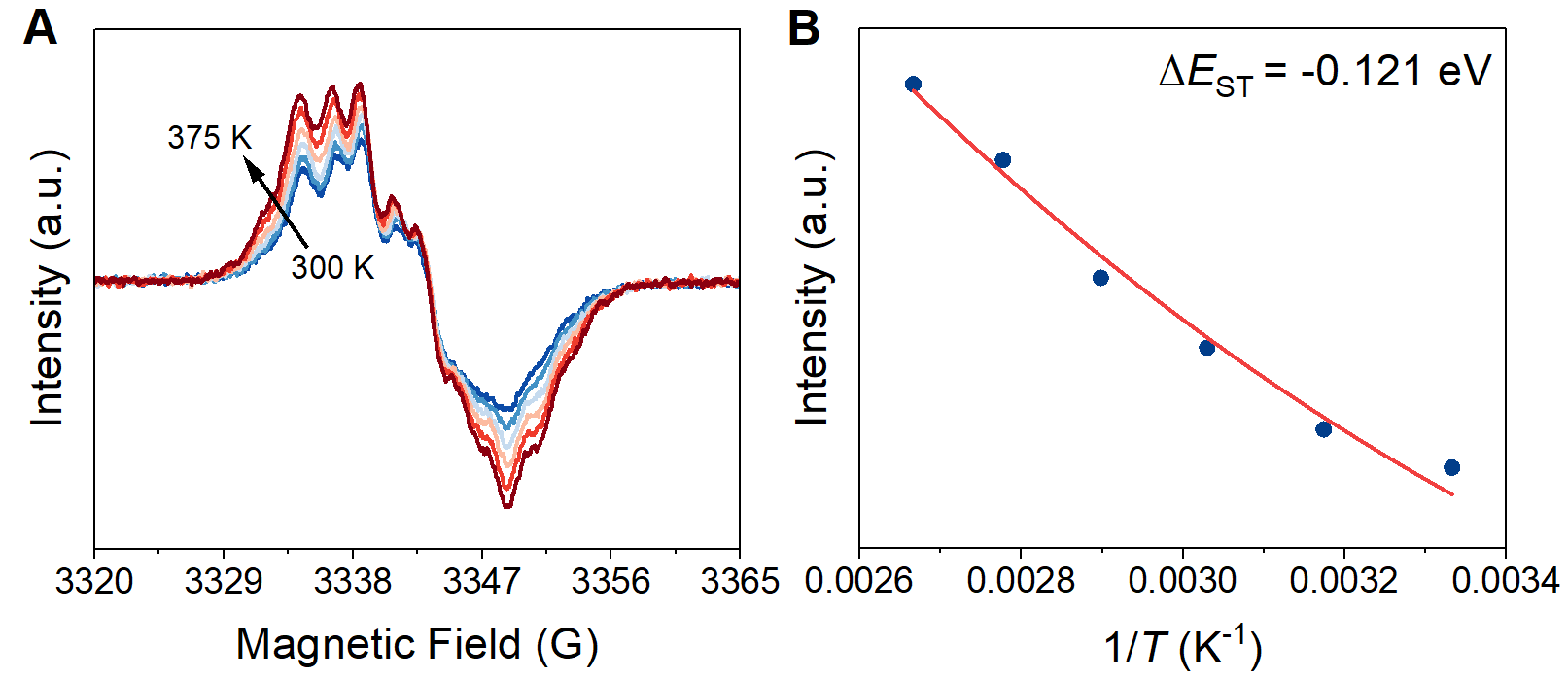


**Figure S20.** (**A**) VT EPR measurements of **1** in toluene between 300-375 K. (**B**) Temperature-dependent fit to the Bleaney-Bowers equation with ΔE_ST_ of -0.121 eV.


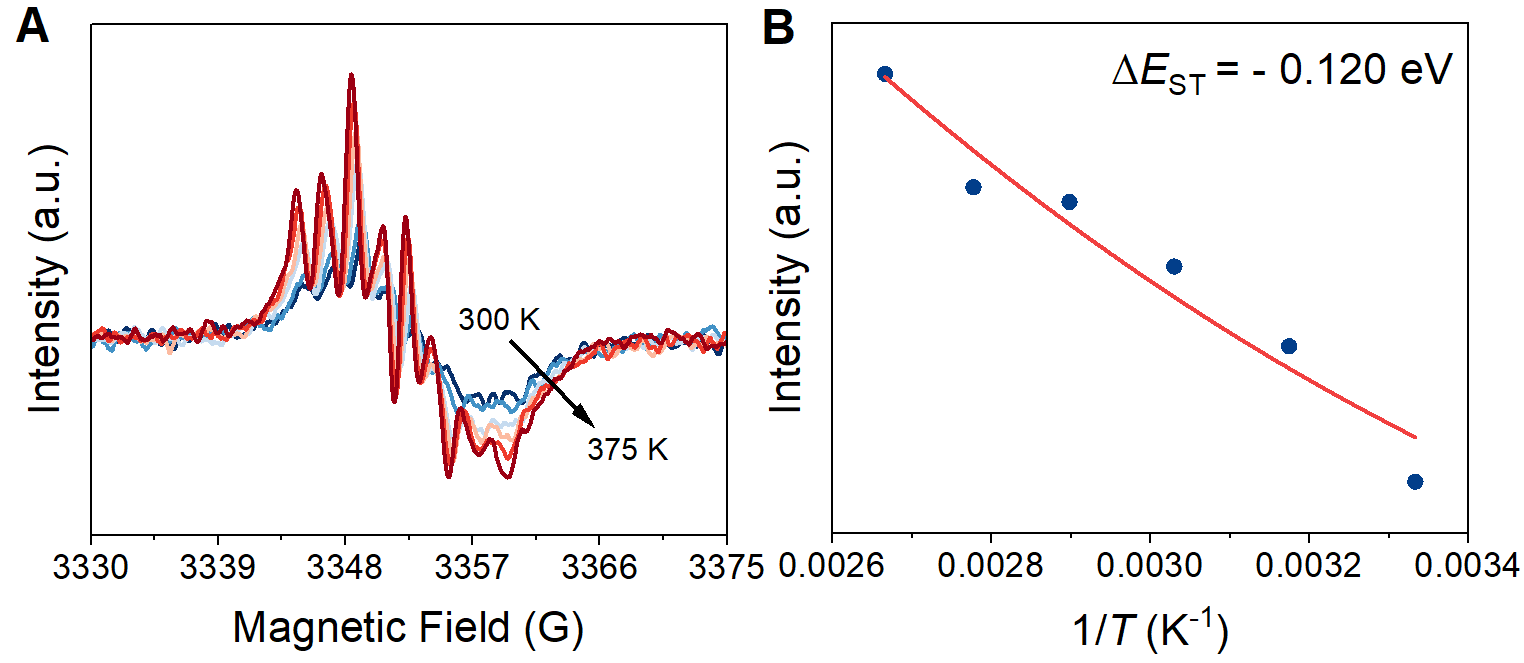


**Figure S21.** (**A**) VT EPR measurements of **2** in toluene between 300-375 K. (**B**) Temperature-dependent fit to the Bleaney-Bowers equation with ΔE_ST_ of -0.120 eV.


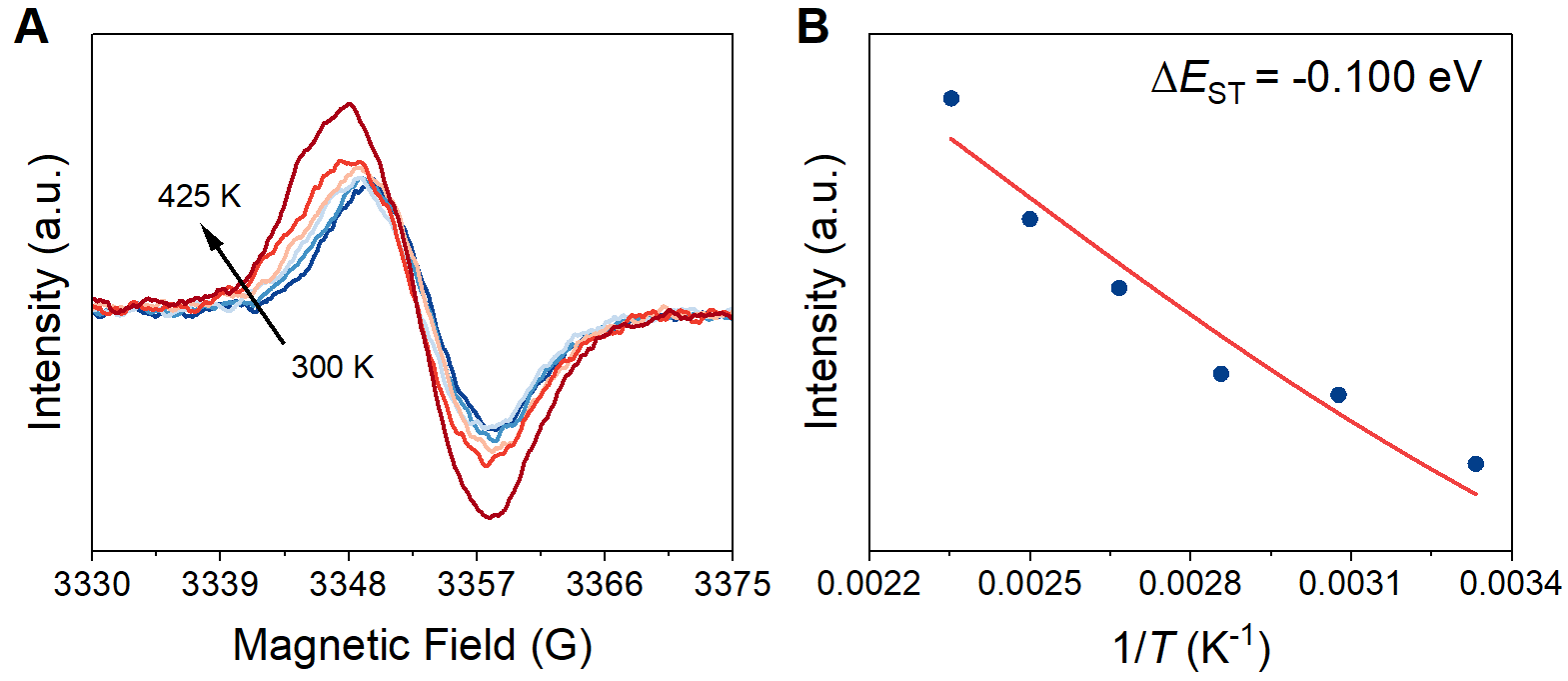


**Figure S22.** (**A**) VT EPR measurements of **3** in bromobenzene between 300-425 K. (**B**) Temperature-dependent fit to the Bleaney-Bowers equation with ΔE_ST_ of -0.100 eV.


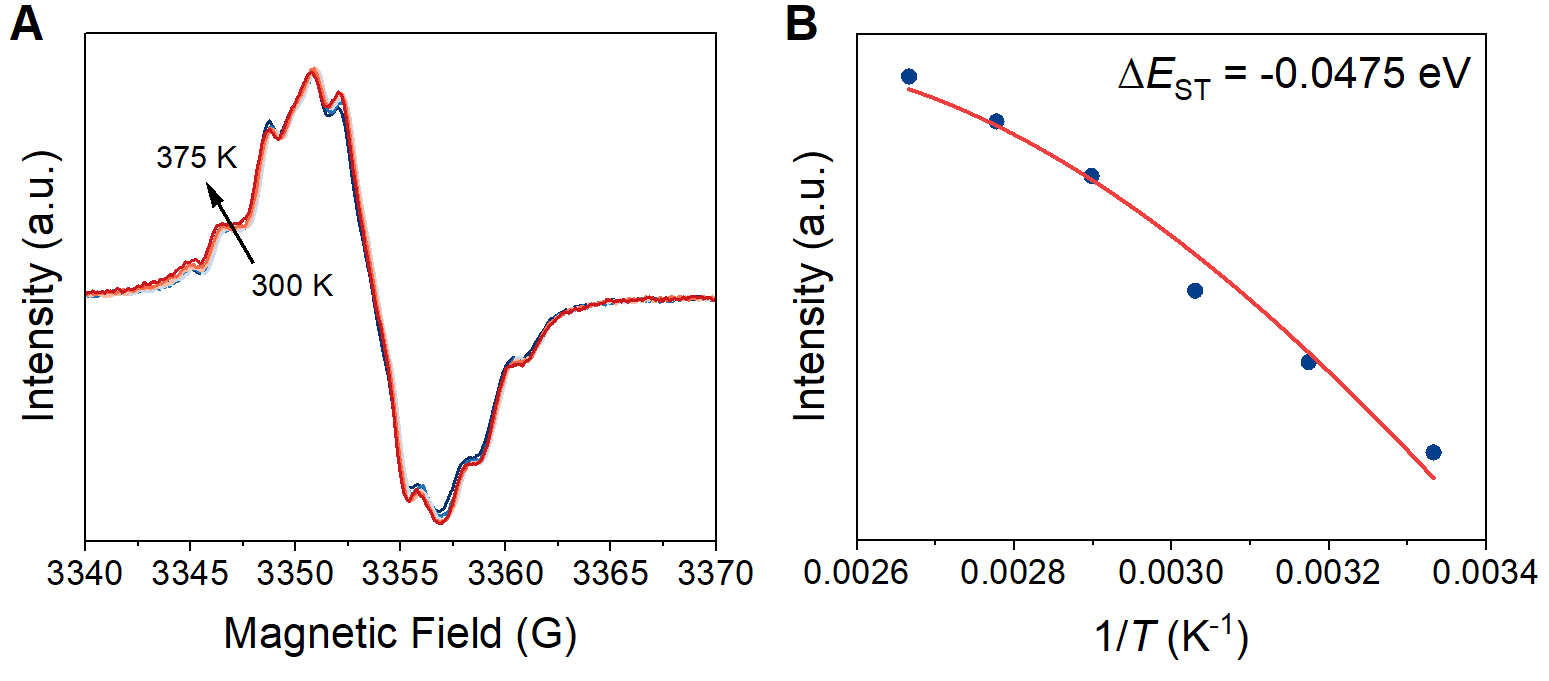


**Figure S23.** (**A**) VT EPR measurements of **4** in toluene between 300-375 K. (**B**) Temperature-dependent fit to the Bleaney-Bowers equation with ΔE_ST_ of -0.0475 eV.


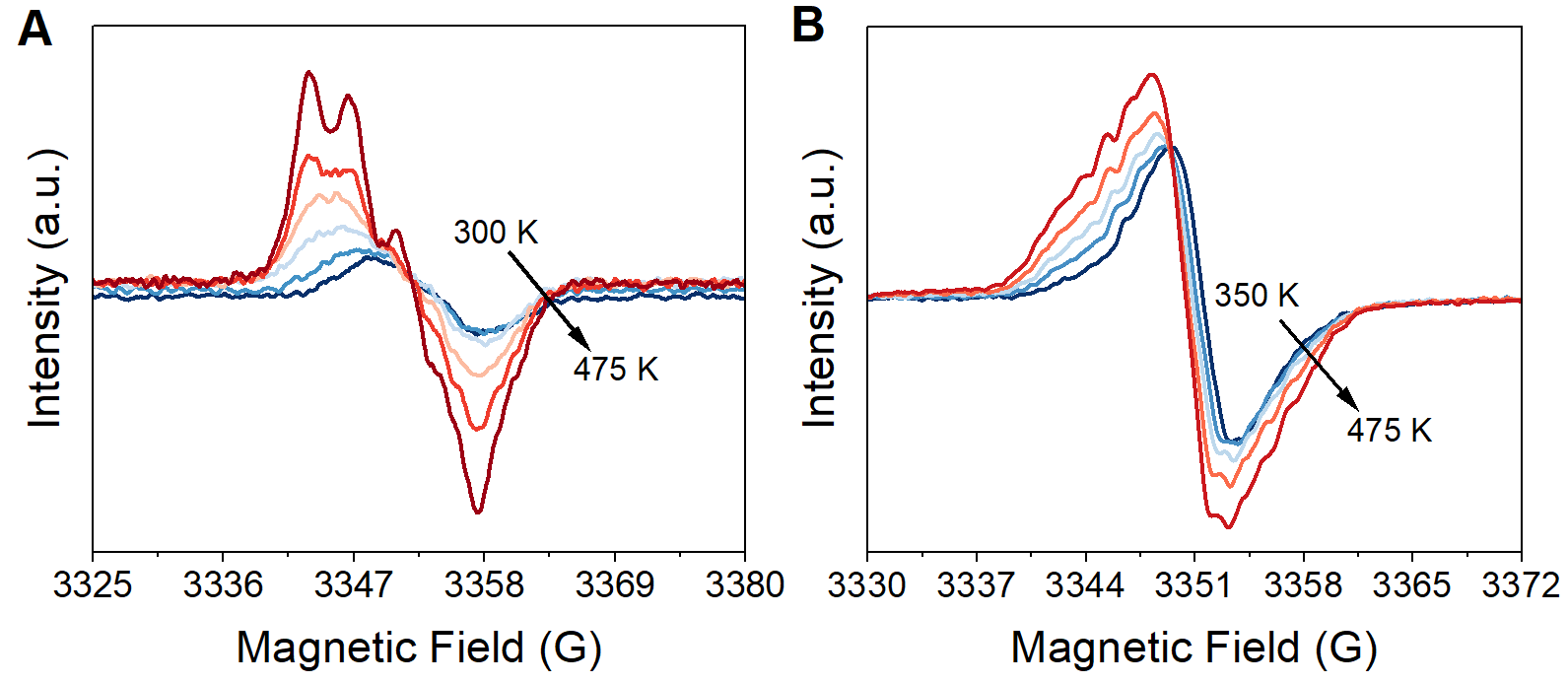


**Figure S24.** VT EPR measurements of **3** (**A**) and **4** (**B**) in CN between 300-475 K.


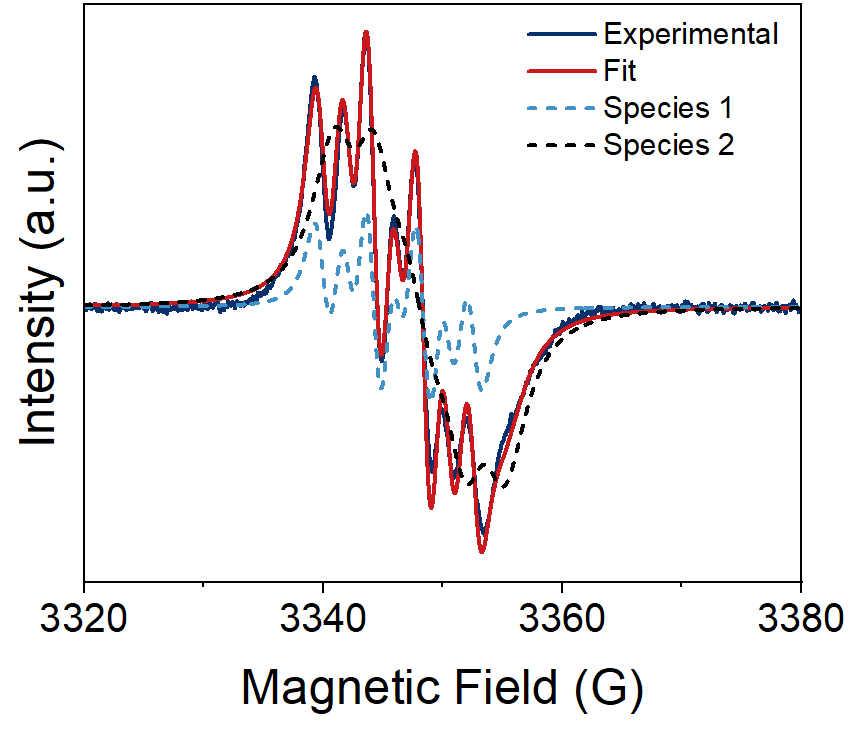


**Figure S25.** Simulation of **1** at 475 K in CN. Two S = 1 species were modeled with hyperfine couplings to two ^14^N nuclei and two ^1^H nuclei.


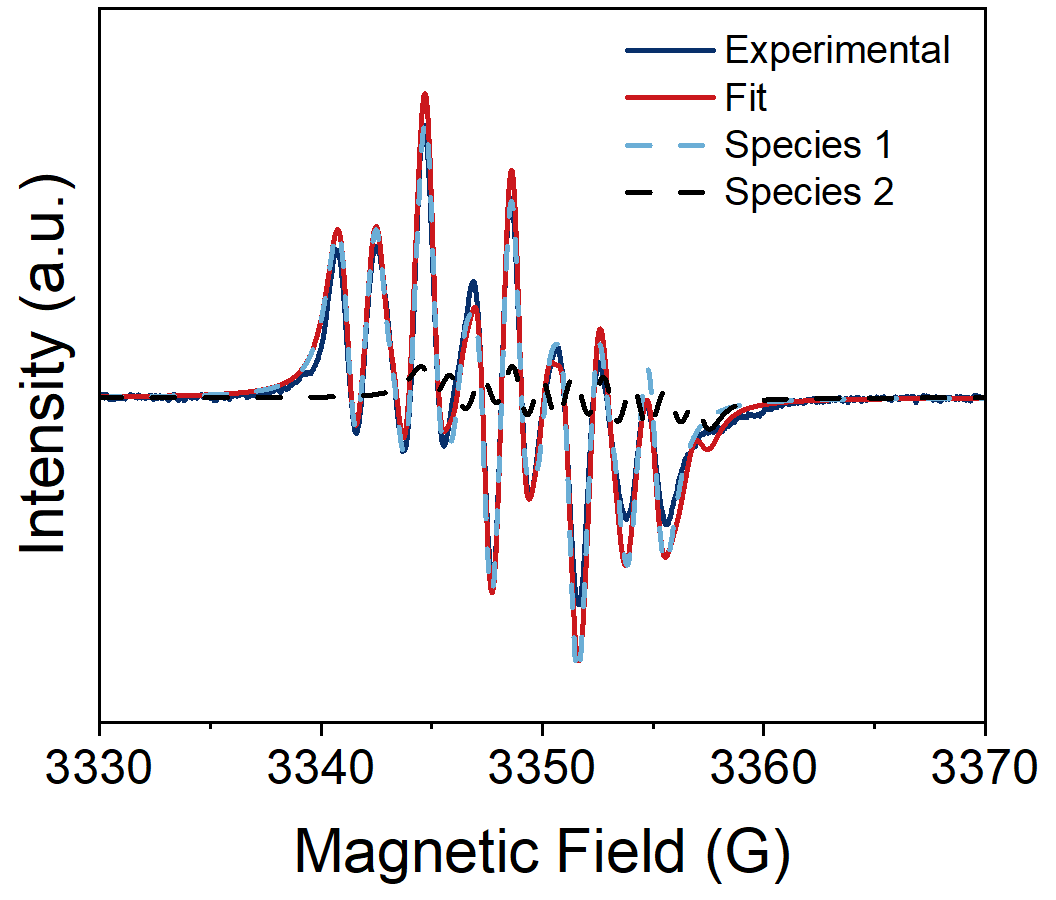


**Figure S26.** Simulation of **2** at 475 K in CN. Two S = 1 species were modeled with hyperfine couplings to two ^14^N nuclei and one ^1^H nucleus.


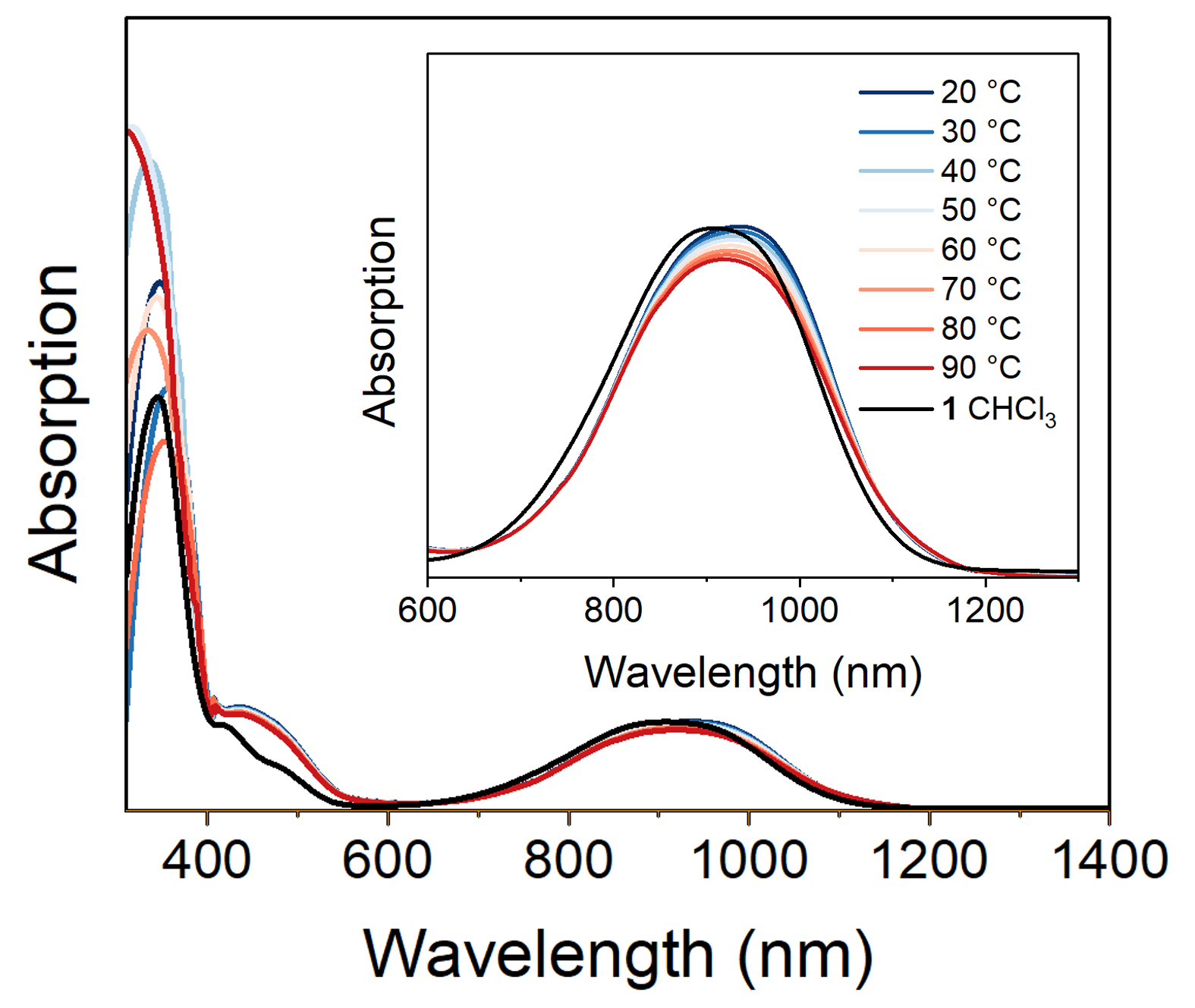


**Figure S27.** UV-Vis-NIR of **1** in CN (0.1 mg/ml) between 20-90 °C plotted vs. the CHCl_3_ spectrum of **1** (0.1 mg mL^-1^) at 25 °C.


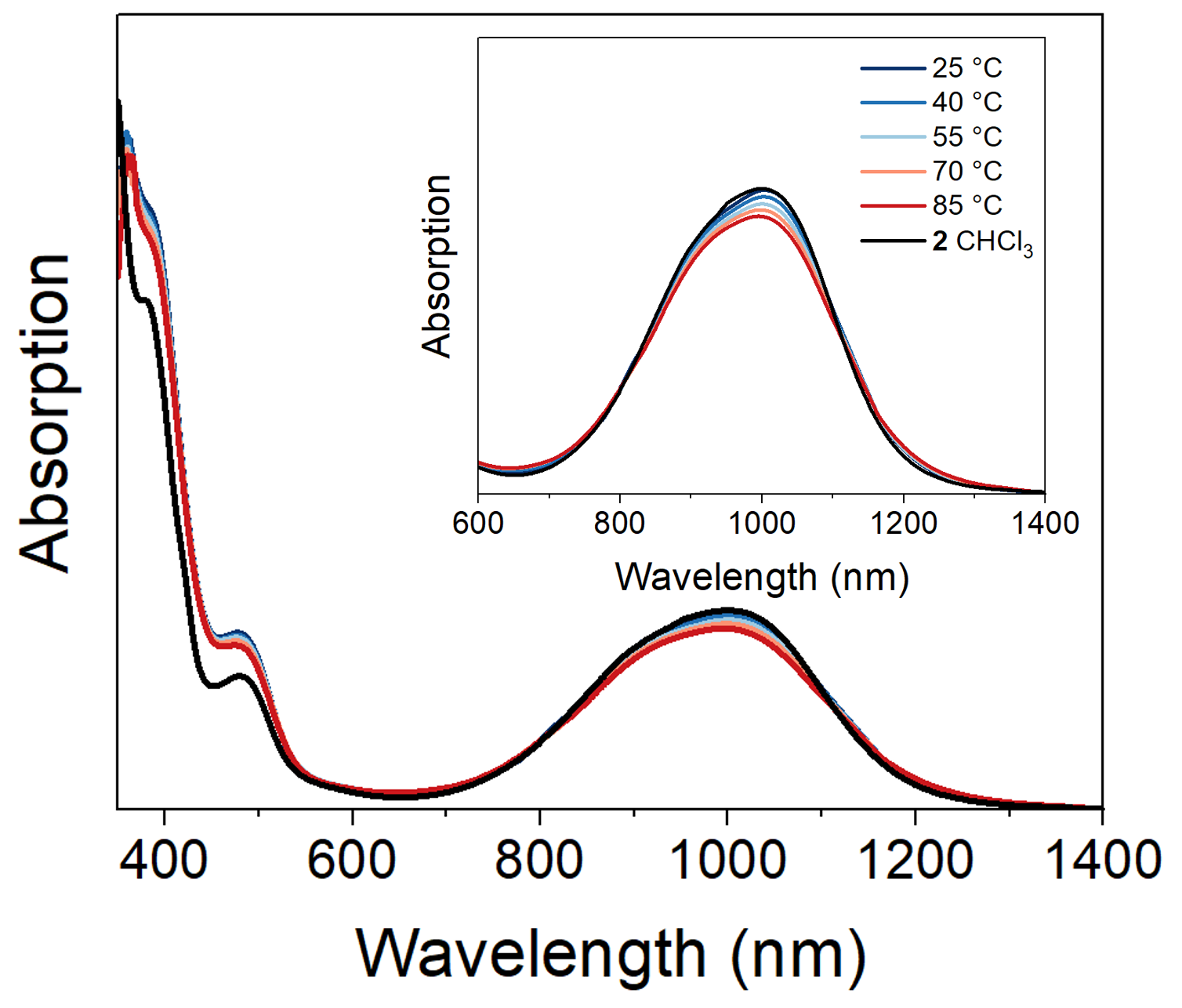


**Figure S28.** UV-Vis-NIR of **2** in CN (0.1 mg mL^-1^) between 25-85 °C plotted vs. the CHCl_3_ spectrum of **2** (0.1 mg mL^-1^) at 25 °C.


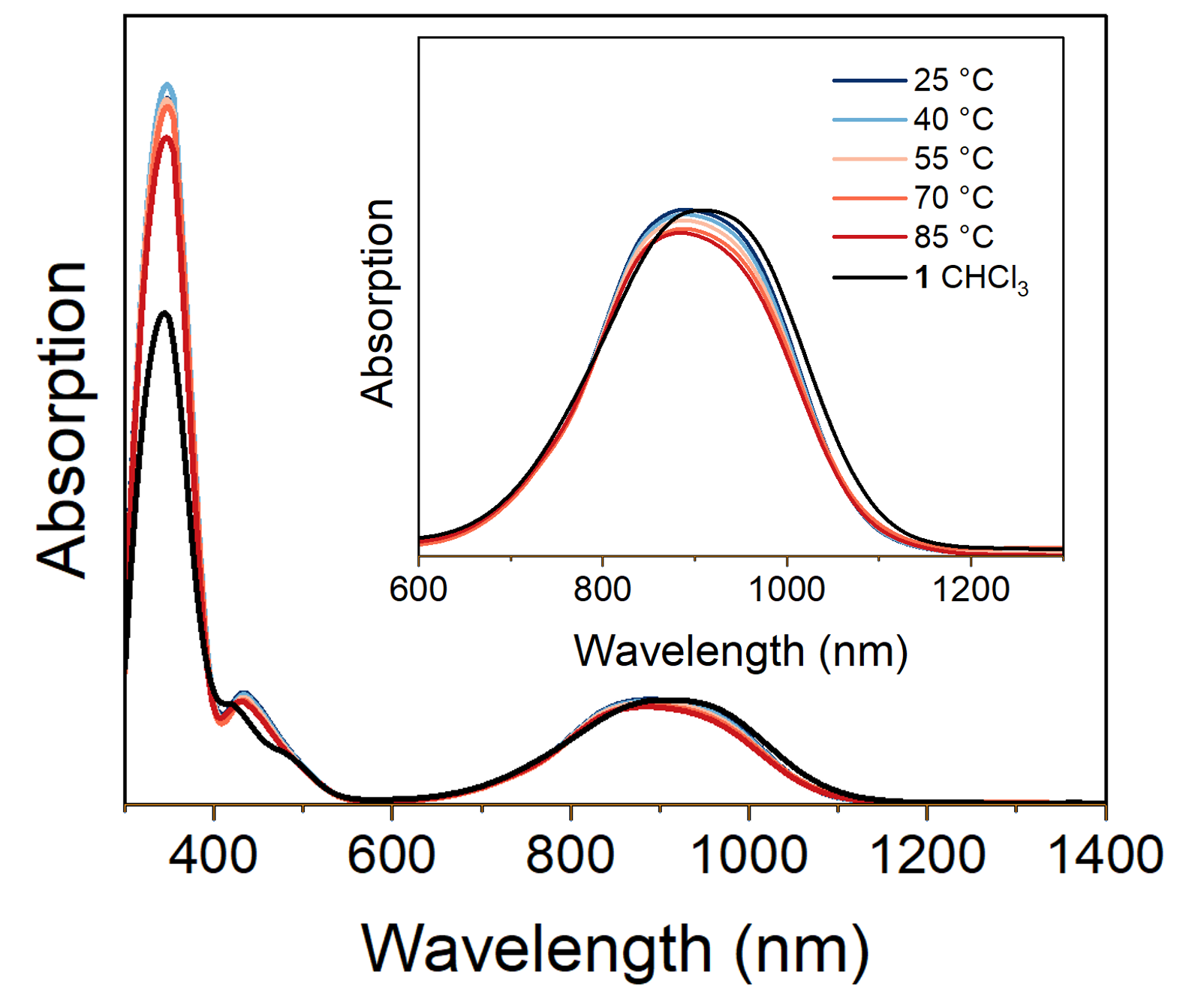


**Figure S29.** UV-Vis-NIR of **1** in toluene (0.1 mg mL^-1^) between 25-85 °C plotted vs. the CHCl_3_ spectrum of **1** (0.1 mg mL^-1^) at 25 °C.


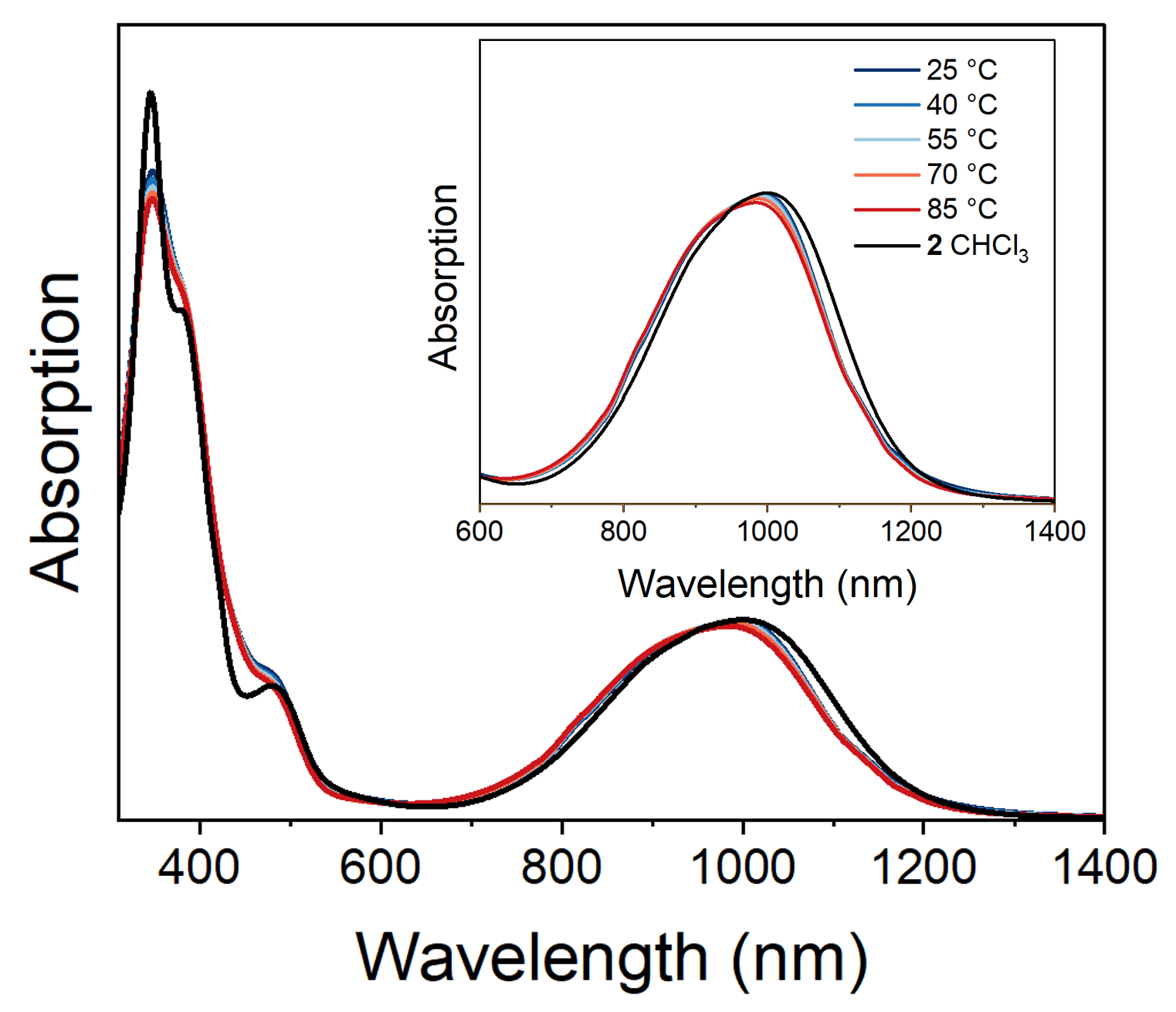


**Figure S30.** UV-Vis-NIR of **2** in toluene (0.1 mg mL^-1^) between 25-85 °C plotted vs. the CHCl_3_ spectrum of **1** (0.1 mg mL^-1^) at 25 °C.


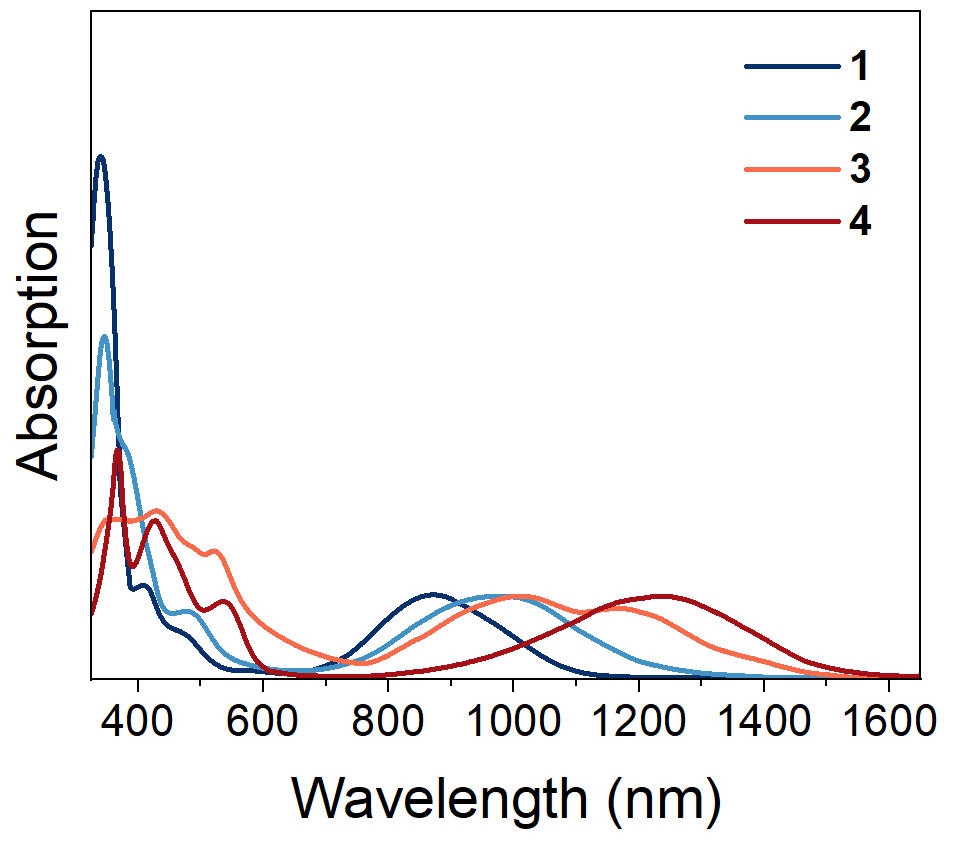


**Figure S31.** UV-Vis-NIR of **1-4** in DCM (0.1 mg mL^-1^) at 25 °C.


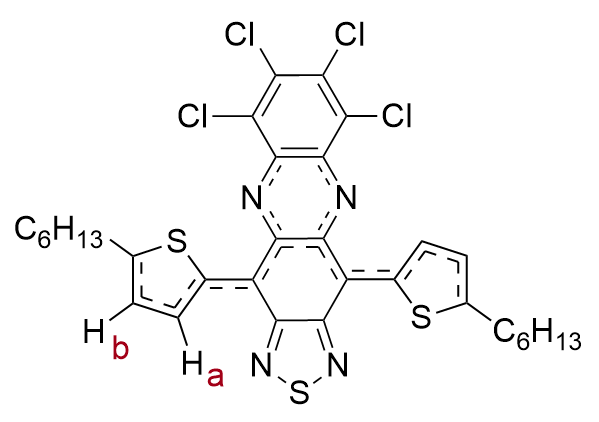


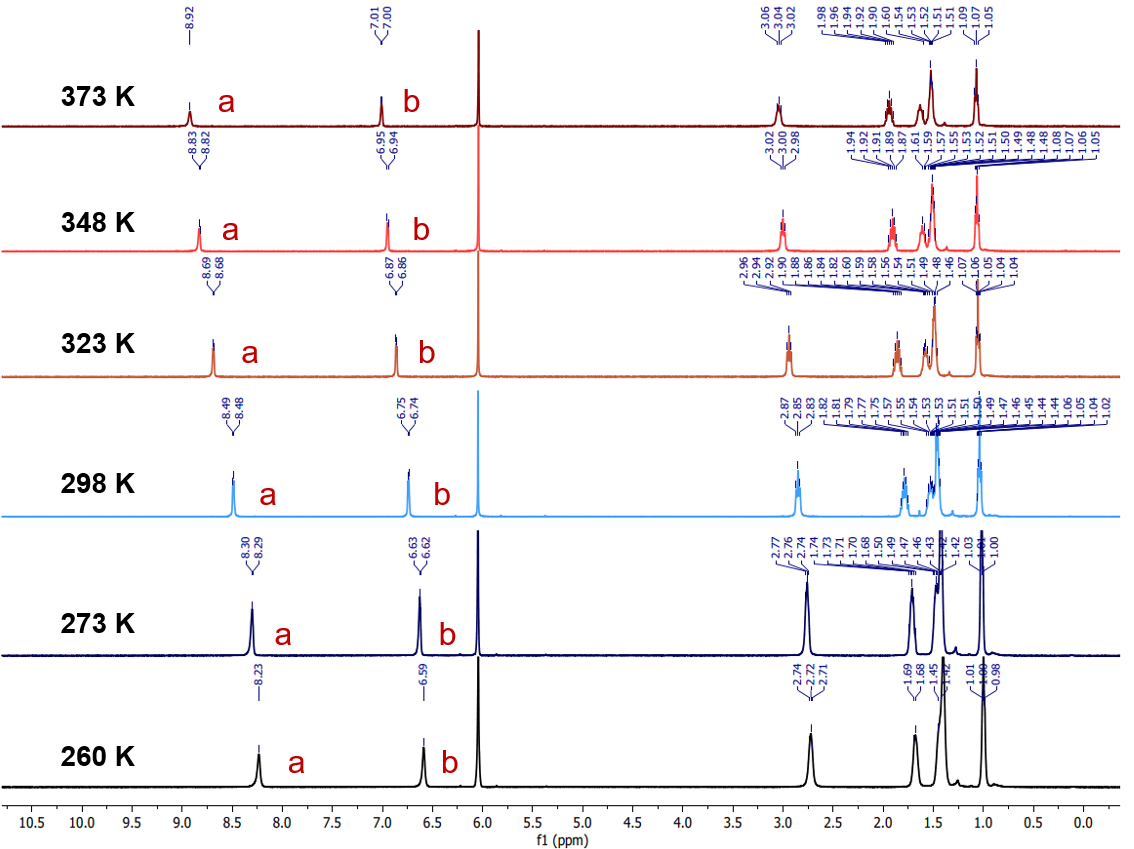


**Figure S32.** VT ^1^H NMR spectrum (400 MHz, tetrachloroethane-d_2_) of **1.**


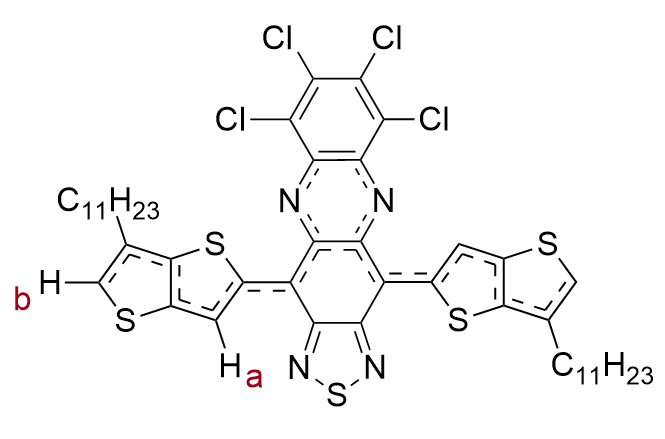


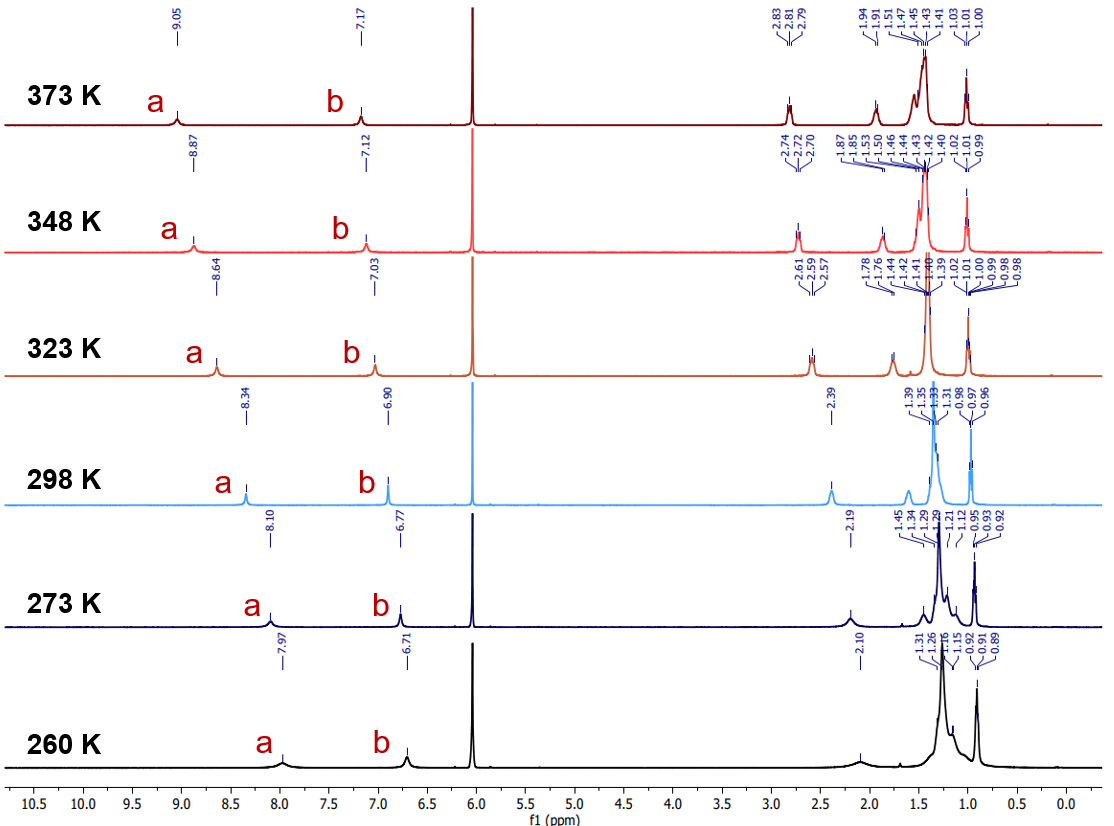


**Figure S33.** VT ^1^H NMR spectrum (400 MHz, tetrachloroethane-d_2_) of **2.**

**
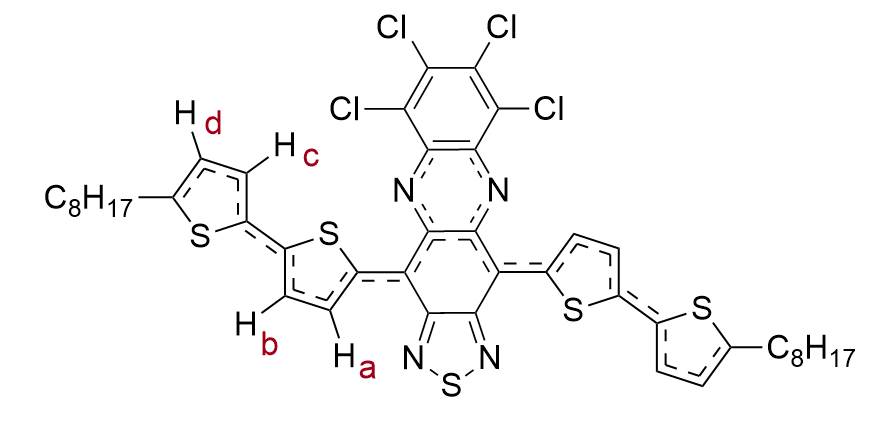
**

**
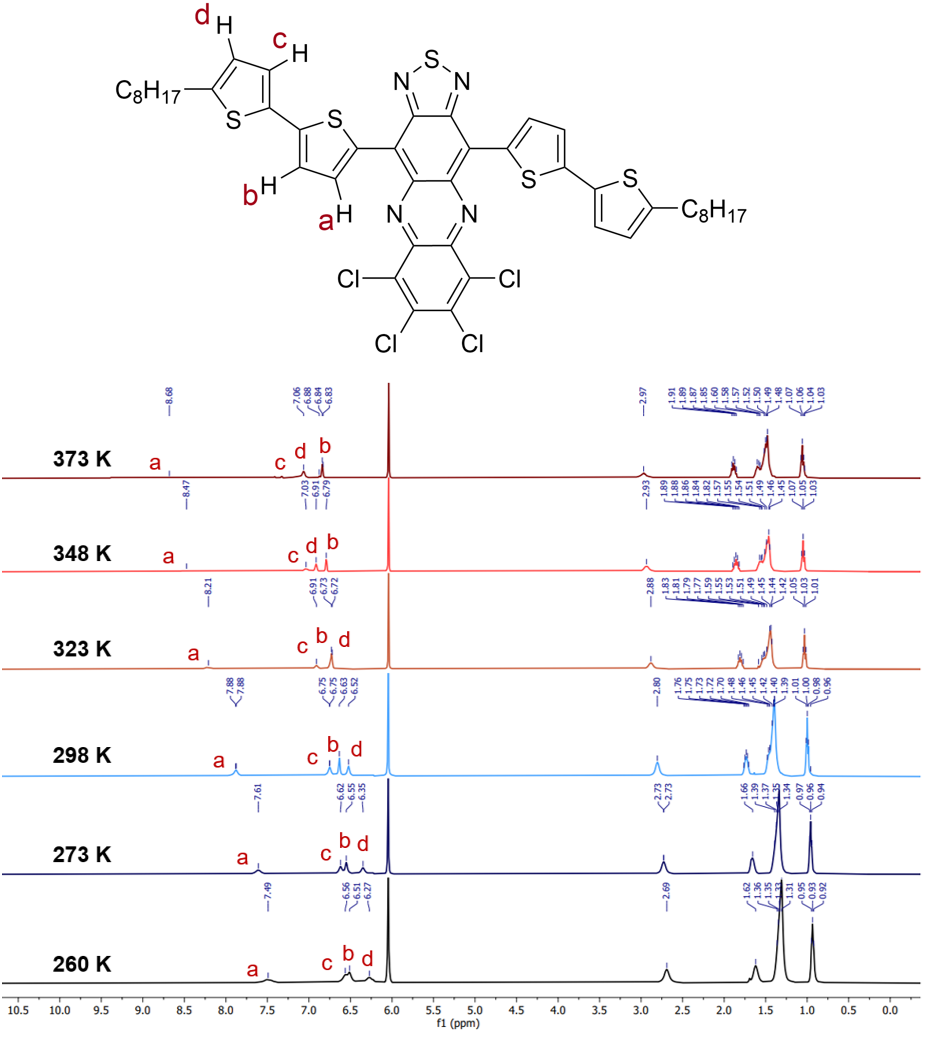
**

**Figure S34.** VT ^1^H NMR spectrum (400 MHz, tetrachloroethane-d_2_) of **3.**


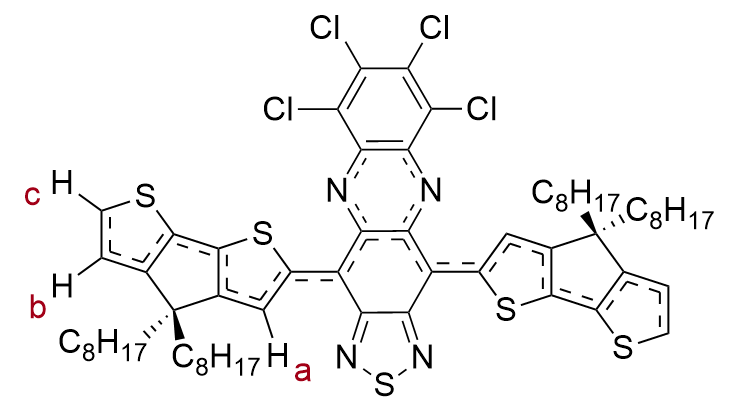


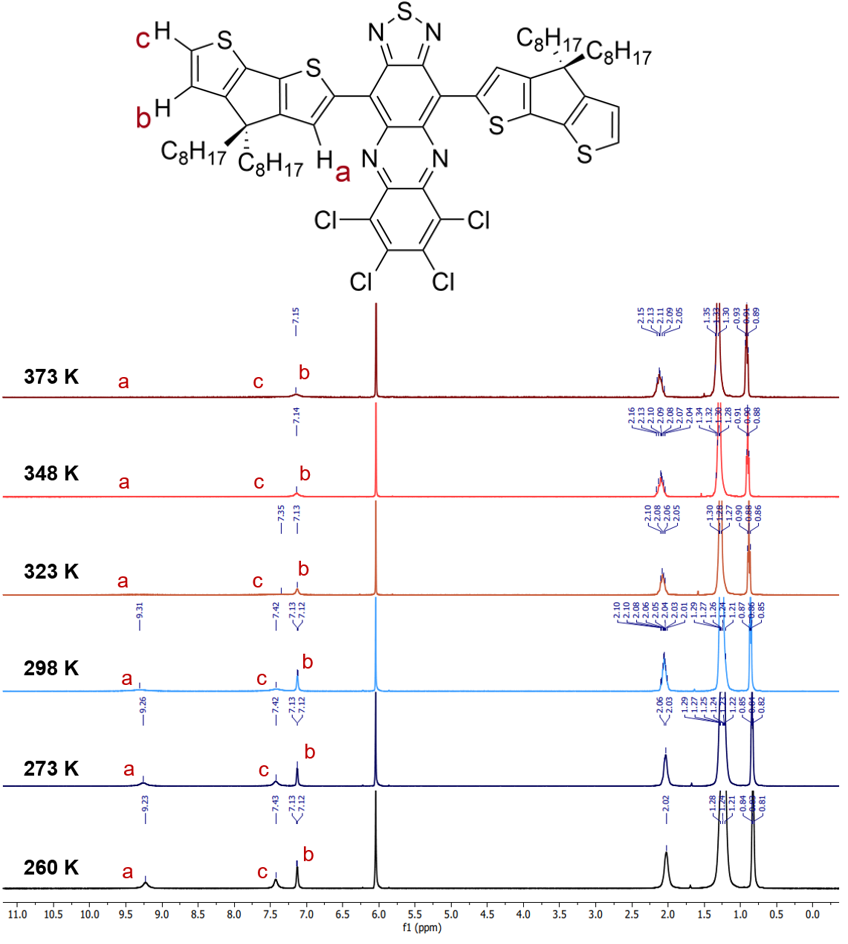


**Figure S35.** VT ^1^H NMR spectrum (400 MHz, tetrachloroethane-d_2_) of **4.**


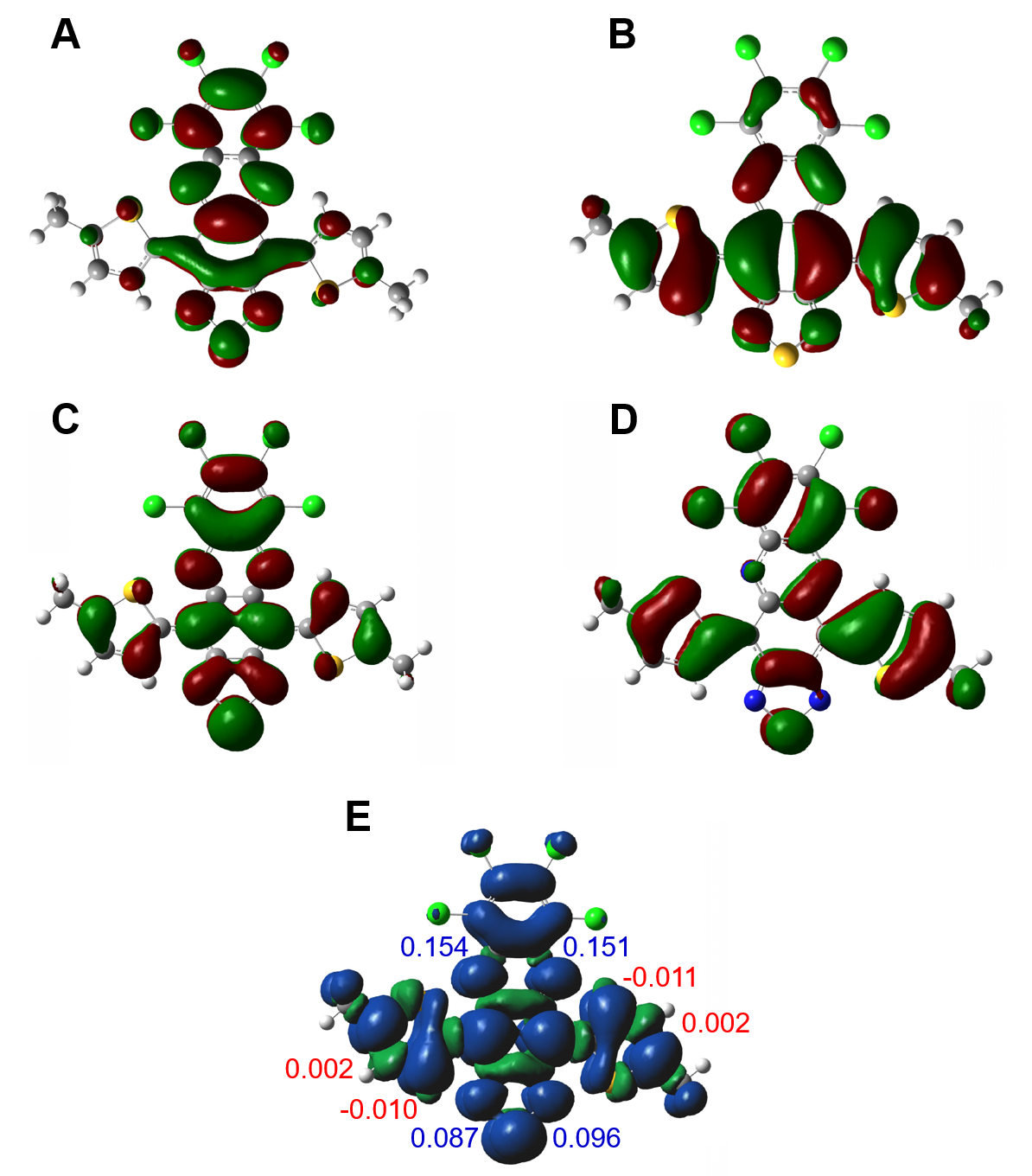


**Figure S36.** Optimized open-shell triplet ground state geometric structures of **1** calculated with (U)CAM-B3LYP/Def2-TZVP and pictorial representations of the frontier MOs. (**A**) α-SUMO and (**B**) β-SUMO, (**C**) α-SOMO and (**D**) β-SOMO, and (**E**) Spin density distribution of the open-shell triplet with the spin populations for the respective nitrogen (blue) and hydrogen (red) nuclei. The green and red surfaces represent positive and negative signs of the MO at isovalue = 0.02 au, respectively. The blue and green surfaces represent positive and negative contributions of the spin density at an isovalue = 0.04 au. Color codes for the atoms are: gray for C, blue for N and yellow for S.


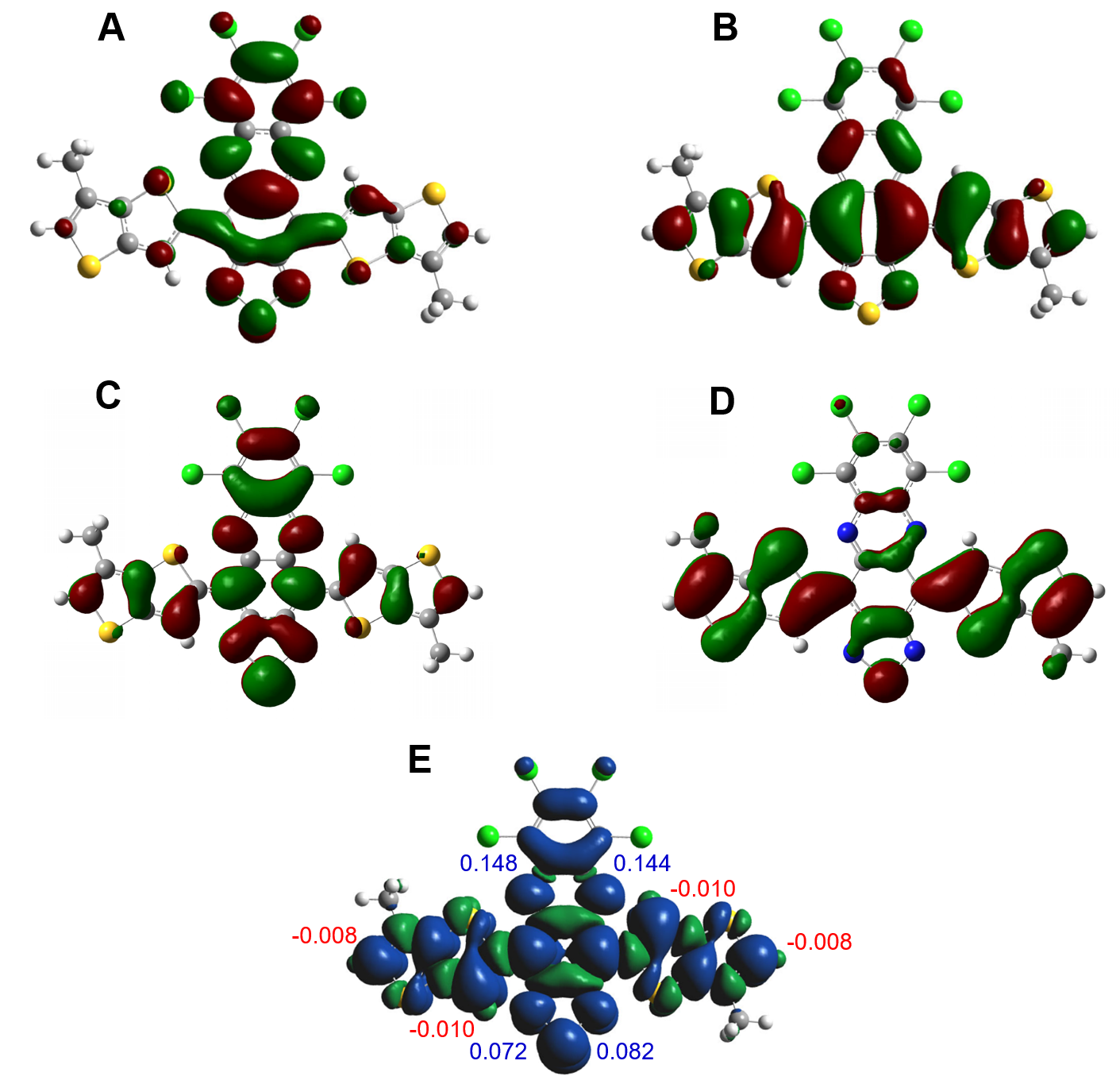


**Figure S37.** Optimized open-shell triplet ground state geometric structures of **2** calculated with (U)CAM-B3LYP/Def2-TZVP and pictorial representations of the frontier MOs. (**A**) α-SUMO and (**B**) β-SUMO, (**C**) α-SOMO and (**D**) β-SOMO, and (**E**) Spin density distribution of the open-shell triplet with the spin populations for the respective nitrogen (blue) and hydrogen (red) nuclei. The green and red surfaces represent positive and negative signs of the MO at isovalue = 0.02 au, respectively. The blue and green surfaces represent positive and negative contributions of the spin density at an isovalue = 0.04 au. Color codes for the atoms are: gray for C, blue for N and yellow for S.

**
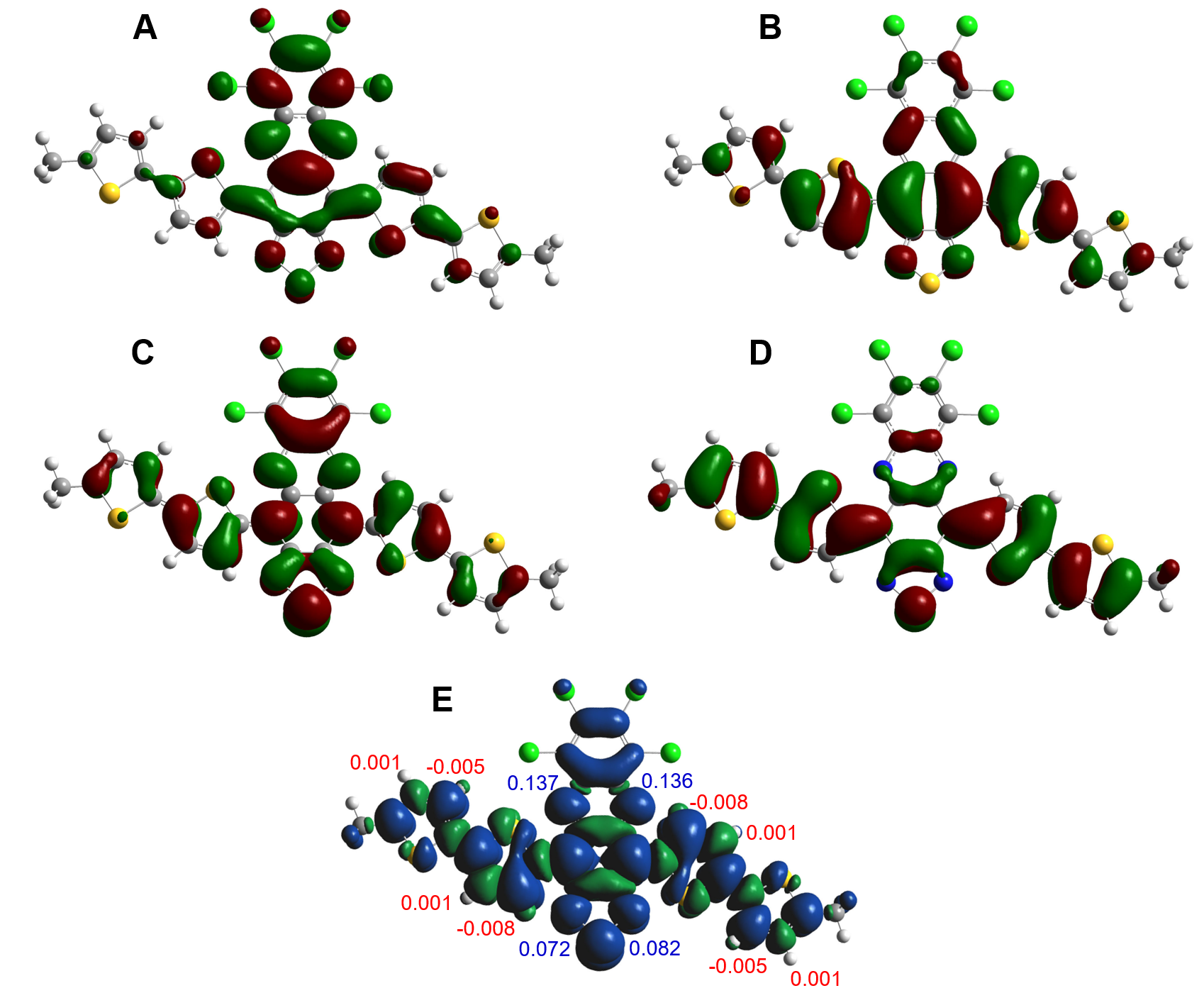
**

**Figure S38.** Optimized open-shell triplet ground state geometric structures of **3** calculated with (U)CAM-B3LYP/Def2-TZVP and pictorial representations of the frontier MOs. (**A**) α-SUMO and (**B**) β-SUMO, (**C**) α-SOMO and (**D**) β-SOMO, and (**E**) Spin density distribution of the open-shell triplet. The green and red surfaces represent positive and negative signs of the MO at isovalue = 0.02 au, respectively. The blue and green surfaces represent positive and negative contributions of the spin density at an isovalue = 0.04 au. Color codes for the atoms are: gray for C, blue for N and yellow for S.


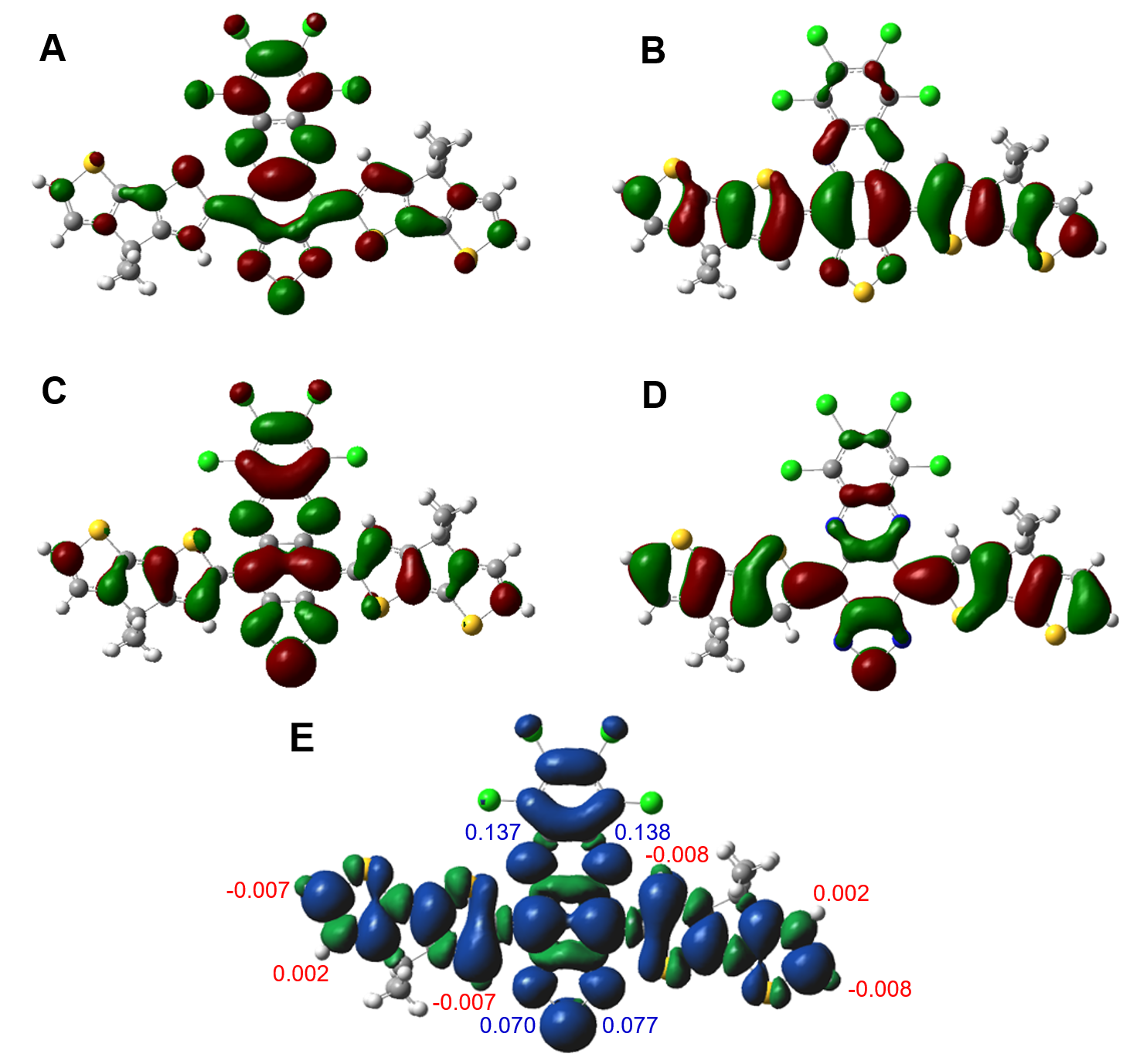


**Figure S39.** Optimized open-shell triplet ground state geometric structures of **4** calculated with (U)CAM-B3LYP/Def2-TZVP and pictorial representations of the frontier MOs. (**A**) α-SUMO and (**B**) β-SUMO, (**C**) α-SOMO and (**D**) β-SOMO, and (**E**) Spin density distribution of the open-shell triplet. The green and red surfaces represent positive and negative signs of the MO at isovalue = 0.02 au, respectively. The blue and green surfaces represent positive and negative contributions of the spin density at an isovalue = 0.04 au. Color codes for the atoms are: gray for C, blue for N and yellow for S.


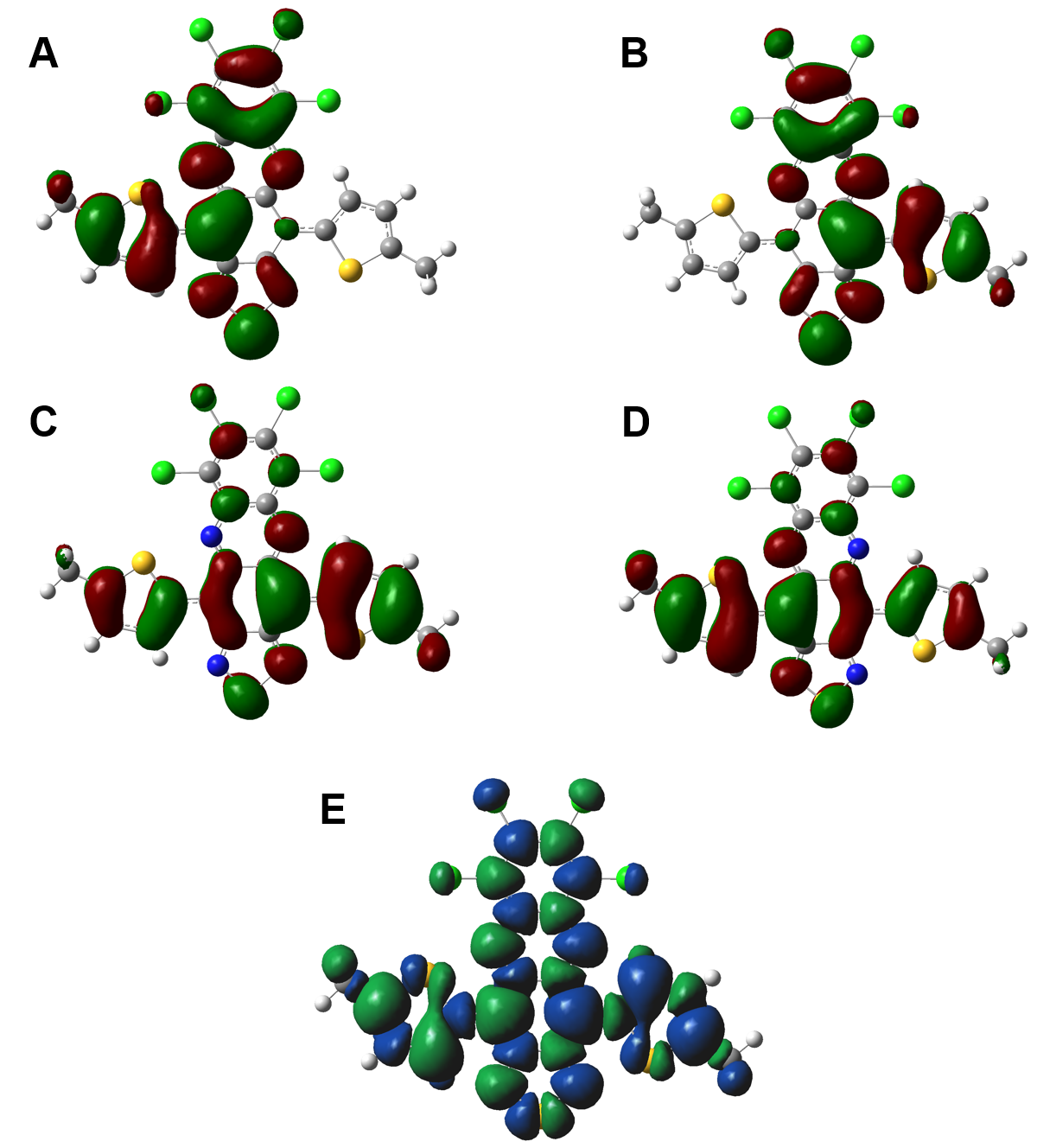


**Figure S40.** Optimized open-shell singlet ground state geometric structures of **1** calculated using broken symmetry with (U)CAM-B3LYP/Def2-TZVP and pictorial representations of the frontier MOs. (**A**) α-SUMO and (**B**) β-SUMO, (**C**) α-SOMO and (**D**) β-SOMO, and (**E**) Spin density distribution of the open-shell singlet. The green and red surfaces represent positive and negative signs of the MO at isovalue = 0.02 au, respectively. The blue and green surfaces represent positive and negative contributions of the spin density at an isovalue = 0.04 au. Color codes for the atoms are: gray for C, blue for N and yellow for S.


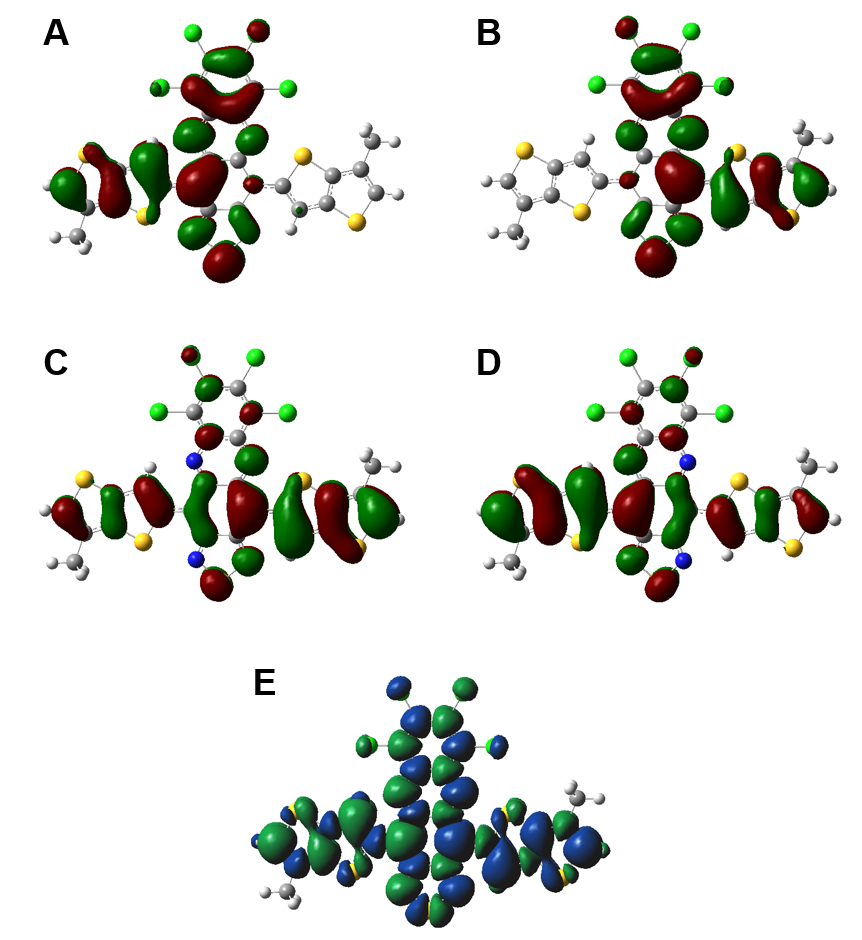


**Figure S41.** Optimized open-shell singlet ground state geometric structures of **2** calculated using broken symmetry with CAM-B3LYP/Def2-TZVP and pictorial representations of the frontier MOs. (**A**) α-SUMO and (**B**) β-SUMO, (**C**) α-SOMO and (**D**) β-SOMO, and (**E**) Spin density distribution of the open-shell singlet. The green and red surfaces represent positive and negative signs of the MO at isovalue = 0.02 au, respectively. The blue and green surfaces represent positive and negative contributions of the spin density at an isovalue = 0.04 au. Color codes for the atoms are: gray for C, blue for N and yellow for S.

**
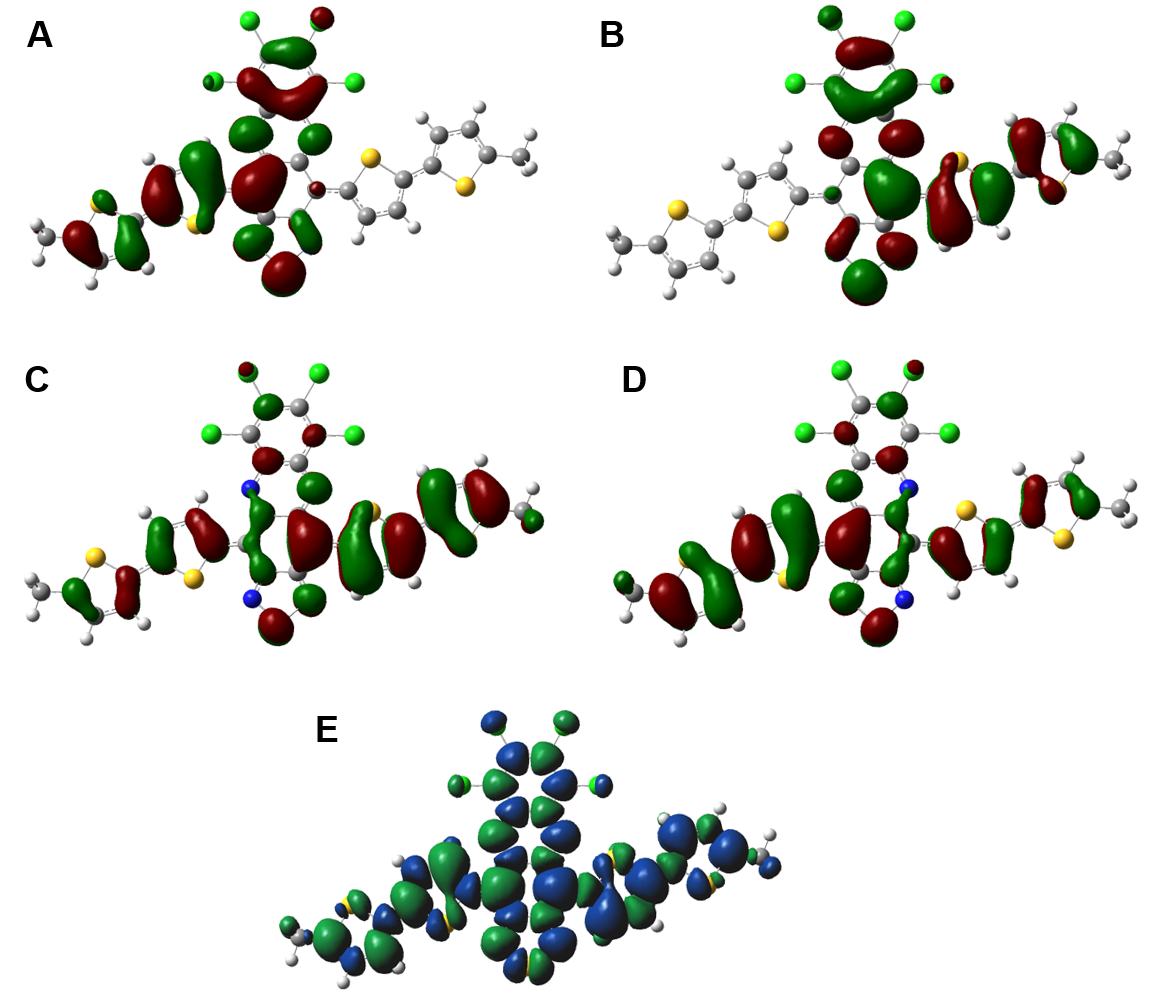
**

**Figure S42.** Optimized open-shell singlet ground state geometric structures of **3** calculated using broken symmetry with CAM-B3LYP/Def2-TZVP and pictorial representations of the frontier MOs. (**A**) α-SUMO and (**B**) β-SUMO, (**C**) α-SOMO and (**D**) β-SOMO, and (**E**) Spin density distribution of the open-shell singlet. The green and red surfaces represent positive and negative signs of the MO at isovalue = 0.02 au, respectively. The blue and green surfaces represent positive and negative contributions of the spin density at an isovalue = 0.04 au. Color codes for the atoms are: gray for C, blue for N and yellow for S.


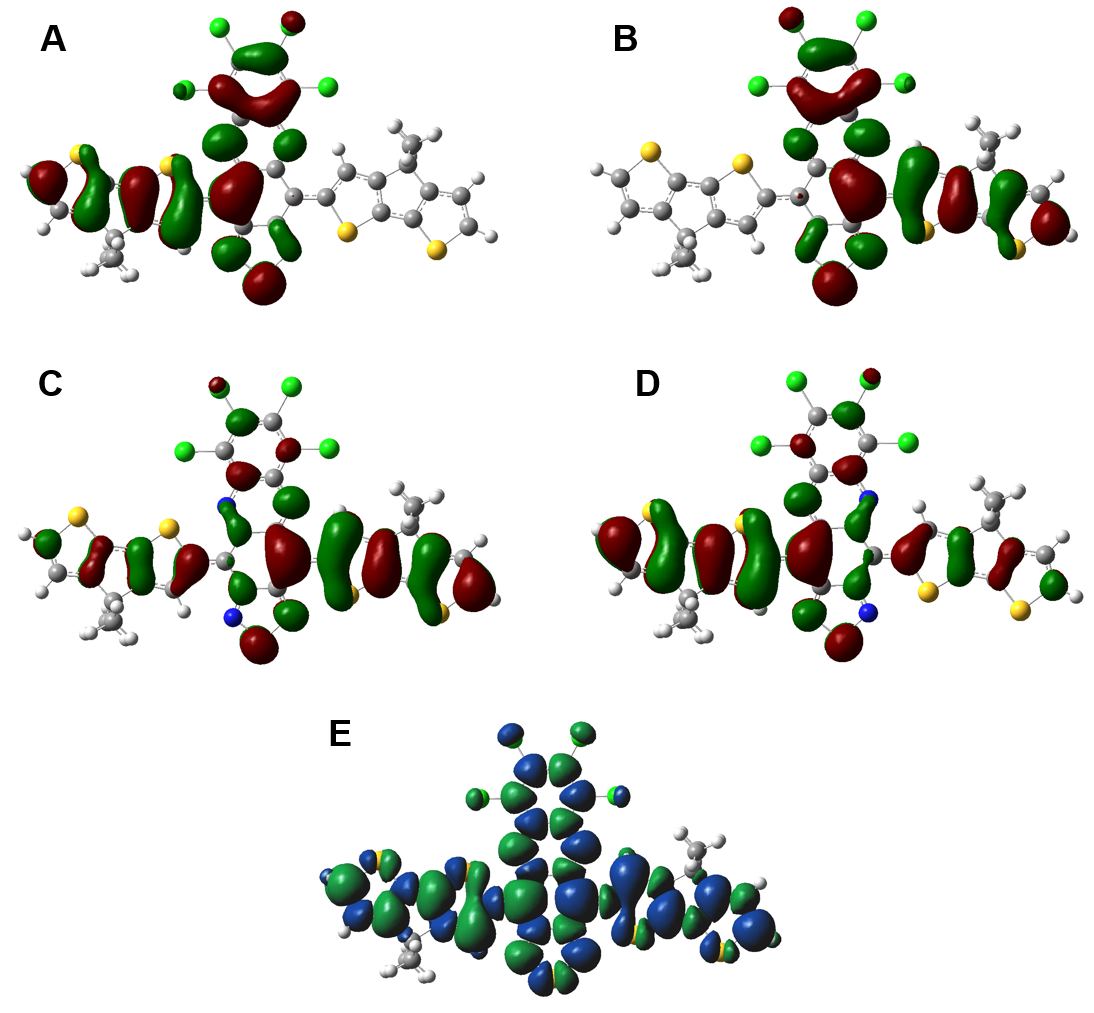


**Figure S43.** Optimized open-shell singlet ground state geometric structures of **4** calculated using broken symmetry with CAM-B3LYP/Def2-TZVP and pictorial representations of the frontier MOs. (**A**) α-SUMO and (**B**) β-SUMO, (**C**) α-SOMO and (**D**) β-SOMO, and (**E**) Spin density distribution of the open-shell singlet. The green and red surfaces represent positive and negative signs of the MO at isovalue = 0.02 au, respectively. The blue and green surfaces represent positive and negative contributions of the spin density at an isovalue = 0.04 au. Color codes for the atoms are: gray for C, blue for N and yellow for S.


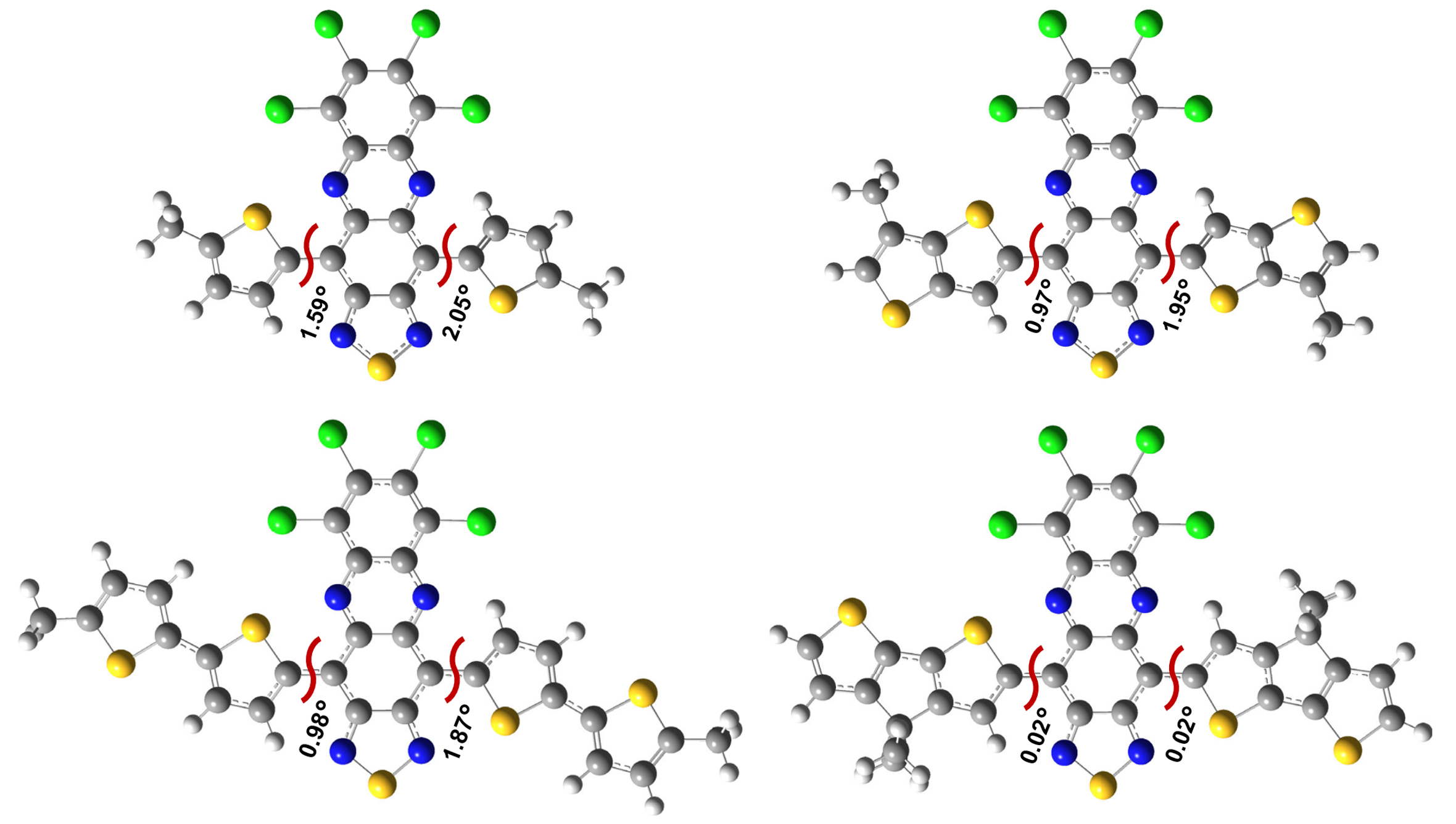


**Figure S44.** Triplet optimized geometries of **1-4** with D-A dihedral angles calculated by BS-DFT with (U)CAM-B3LYP/Def2-TZVP.


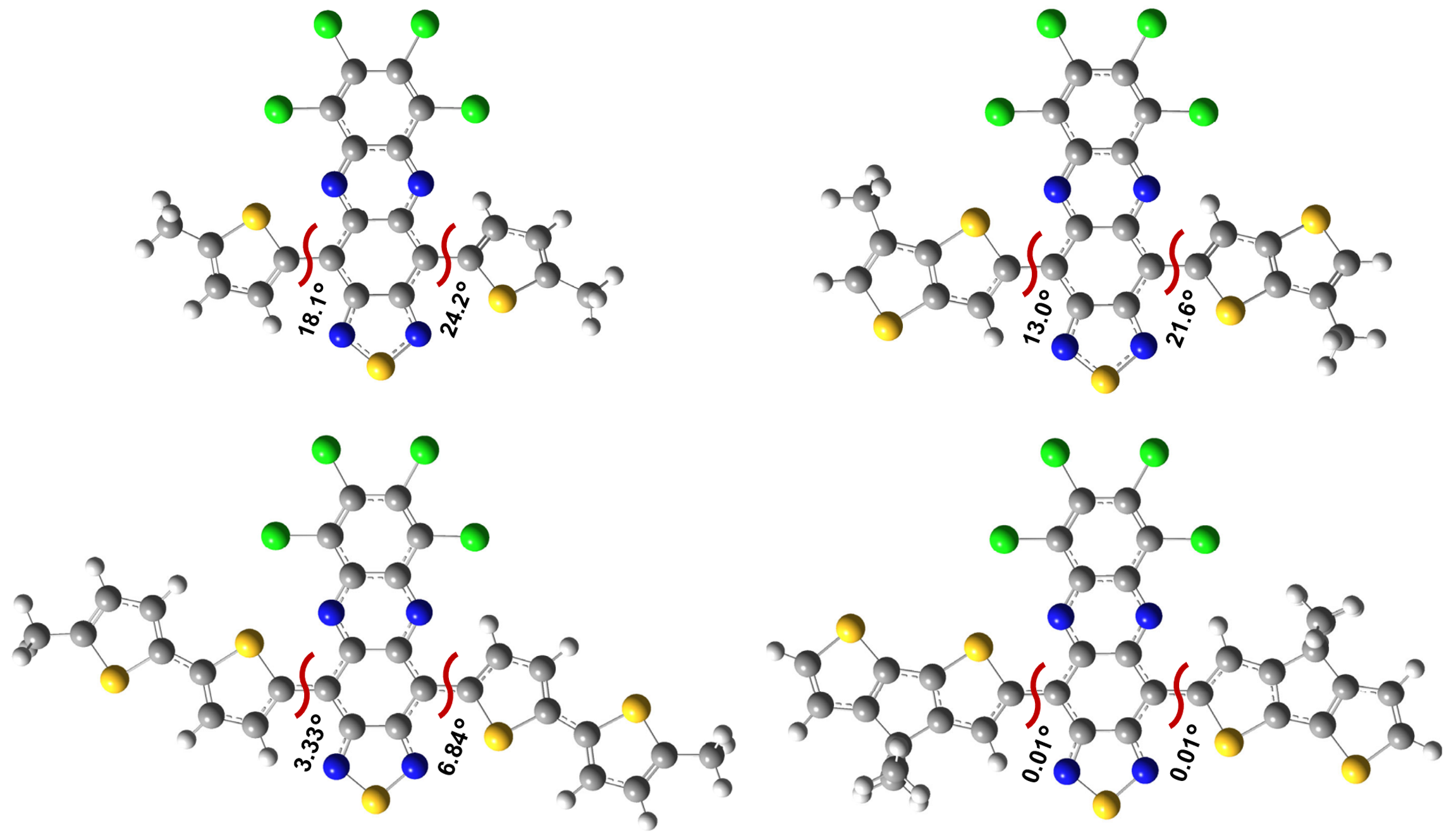


**Figure S45.** Singlet optimized geometries of **1-4** with D-A dihedral angles calculated by BS-DFT with (U)CAM-B3LYP/Def2-TZVP.

**
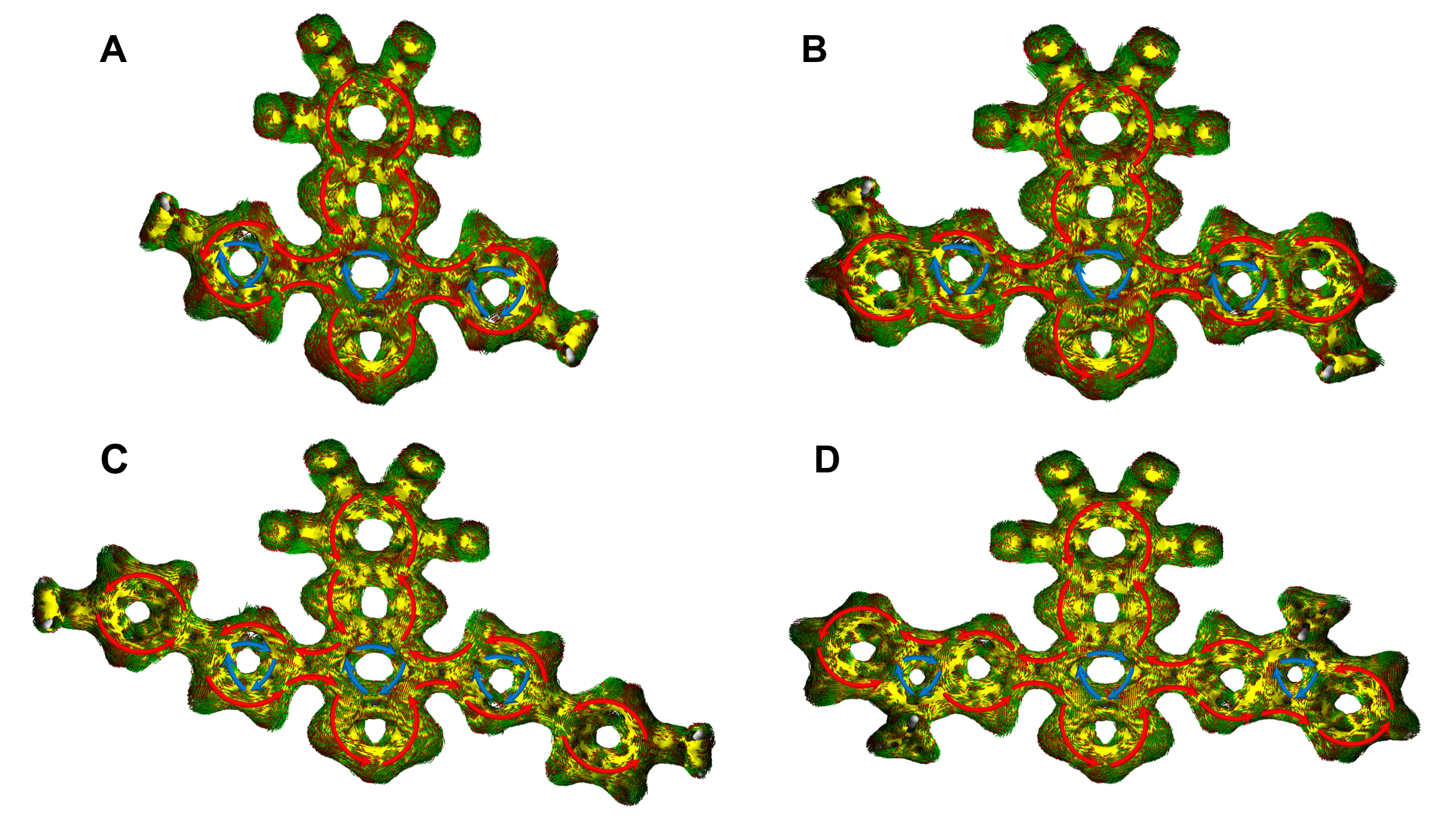
**

**Figure S46.** Anisotropy of the induced current density (ACID) of the *π*-system at CSGT-(U)CAMB3LYP/6-31G** level of theory for **1-4**. The singlet geometries were optimized by BS-DFT with CAM-B3LYP/Def2-TZVP. The current density vectors plotted on the ACID isosurface indicate ring current (clockwise, counterclockwise) and prevalent delocalization pathways present in these systems.

**
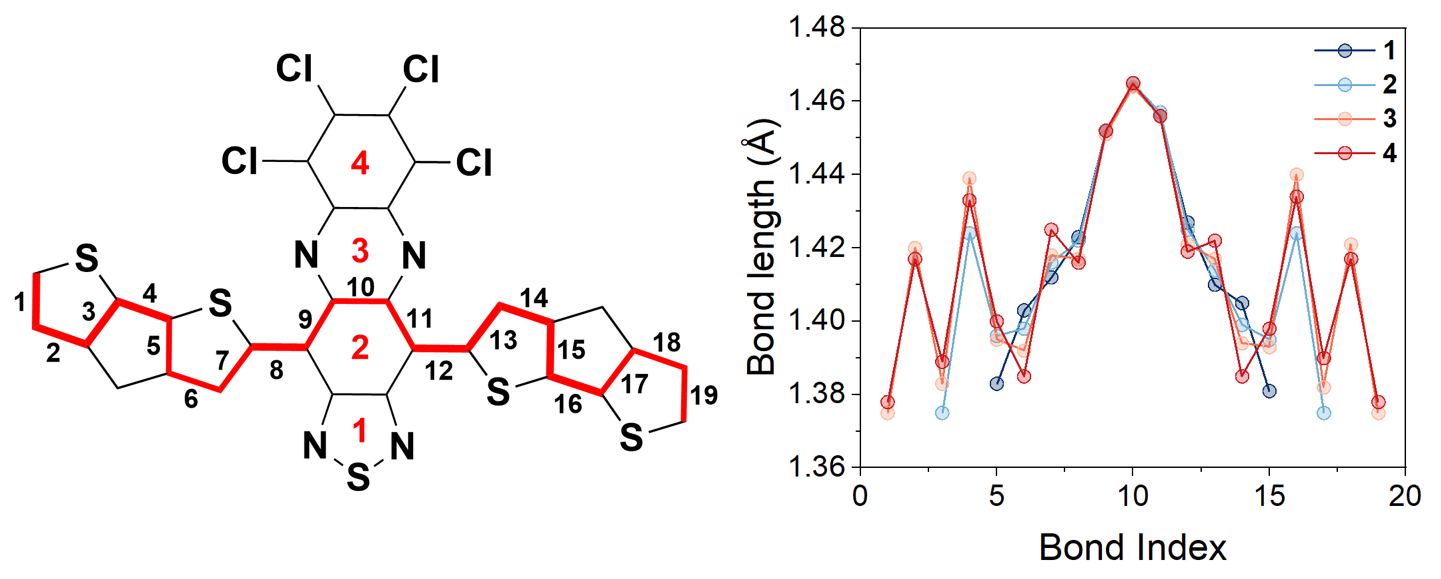
**

**Figure S47.** Bond length analysis of the triplet geometries of **1-4**.


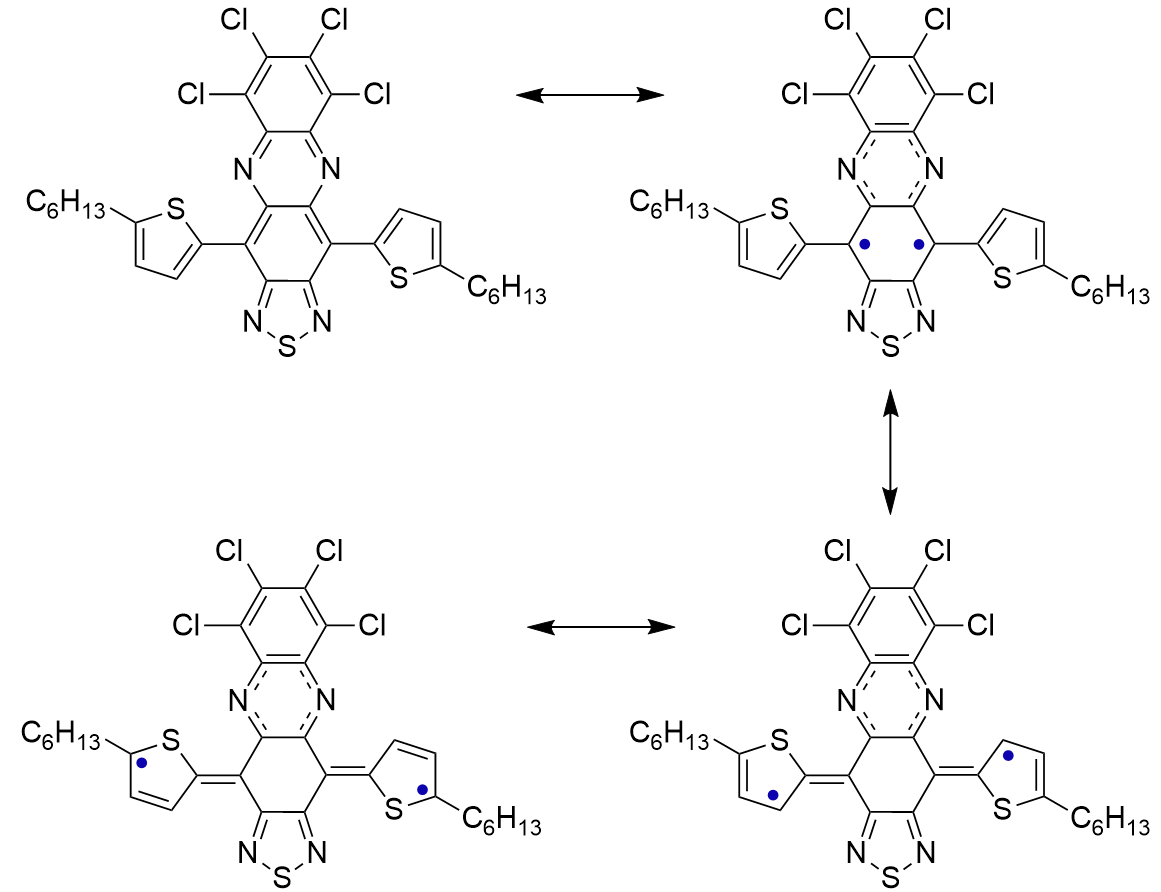


**Figure S48:** Dominant resonance structures of the singlet open-shell forms of **1**.


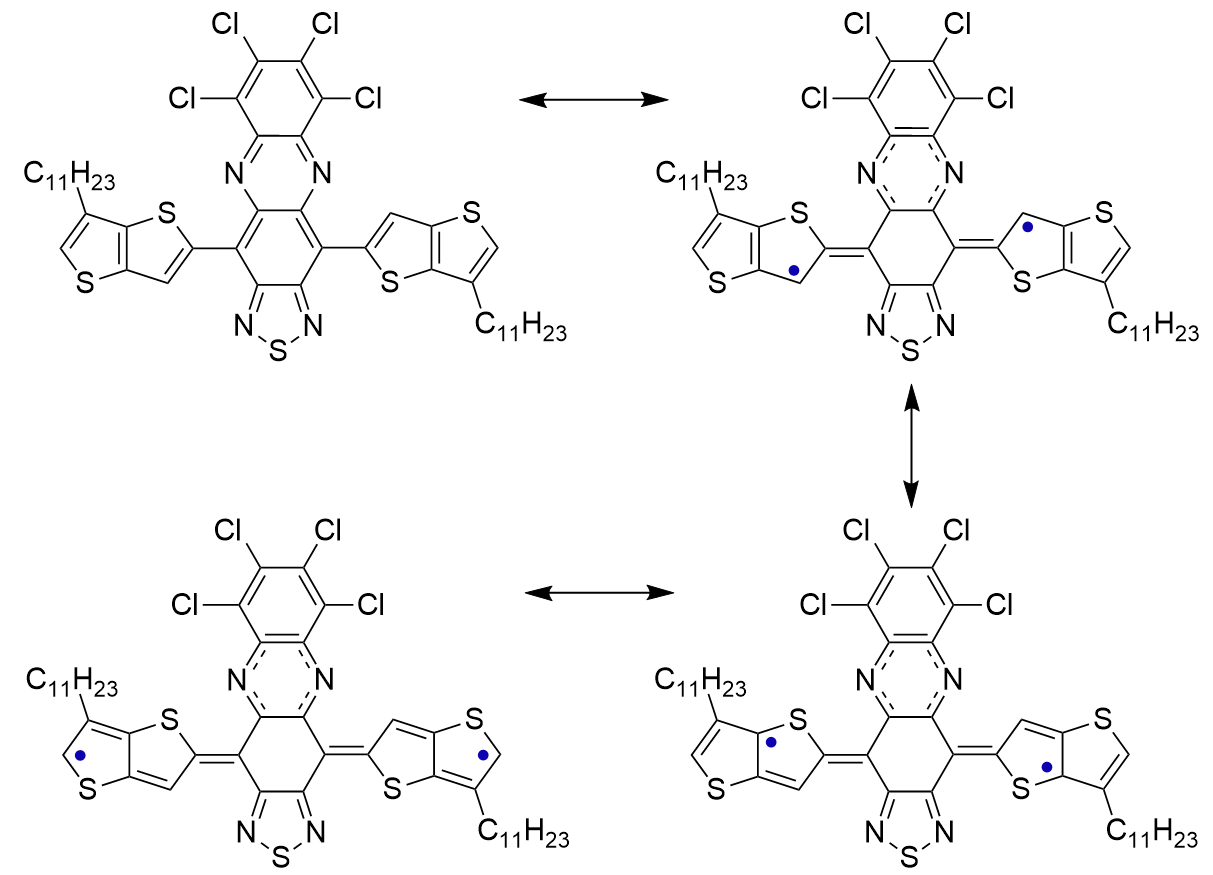


**Figure S49:** Dominant resonance structures of the singlet open-shell forms of **2.**


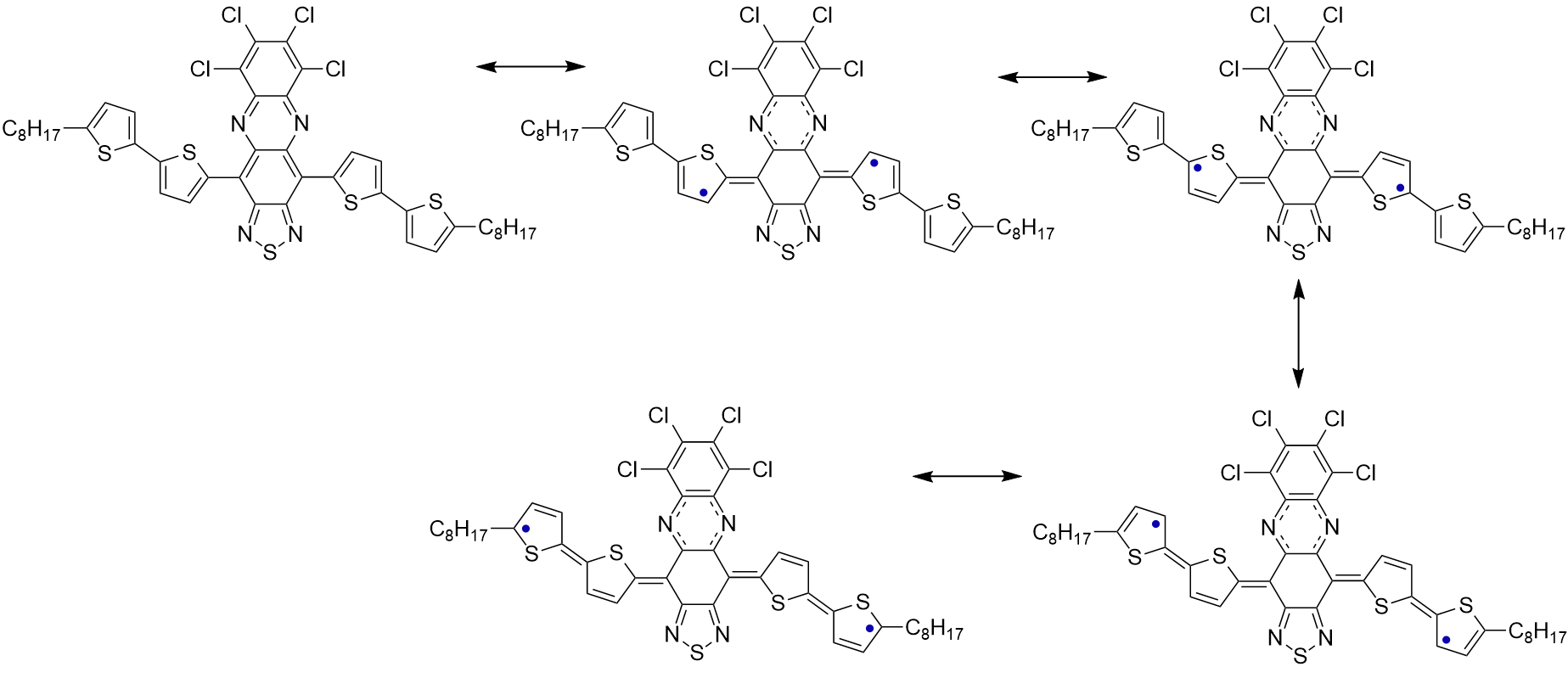


**Figure S50:** Dominant resonance structures of the singlet open-shell forms of **3.**


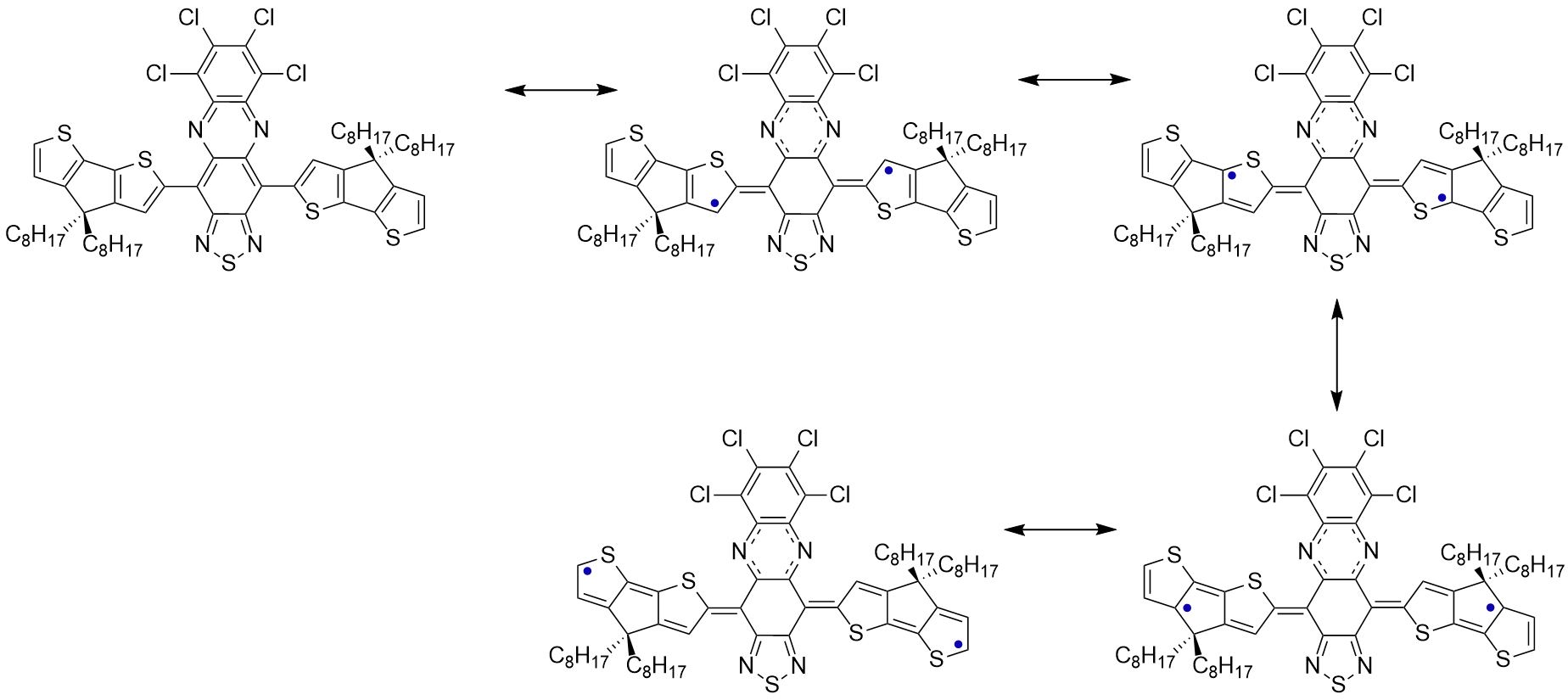


**Figure S51:** Dominant resonance structures of the singlet open-shell forms of **4**.

**5. Supplemental Tables**

**Table S1. Major electronic transitions (oscillator strength > 0.1) of the singlet-optimized geometry of 1 calculated by MRSF-TDDFT**

| Energy (nm) | Oscillator Strength | Major Contributions |
| --- | --- | --- |
| 942.8456 | 0.4995 | SOMO(α)$\to$SUMO(α) (49%) SOMO(β)$\to$SUMO(β) (49%) |
| 395.4839 | 0.4957 | HOMO-5(α) -> SUMO(α) (40%)  HOMO-5(β) -> SUMO(β) (40%) |
| 388.6652 | 0.1552 | SOMO-2(α) ->SUMO(α) (24%)  SOMO-2(β) ->SUMO(β) (24%) SOMO-3(α) -> SUMO(α) (23%)  SOMO-3(β) -> SUMO(β) (23%) |
| 355.6632 | 1.2595 | SOMO(α) -> SUMO+3(α) (42%)  SOMO(β) -> SUMO+3(β) (42%) |

**Table S2. Major electronic transitions (oscillator strength > 0.1) of the singlet-optimized geometry of 2 calculated by MRSF-TDDFT**

| Energy (nm) | Oscillator Strength | Transition MOs |
| --- | --- | --- |
| 1013.771 | 0.6394 | SOMO(α)->SUMO(α) (49%)  SOMO(β)->SUMO(β) (49%) |
| 484.5025 | 0.2113 | SOMO(α) -> LUMO(α) (49%)  SOMO(β)->LUMO(β) (49%) |
| 395.2317 | 0.4975 | HOMO-5(α) -> SUMO(α) (41%)  HOMO-5(β) -> SUMO(β) (41%) |
| 385.1637 | 0.928 | SOMO(α) -> LUMO+1(α) (46%)  SOMO(β) -> LUMO+1(β) (46%) |
| 367.4695 | 1.1322 | SOMO(α) -> LUMO+3(α) (41%)  SOMO(β) -> LUMO+1(β) (41%) |

**Table S3. Major electronic transitions (oscillator strength > 0.1) of the singlet-optimized geometry of 3 calculated by MRSF-TDDFT**

| Energy (nm) | Oscillator Strength | Transition MOs |
| --- | --- | --- |
| 1109.975 | 0.7169 | SOMO(α)->SUMO(α) (49%)  SOMO(β)->SUMO(β) (49%) |
| 504.0008 | 0.3458 | SOMO(α)->LUMO(α) (48%)  SOMO(β)->LUMO(β) (48%) |
| 423.8776 | 0.8994 | SOMO(α)->LUMO+1(α) (45%)  SOMO(β)->LUMO+1(β) (45%) |
| 398.7912 | 0.3438 | HOMO-5(α) -> LUMO(α) (33%)  HOMO-5(β) -> LUMO(β) (33%) |
| 353.2313 | 0.7524 | SOMO(α) -> LUMO+3(α) (38%)  SOMO(β) -> LUMO+3(β) (38%) |

**Table S4. Major electronic transitions (oscillator strength > 0.1) of the singlet-optimized geometry of 4 calculated by MRSF-TDDFT**

| Energy (nm) | Oscillator Strength | Transition MOs |
| --- | --- | --- |
| 1232.447 | 0.7219 | SOMO(α)->SUMO(α) (49%)  SOMO(β)->SUMO(β) (49%) |
| 529.3945 | 0.3189 | SOMO(α)-> LUMO(α) (43%)  SOMO(β)-> LUMO(β) (43%) |
| 435.6437 | 0.9116 | HOMO-3(α) -> SUMO(α) (45%);  HOMO-3(β) -> SUMO(β) (45%);  SOMO(α) -> LUMO(α) (38%)  SOMO(β) -> LUMO(β) (38%) |
| 392.7279 | 0.5818 | HOMO-5(α) -> SUMO(α) (38%)  HOMO-5(β) -> SUMO(β) (38%) |
| 364.6594 | 0.9913 | HOMO-6(α) ->SUMO(α) (46%)  SOMO(β) ->LUMO+3(β) (68%) |

**Table S5. Selected electrochemical values of 1-4.**

| **Compound** | **E_ox_^a^ (V)** | **E_red_^a^ (V)** | **E_g_^b^ (eV)** |
| --- | --- | --- | --- |
| **1** | 0.432 | -0.756 | 1.188 |
| **2** | 0.412 | -0.719 | 1.131 |
| **3** | 0.182 | -0.724 | 0.906 |
| **4** | -0.086 | -0.833 | 0.747 |

^a^Calibrated with ferrocene and corrected to the normal hydrogen electrode (NHE). ^b^Calculated from the difference between the onset of oxidation and the onset of reduction.

**Table S6. Selected electronic properties of 1-4.**

| **n^a^** | **Δ*E*_ST_^b^**  **(eV)** | **HONO^c^** | **LUNO^c^** | ***y*^d^** | **SOS-SCS**  **(eV)** | **µ_g_^e^**  **(Debye)** |
| --- | --- | --- | --- | --- | --- | --- |
| **1** | 0.154 | 1.58 | 0.42 | 0.13 | 0.007 | 2.48 |
| **2** | 0.125 | 1.55 | 0.45 | 0.16 | 0.014 | 2.70 |
| **3** | 0.093 | 1.51 | 0.49 | 0.19 | 0.040 | 3.01 |
| **4** | 0.053 | 1.45 | 0.55 | 0.25 | 0.093 | 3.39 |

*^a^*Compound *^b^*Singlet-triplet energy gap between the singlet and triplet optimized structures calculated us MRSF-TDDFT. *^c^*Natural orbital occupancies, *^d^*diradical character index (*y*) calculated from Yamaguchi's formula and *^e^*ground state dipole moment as determined at the MRSF-TDDFT level of theory. Natural orbital occupancies (HONO, LUNO) and *y* are unitless quantities. SCS: singlet closed shell; SOS: singlet open shell.

**Table S7: EPR Simulation Parameters for the spectrum of 1 in CN at 475 K.**

| **Hyperfine coupling constants (MHz)** | **Species 1** | **Species 2** |
| --- | --- | --- |
| **Spin system – pepper** | *S* = 1 | *S* = 1 |
| **^14^N HFCC (MHz)** | 15.65 | 9.87 |
| **^14^N HFCC (MHz)** | 10.04 | 6.81 |
| **^1^H HFCC (MHz)** | 1.86 | 3.53 |
| **^1^H HFCC (MHz)** | 2.26 | 1.45 |
| **Linewidth (Gaussian – mT)** | 0.00686 | 0.0110 |
| **Linewidth (Lorentzian – mT)** | 0.132 | 0.281 |
| **Weight (%)** | 63.8 | 36.2 |
| **RMSD** | 0.0068 | 0.0068 |

**Table S8: EPR Simulation Parameters for the spectrum of 2 in CN at 475 K.**

| **Parameters** | **Species 1** | **Species 2** |
| --- | --- | --- |
| **Spin system – pepper** | *S* = 1 | *S* = 1 |
| **^14^N HFCC (MHz)** | 14.41 | 15.13 |
| **^14^N HFCC (MHz)** | 8.61 | 5.62 |
| **^1^H HFCC (MHz)** | 5.95 | 4.77 |
| **Linewidth (Gaussian – mT)** | 0.0542 | 0.0192 |
| **Linewidth (Lorentzian – mT)** | 0.104 | 0.139 |
| **Weight (%)** | 77.2 | 22.8 |
| **RMSD** | 0.0098 | 0.0098 |

**Table S9. Δ*E*_ST_ values determined from solution VT EPR.**

| **Compound** | **Solvent** | **Δ*E*_ST_ (eV)** |
| --- | --- | --- |
| **1** | Toluene | -0.121 |
| **2** | Toluene | -0.120 |
| **3** | Bromobenzene | -0.100 |
| **4** | Toluene | -0.0475 |


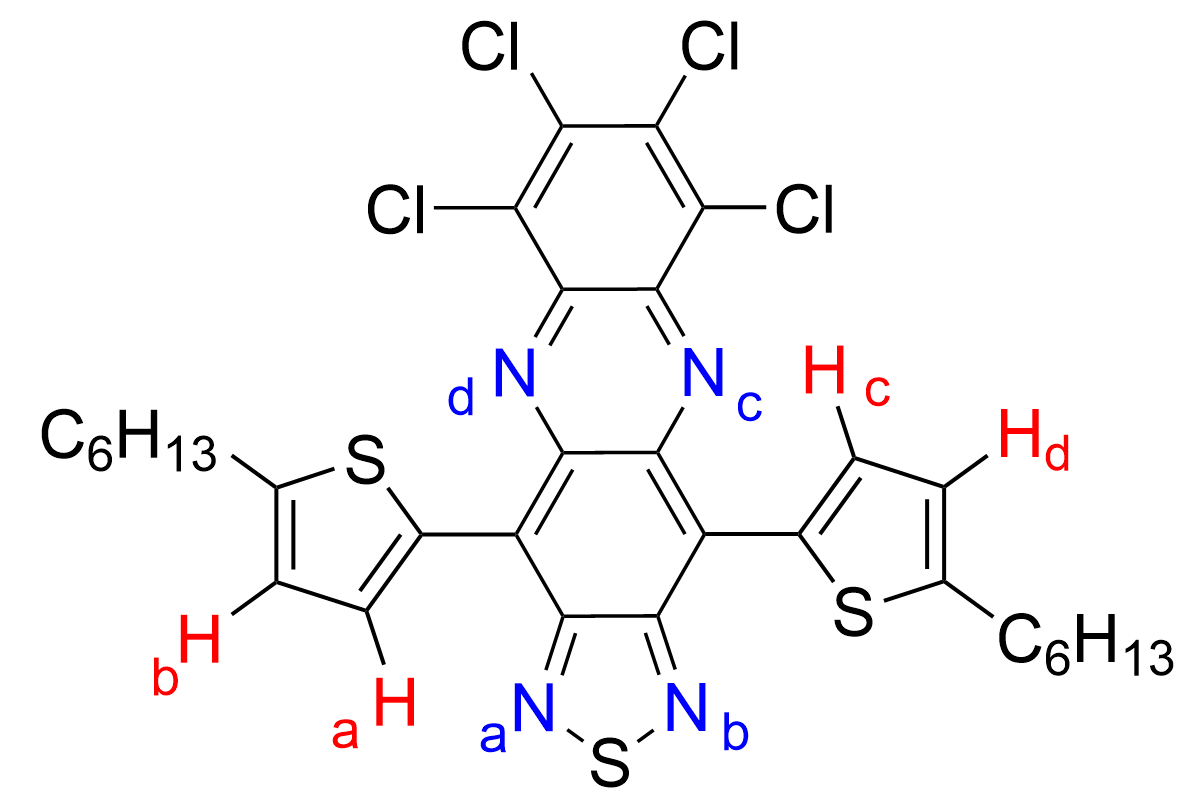


**Table S10: Hyperfine coupling constants (MHz) for 1 calculated using different functionals with the def2-TZVP basis set.**

| **Functional** | **N_a_** | **N_b_** | **N_c_** | **N_d_** | **H_a_** | **H_b_** | **H_c_** | **H_d_** |
| --- | --- | --- | --- | --- | --- | --- | --- | --- |
| **B3LYP** | 19.02 | 23.23 | 30.85 | 34.84 | -7.17 | 1.04 | -6.98 | 1.01 |
| **M062X** | 27.32 | 33.19 | 46.63 | 51.51 | -8.21 | 0.62 | -8.21 | 0.60 |
| **PB0** | 18.41 | 23.62 | 32.29 | 36.82 | -8.24 | 1.69 | -8.03 | 1.65 |


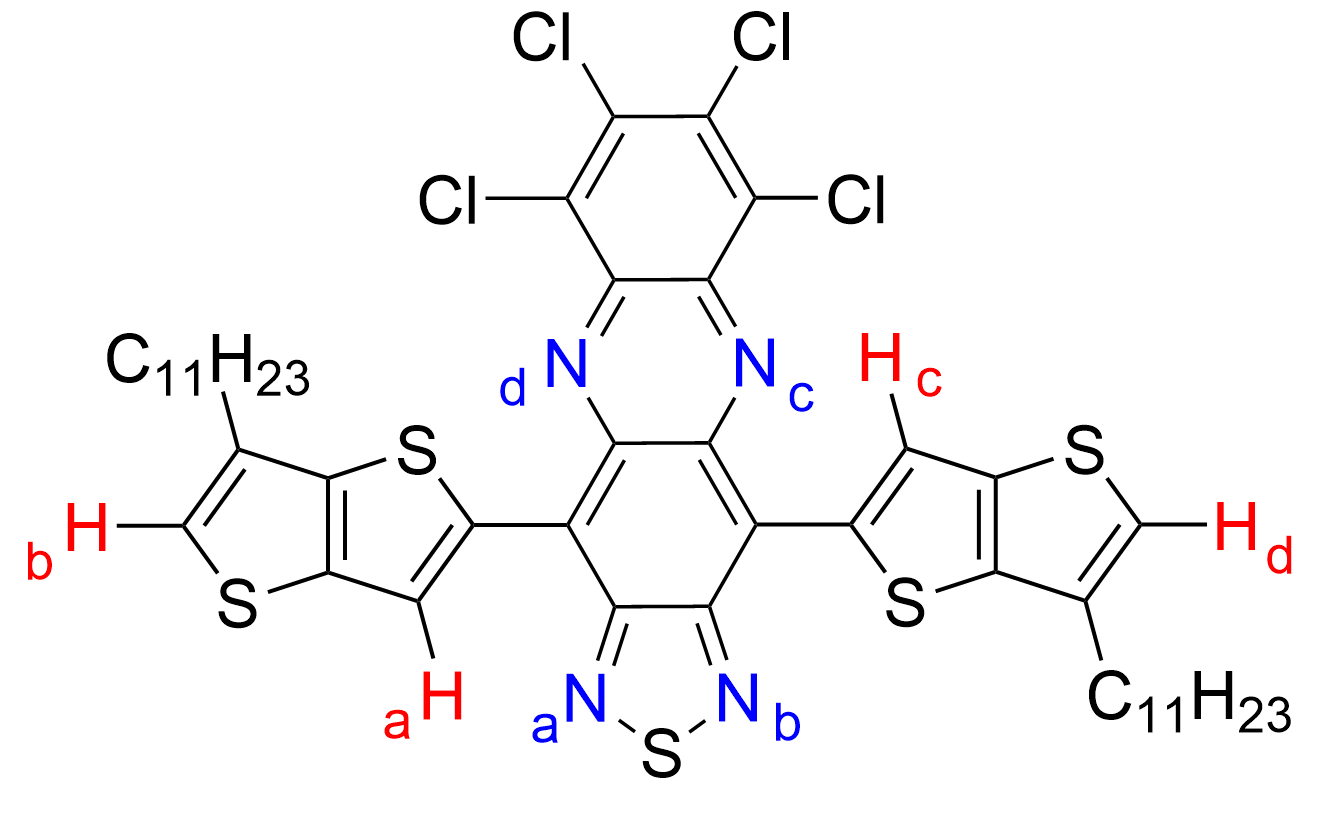


**Table S11: Hyperfine coupling constants (MHz) for 2 calculated using different functionals with the def2-TZVP basis set.**

| **Functional** | **N_a_** | **N_b_** | **N_c_** | **N_d_** | **H_a_** | **H_b_** | **H_c_** | **H_d_** |
| --- | --- | --- | --- | --- | --- | --- | --- | --- |
| **B3LYP** | 17.31 | 23.10 | 29.04 | 34.43 | -7.32 | -5.75 | -7.08 | -5.53 |
| **M062X** | 24.86 | 33.29 | 43.57 | 51.01 | -8.61 | -5.41 | -8.29 | -5.17 |
| **PB0** | 16.67 | 23.29 | 30.35 | 36.60 | -8.42 | -6.47 | -8.16 | -6.23 |


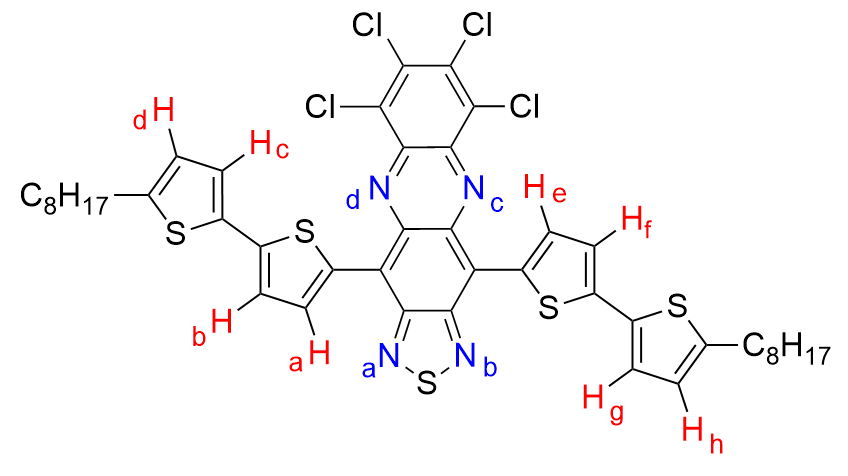


**Table S12: Hyperfine coupling constants (MHz) for 3 calculated using different functionals with the def2-TZVP basis set.**

| **Functional** | **N_a_** | **N_b_** | **N_c_** | **N_d_** | **H_a_** | **H_b_** | **H_c_** | **H_d_** | **H_e_** | **H_f_** | **H_g_** | **H_h_** |
| --- | --- | --- | --- | --- | --- | --- | --- | --- | --- | --- | --- | --- |
| **B3LYP** | 16.19 | 20.42 | 28.43 | 31.53 | -6.34 | 1.00 | -3.72 | 0.55 | -6.19 | 1.00 | -3.53 | 0.51 |
| **M062X** | 23.56 | 29.65 | 43.27 | 46.88 | -7.55 | 0.835 | -3.72 | 0.31 | -7.38 | 0.848 | -3.49 | 0.279 |
| **PB0** | 15.66 | 20.52 | 29.96 | 33.42 | -7.39 | 1.76 | -4.34 | 0.95 | -7.23 | 1.78 | -4.13 | 0.90 |


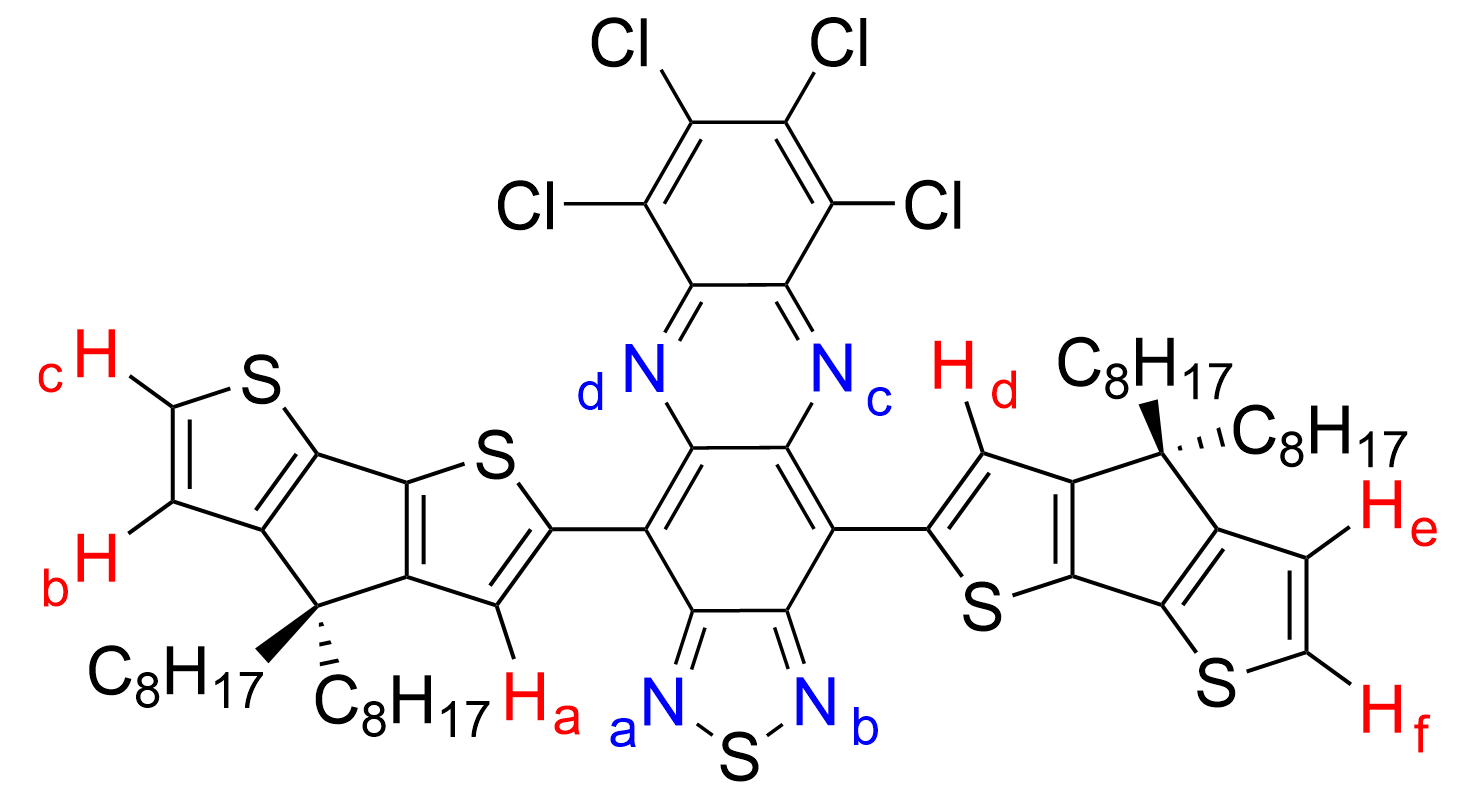


**Table S13: Hyperfine coupling constants (MHz) for 4 calculated using different functionals with the def2-TZVP basis set.**

| **Functional** | **N_a_** | **N_b_** | **N_c_** | **N_d_** | **H_a_** | **H_b_** | | **H_c_** | **H_d_** | **H_e_** | **H_f_** |
| --- | --- | --- | --- | --- | --- | --- | --- | --- | --- | --- | --- |
| **B3LYP** | 15.85 | 19.00 | 28.55 | 31.01 | -5.12 | | 1.31 | -4.79 | -5.00 | -1.27 | -4.64 |
| **PB0** | 15.47 | 19.15 | 30.05 | 32.91 | -6.09 | | 1.77 | -5.39 | -5.96 | 1.72 | -5.23 |

**Table S14. Tabulated NICS values of compound 1 singlet optimized geometry.**


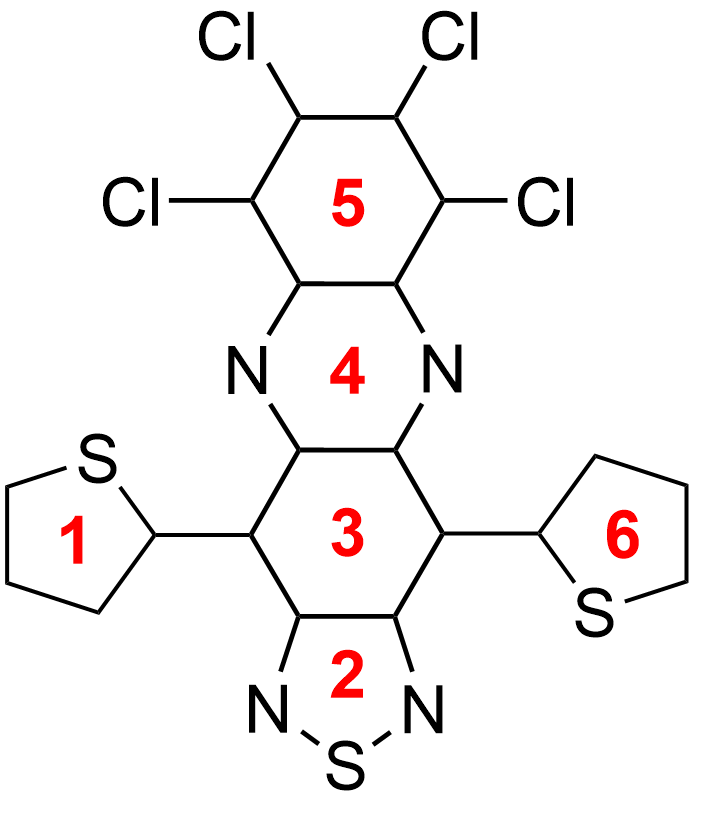


| **Ring index** | **NICS*_iso(1)_* (ppm)** |
| --- | --- |
| **1** | -7.74 |
| **2** | -14.10 |
| **3** | -9.67 |
| **4** | -10.26 |
| **5** | -6.32 |
| **6** | -6.96 |

**Table S15. Tabulated NICS values of compound 1 triplet optimized geometry.**


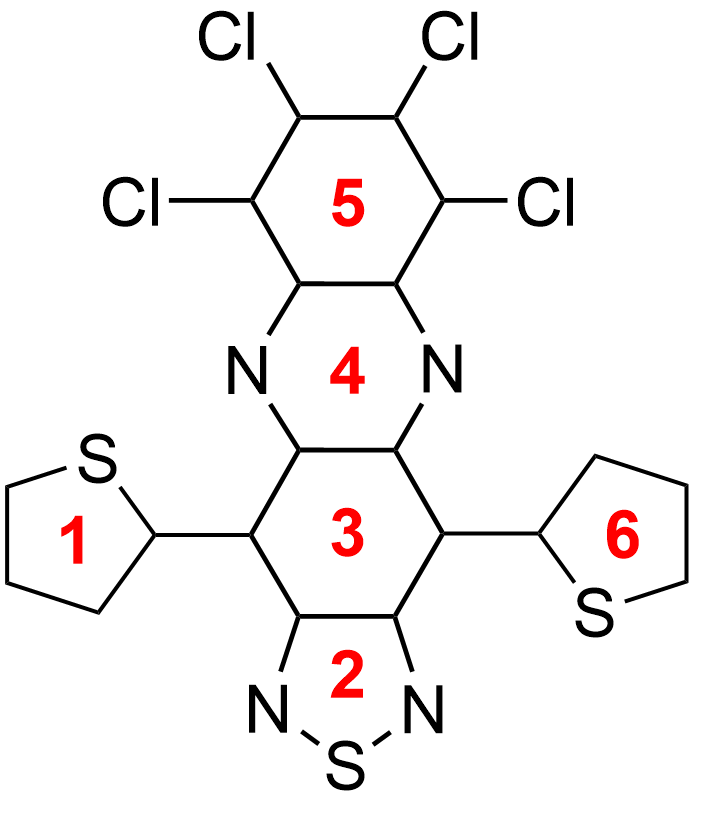


| **Ring index** | **NICS*_iso(1)_* (ppm)** |
| --- | --- |
| **1** | -6.24 |
| **2** | -8.50 |
| **3** | -0.16 |
| **4** | -5.42 |
| **5** | -7.85 |
| **6** | -5.89 |

**Table S16. Tabulated NICS values of 2 singlet optimized geometry.**

**
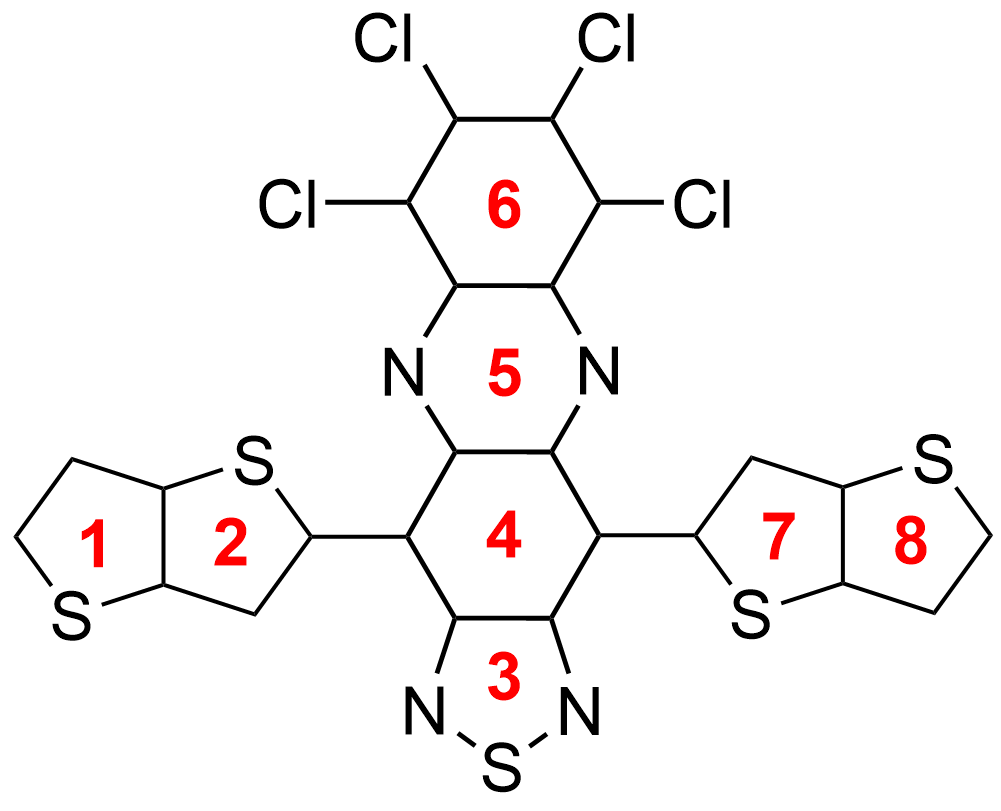
**

| **Ring index** | **NICS*_iso(1)_* (ppm)** |
| --- | --- |
| **1** | -7.86 |
| **2** | -6.94 |
| **3** | -14.54 |
| **4** | -9.08 |
| **5** | -10.16 |
| **6** | -6.23 |
| **7** | -6.60 |
| **8** | -7.76 |

**Table S17. Tabulated NICS values of 2 triplet optimized geometry.**

**
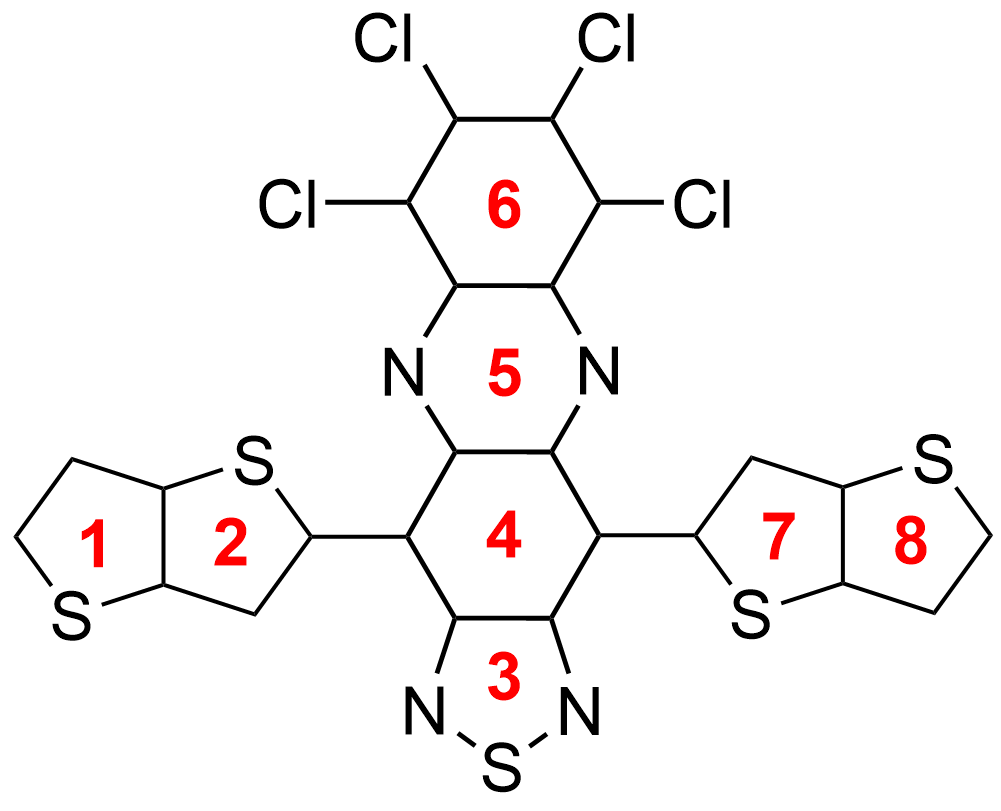
**

| **Ring index** | **NICS*_iso(1)_* (ppm)** |
| --- | --- |
| **1** | -6.60 |
| **2** | -5.30 |
| **3** | -8.74 |
| **4** | -0.06 |
| **5** | -5.44 |
| **6** | -7.80 |
| **7** | -5.11 |
| **8** | -6.39 |

**Table S18. Tabulated NICS values of 3 singlet optimized geometry.**


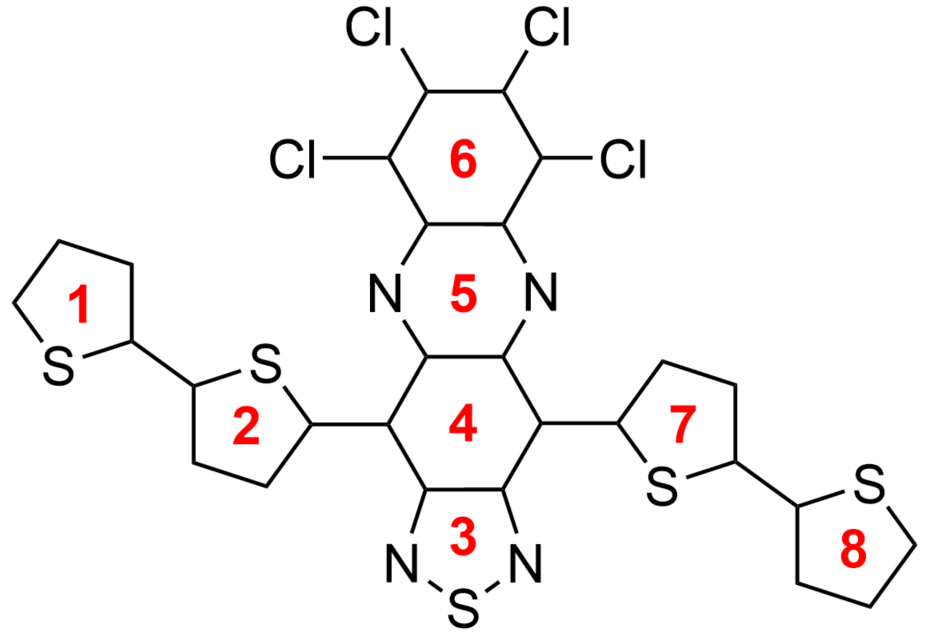


| **Ring index** | **NICS*_iso(1)_* (ppm)** | |
| --- | --- | --- |
| **1** | -7.66 |  |
| **2** | -5.90 |  |
| **3** | -14.41 |  |
| **4** | -7.98 |  |
| **5** | -9.99 |  |
| **6** | -6.89 |  |
| **7** | -5.89 |  |
| **8** | -7.67 |  |

**Table S19. Tabulated NICS values of 3 triplet optimized geometry.**


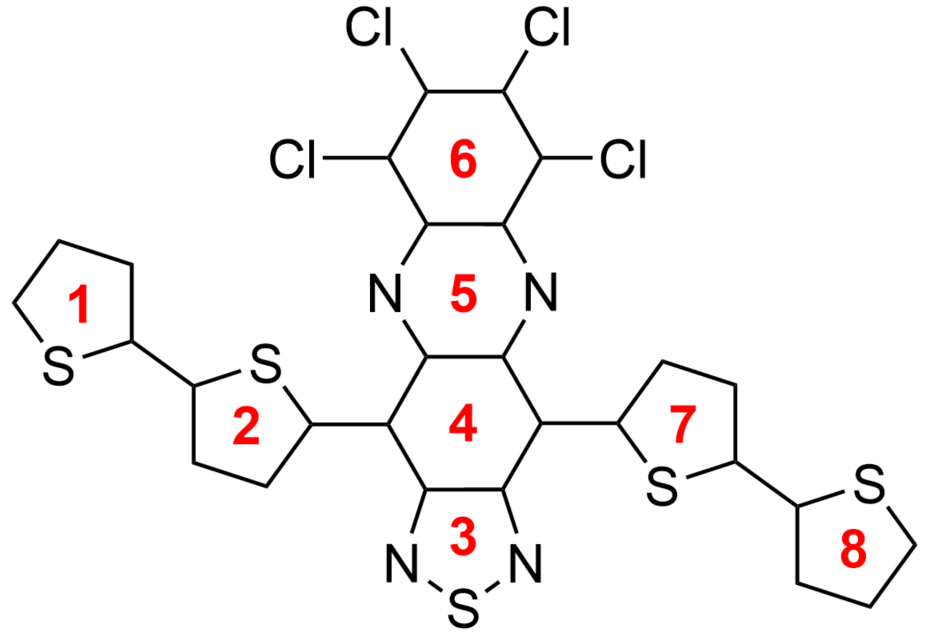


| **Ring index** | **NICS*_iso(1)_* (ppm)** | |
| --- | --- | --- |
| **1** | -7.14 |  |
| **2** | -6.46 |  |
| **3** | -9.71 |  |
| **4** | +0.09 |  |
| **5** | -5.60 |  |
| **6** | -8.16 |  |
| **7** | -5.81 |  |
| **8** | -7.98 |  |

**Table S20. Tabulated NICS values of 4 of the singlet optimized geometry.**


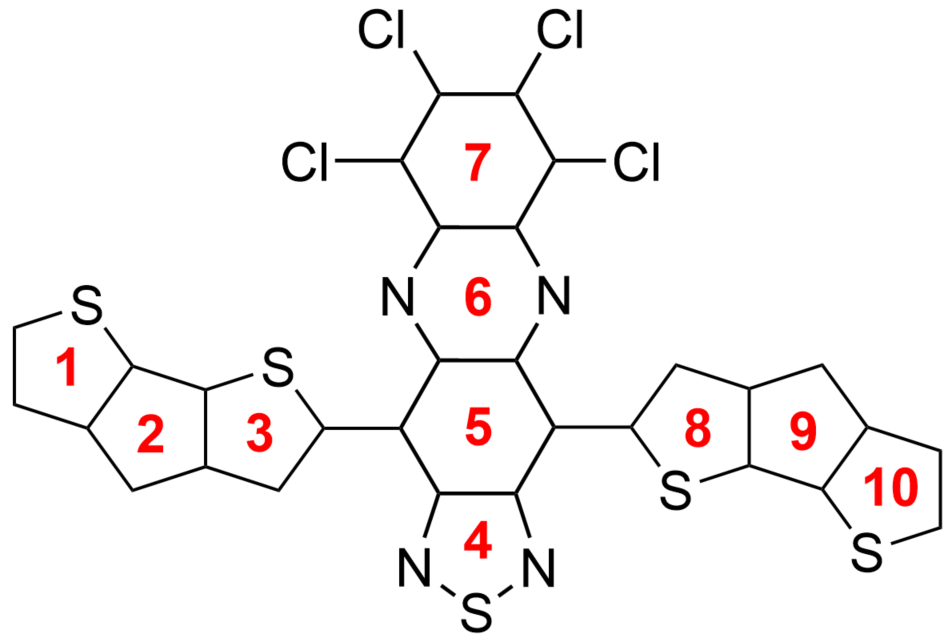


| **Ring index** | **NICS*_iso(1)_* (ppm)** |
| --- | --- |
| **1** | -7.56 |
| **2** | -1.35 |
| **3** | -5.66 |
| **4** | -14.38 |
| **5** | -5.97 |
| **6** | -7.85 |
| **7** | -7.93 |
| **8** | -5.59 |
| **9** | -1.26 |
| **10** | -7.63 |

**Table S21. Tabulated NICS values of 4 of the triplet optimized geometry.**


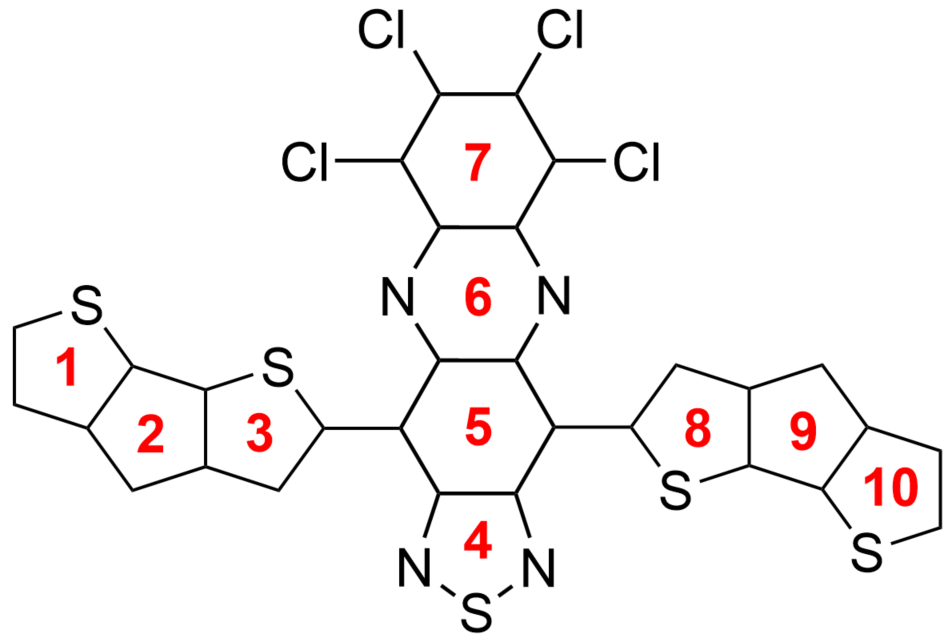


| **Ring index** | **NICS*_iso(1)_* (ppm)** |
| --- | --- |
| **1** | -8.55 |
| **2** | -1.67 |
| **3** | -5.62 |
| **4** | -8.89 |
| **5** | +0.19 |
| **6** | -5.50 |
| **7** | -7.96 |
| **8** | -5.34 |
| **9** | -1.93 |
| **10** | -8.26 |

**Table S22. Tabulated bond length values (Å) of the singlet-optimized geometry of 1 Calculated using BS-DFT using CAM-B3LYP/def2-TZVP**.


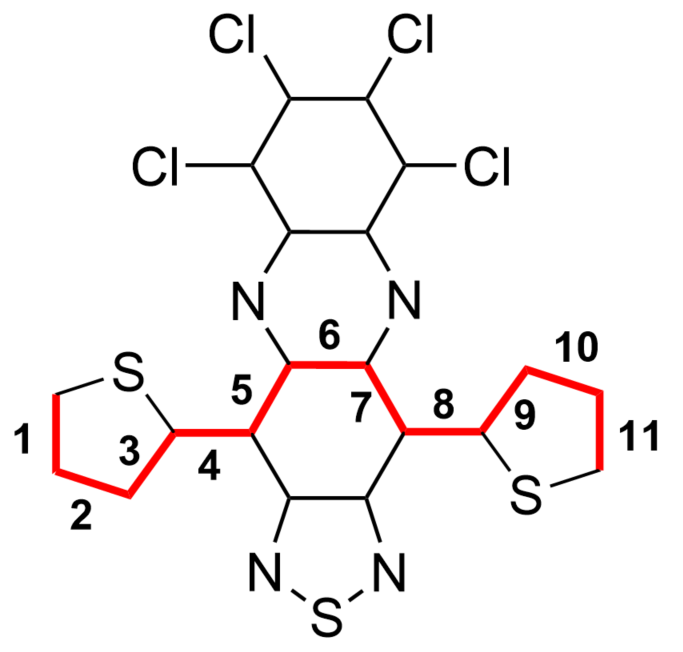


| **Bond Index** | **Length (Å)** | **Bond Index** | **Length (Å)** |
| --- | --- | --- | --- |
| **1** | 1.374 | **7** | 1.422 |
| **2** | 1.417 | **8** | 1.457 |
| **3** | 1.388 | **9** | 1.386 |
| **4** | 1.456 | **10** | 1.418 |
| **5** | 1.422 | **11** | 1.375 |
| **6** | 1.462 |  |  |

**Table S23. Tabulated bond length values (Å) of the triplet-optimized geometry of 1.**


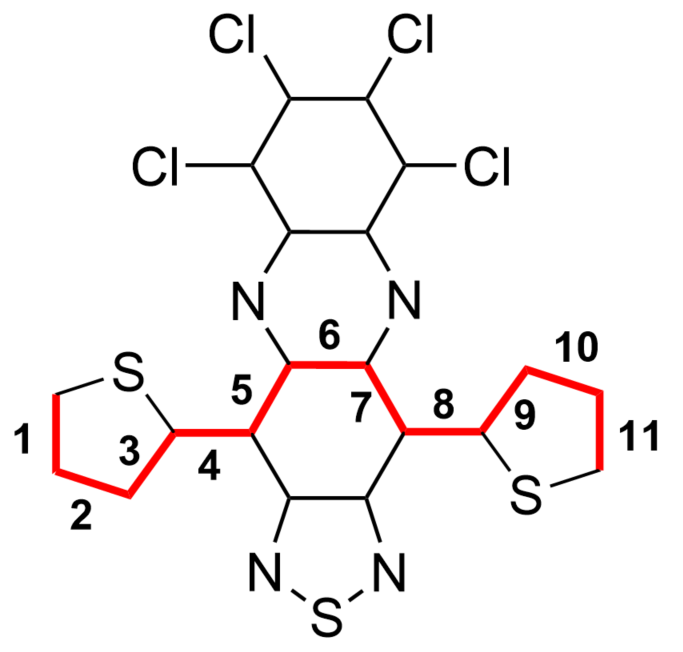


| **Bond Index** | **Length (Å)** | **Bond Index** | **Length (Å)** |
| --- | --- | --- | --- |
| **1** | 1.383 | **7** | 1.456 |
| **2** | 1.403 | **8** | 1.427 |
| **3** | 1.412 | **9** | 1.410 |
| **4** | 1.423 | **10** | 1.405 |
| **5** | 1.452 | **11** | 1.381 |
| **6** | 1.464 |  |  |

**Table S24. Tabulated bond length values (Å) of the singlet-optimized geometry of 2.**


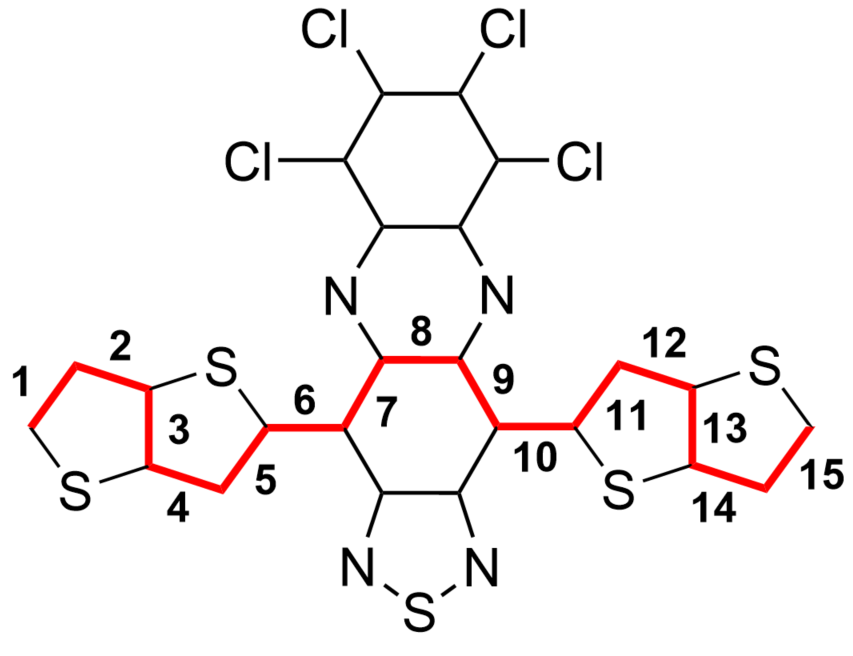


| **Bond Index** | **Length (Å)** | **Bond Index** | **Length (Å)** |
| --- | --- | --- | --- |
| **1** | 1.371 | **9** | 1.426 |
| **2** | 1.430 | **10** | 1.454 |
| **3** | 1.389 | **11** | 1.389 |
| **4** | 1.411 | **12** | 1.412 |
| **5** | 1.393 | **13** | 1.389 |
| **6** | 1.453 | **14** | 1.430 |
| **7** | 1.427 | **15** | 1.371 |
| **8** | 1.463 |  |  |

**Table S25. Tabulated bond length values (Å) of the triplet-optimized geometry of 2.**


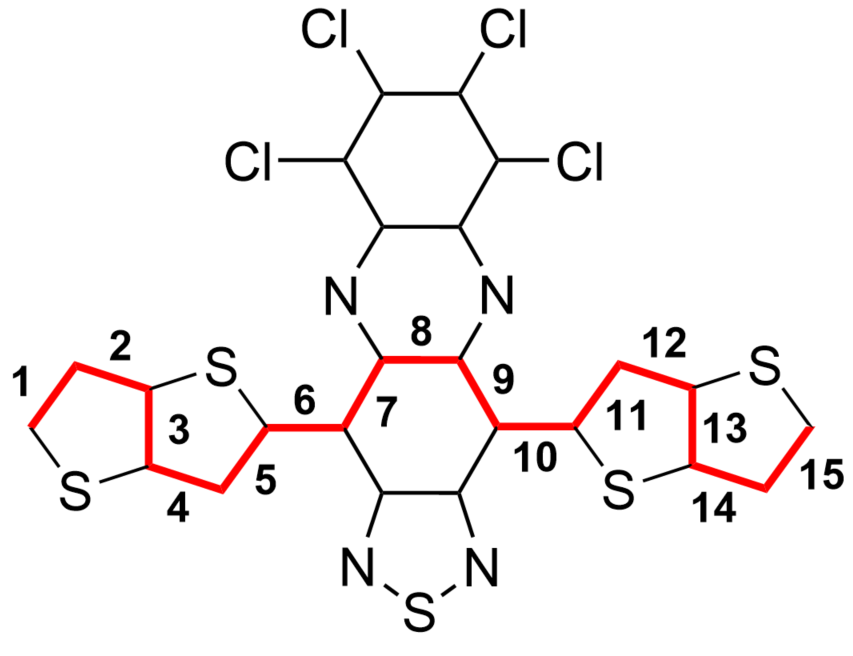


| **Bond Index** | **Length (Å)** | **Bond Index** | **Length (Å)** |
| --- | --- | --- | --- |
| **1** | 1.375 | **9** | 1.457 |
| **2** | 1.424 | **10** | 1.425 |
| **3** | 1.396 | **11** | 1.414 |
| **4** | 1.398 | **12** | 1.399 |
| **5** | 1.416 | **13** | 1.395 |
| **6** | 1.422 | **14** | 1.424 |
| **7** | 1.452 | **15** | 1.375 |
| **8** | 1.465 |  |  |

**Table S26. Tabulated bond length values (Å) the singlet-optimized geometry of 3.**


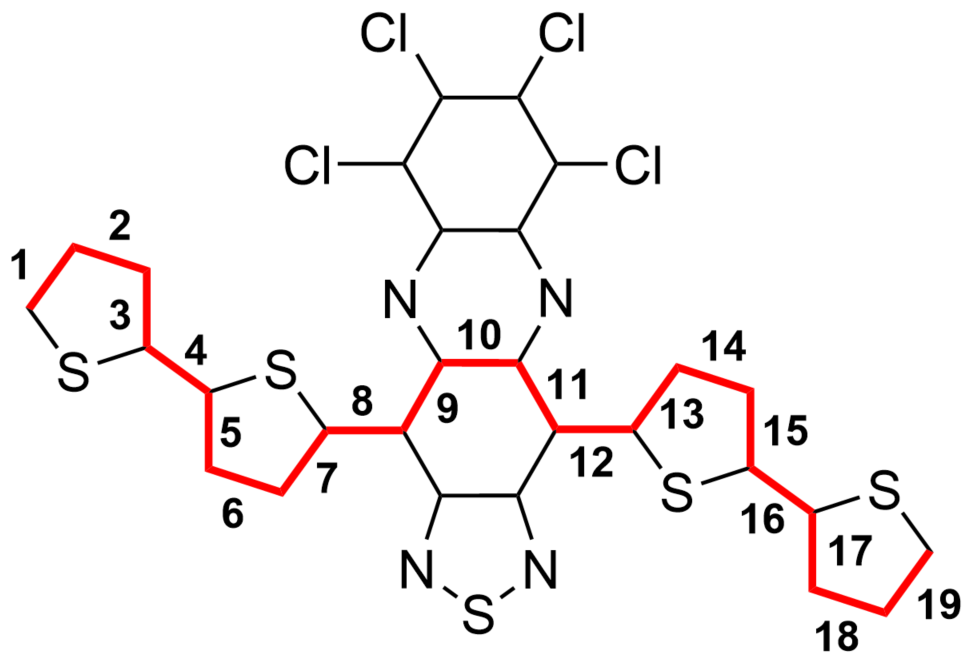


| **Bond Index** | **Length (Å)** | **Bond Index** | **Length (Å)** |
| --- | --- | --- | --- |
| **1** | 1.373 | **11** | 1.438 |
| **2** | 1.424 | **12** | 1.443 |
| **3** | 1.379 | **13** | 1.401 |
| **4** | 1.447 | **14** | 1.404 |
| **5** | 1.386 | **15** | 1.385 |
| **6** | 1.403 | **16** | 1.447 |
| **7** | 1.402 | **17** | 1.379 |
| **8** | 1.439 | **18** | 1.424 |
| **9** | 1.436 | **19** | 1.373 |
| **10** | 1.466 |  |  |

**Table S27. Tabulated bond length values (Å) the triplet-optimized geometry of 3.**


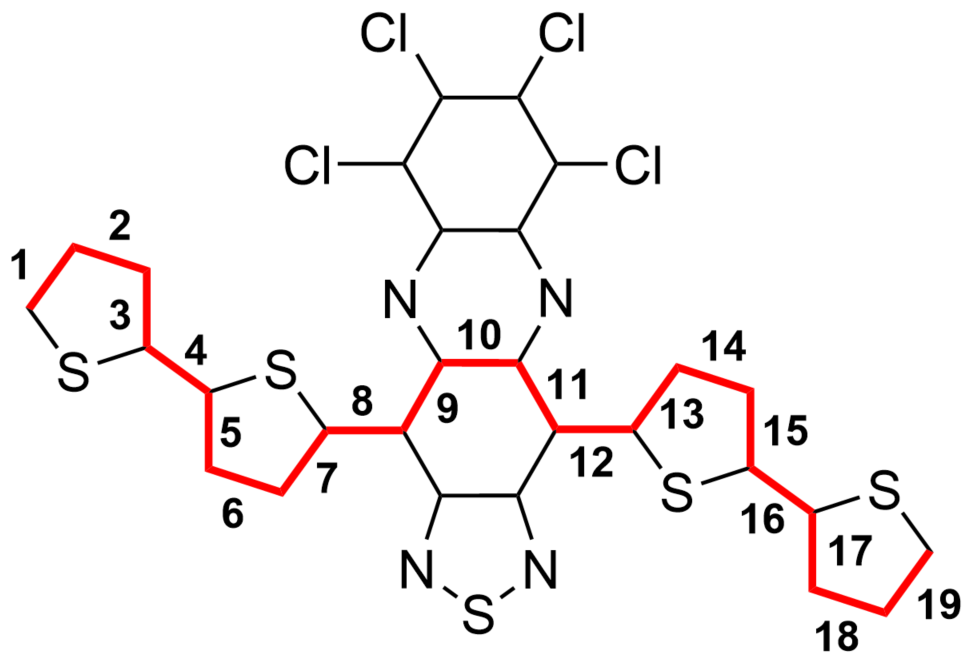


| **Bond Index** | **Length (Å)** | **Bond Index** | **Length (Å)** |
| --- | --- | --- | --- |
| **1** | 1.375 | **11** | 1.456 |
| **2** | 1.420 | **12** | 1.421 |
| **3** | 1.383 | **13** | 1.417 |
| **4** | 1.439 | **14** | 1.394 |
| **5** | 1.395 | **15** | 1.393 |
| **6** | 1.392 | **16** | 1.44 |
| **7** | 1.418 | **17** | 1.382 |
| **8** | 1.417 | **18** | 1.421 |
| **9** | 1.451 | **19** | 1.375 |
| **10** | 1.464 |  |  |

**Table S28. Tabulated bond length values (Å) of singlet-optimized geometry of 4.**


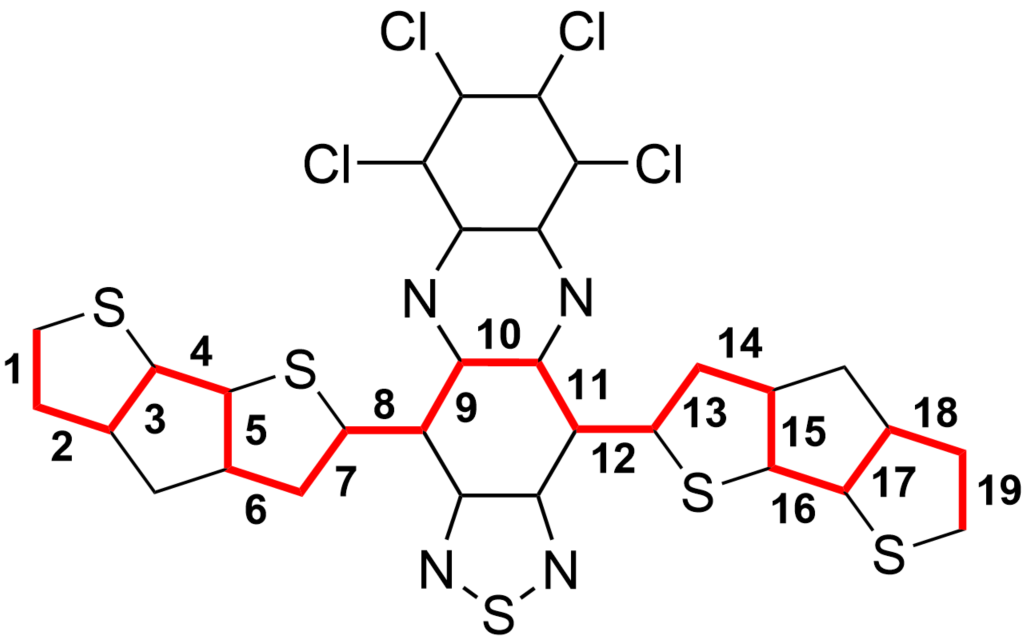


| **Bond Index** | **Length (Å)** | **Bond Index** | **Length (Å)** |
| --- | --- | --- | --- |
| **1** | 1.377 | **11** | 1.443 |
| **2** | 1.419 | **12** | 1.436 |
| **3** | 1.386 | **13** | 1.410 |
| **4** | 1.440 | **14** | 1.394 |
| **5** | 1.391 | **15** | 1.390 |
| **6** | 1.395 | **16** | 1.440 |
| **7** | 1.413 | **17** | 1.386 |
| **8** | 1.434 | **18** | 1.419 |
| **9** | 1.441 | **19** | 1.377 |
| **10** | 1.467 |  |  |

**Table S29. Tabulated bond length values (Å) of triplet-optimized geometry of 4.**


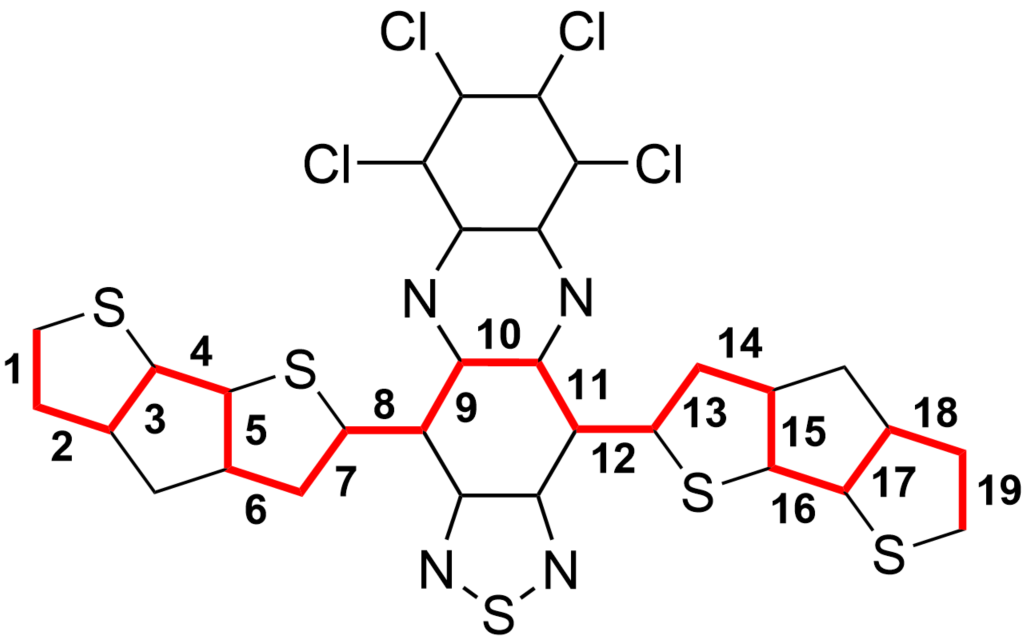


| **Bond Index** | **Length (Å)** | **Bond Index** | **Length (Å)** |
| --- | --- | --- | --- |
| **1** | 1.378 | **11** | 1.456 |
| **2** | 1.417 | **12** | 1.419 |
| **3** | 1.389 | **13** | 1.422 |
| **4** | 1.433 | **14** | 1.385 |
| **5** | 1.400 | **15** | 1.398 |
| **6** | 1.385 | **16** | 1.434 |
| **7** | 1.425 | **17** | 1.390 |
| **8** | 1.416 | **18** | 1.417 |
| **9** | 1.452 | **19** | 1.378 |
| **10** | 1.465 |  |  |

**6. Supplemental References**

1. F. Lincker, N. Delbosc, S. Bailly, R. de Bettignies, M. Billon, A. Pron, R. Demadrille, "Fluorenone‐Based Molecules for Bulk‐Heterojunction Solar Cells: Synthesis, Characterization, and Photovoltaic Properties" *Adv. Funct. Mater.* **2008**, *18*, 3444–3453.

2. S. D. Oosterhout, V. Savikhin, J. Zhang, Y. Zhang, M. A. Burgers, S. R. Marder, G. C. Bazan, M. F. Toney, "Mixing Behavior in Small Molecule:Fullerene Organic Photovoltaics" *Chem. Mater.* **2017**, *29*, 3062-3069.

3. N. Eedugurala, M. E. Steelman, P. Mahalingavelar, D. J. Adams, K. S. Mayer, C.-T. Liu, A. Benasco, G. Ma, X. Gu, M. K. Bowman, J. D. Azoulay, "Strong Acceptor Annulation Enables Control of Electronic Structure and Spin Configuration in Donor–Acceptor Conjugated Polymers" *Chem. Mat.* **2023**, *35*, 3115-3123.

4. L. Huang, N. Eedugurala, A. Benasco, S. Zhang, K. S. Mayer, D. J. Adams, B. Fowler, M. M. Lockart, M. Saghayezhian, H. Tahir, E. R. King, S. Morgan, M. K. Bowman, X. Gu, J. D. Azoulay, "Open‐Shell Donor–Acceptor Conjugated Polymers with High Electrical Conductivity" *Adv. Funct. Mater.* **2020**, *30*, 1909805.

5. S. S. Stoll, "EasySpin, a comprehensive software package for spectral simulation and analysis in EPR" **2006**, *178*, 42-55.

6. S. Mugiraneza, A. M. Hallas, "Tutorial: a beginner’s guide to interpreting magnetic susceptibility data with the Curie-Weiss law" *Communications Physics* **2022**, *5*.

7. D. J. Adams, K. S. Mayer, M. Steelman, J. D. Azoulay, "Magnetic Characterization of Open-Shell Donor–Acceptor Conjugated Polymers" *J. Phys. Chem. C* **2022**, *126*, 5701-5710.

8. S. Lee, E. E. Kim, H. Nakata, S. Lee, C. H. Choi, "Efficient implementations of analytic energy gradient for mixed-reference spin-flip time-dependent density functional theory (MRSF-TDDFT)" *J. Chem. Phys.* **2019**, *150*, 184111.

9. Y. Shao, H.-G. M., A. I. Krylov, "The spin–flip approach within time-dependent density functional theory: Theory and applications to diradicals" *J. Chem. Phys.* **2003**, *118*, 4807-4818.

10. M. J. Frisch, G. W. Trucks, H. B. Schlegel, G. E. Scuseria, M. A. Robb, J. R. Cheeseman, G. Scalmani, V. Barone, G. A. Petersson, H. Nakatsuji, X. Li, M. Caricato, A. V. Marenich, J. Bloino, B. G. Janesko, R. Gomperts, B. Mennucci, H. P. Hratchian, J. V. Ortiz, A. F. Izmaylov, J. L. Sonnenberg, D. Williams-Young, F. Ding, F. Lipparini, F. Egidi, J. Goings, B. Peng, A. Petrone, T. Henderson, D. Ranasinghe, V. G. Zakrzewski, J. Gao, N. Rega, G. Zheng, W. Liang, M. Hada, M. Ehara, K. Toyota, R. Fukuda, J. Hasegawa, M. Ishida, T. Nakajima, Y. Honda, O. Kitao, H. Nakai, T. Vreven, K. Throssell, J. J. A. Montgomery, J. E. Peralta, F. Ogliaro, M. J. Bearpark, J. J. Heyd, E. N. Brothers, K. N. Kudin, V. N. Staroverov, T. A. Keith, R. Kobayashi, J. Normand, K. Raghavachari, A. P. Rendell, J. C. Burant, S. S. Iyengar, J. Tomasi, M. Cossi, J. M. Millam, M. Klene, C. Adamo, R. Cammi, J. W. Ochterski, R. L. Martin, K. Morokuma, O. Farkas, J. B. Foresman, D. J. Fox., *Gaussian 16, Revision A.03, Gaussian Inc., Wallingford CT* **2016**.

11. A. D. Becke, "A new mixing of Hartree-Fock and local density-functional theories" *J. Chem. Phys.* **1993**, *98*, 1372-1377.

12. F. Weigend, R. Ahlrichs, "Balanced basis sets of split valence, triple zeta valence and quadruple zeta valence quality for H to Rn: Design and assessment of accuracy" *Phys. Chem. Chem. Phys.* **2005**, *7*, 3297-3305.

13. T. Yanai, D. P. Tew, N. C. Handy, "A new hybrid exchange–correlation functional using the Coulomb-attenuating method (CAM-B3LYP)" *Chem. Phys. Lett.* **2004**, *393*, 51-57.

14. G. M. J. Barca, C. Bertoni, L. Carrington, D. Datta, N. De Silva, J. E. Deustua, D. G. Fedorov, J. R. Gour, A. O. Gunina, E. Guidez, T. Harville, S. Irle, J. Ivanic, K. Kowalski, S. S. Leang, H. Li, W. Li, J. J. Lutz, I. Magoulas, J. Mato, V. Mironov, H. Nakata, B. Q. Pham, P. Piecuch, D. Poole, S. R. Pruitt, A. P. Rendell, L. B. Roskop, K. Ruedenberg, T. Sattasathuchana, M. W. Schmidt, J. Shen, L. Slipchenko, M. Sosonkina, V. Sundriyal, A. Tiwari, J. L. Galvez Vallejo, B. Westheimer, M. Wloch, P. Xu, F. Zahariev, M. S. Gordon, "Recent developments in the general atomic and molecular electronic structure system" *J. Chem. Phys.* **2020**, *152*, 154102.

15. V. Pomogaev, S. Lee, S. Shaik, M. Filatov, C. H. Choi, "Exploring Dyson's Orbitals and Their Electron Binding Energies for Conceptualizing Excited States from Response Methodology" *J. Phys. Chem. Lett.* **2021**, *12*, 9963-9972.

16. J. V. Ortiz, "Dyson-orbital concepts for description of electrons in molecules" *J. Chem. Phys.* **2020**, *153*, 070902.

17. S. K. Yamanaka, Noro, T., Yamaguchi, K. , "Heisenberg model for radical reactions. Part 3. Direct exchange coupling between transition metal ions and triplet methylene" *J. Mol. Struct.* **1994**, *310*, 185-196.

18. M. Nakano, R. Kishi, S. Ohta, A. Takebe, H. Takahashi, S. Furukawa, T. Kubo, Y. Morita, K. Nakasuji, K. Yamaguchi, K. Kamada, K. Ohta, B. Champagne, E. Botek, "Origin of the enhancement of the second hyperpolarizability of singlet diradical systems with intermediate diradical character" *J. Chem. Phys.* **2006**, *125*, 074113.
